# The tribe Phanerotomini (Hymenoptera, Braconidae, Cheloninae) of the Arabian Peninsula, with special reference to the United Arab Emirates and Yemen

**DOI:** 10.3897/zookeys.1014.60426

**Published:** 2021-02-03

**Authors:** Cornelis van Achterberg

**Affiliations:** 1 Naturalis Biodiversity Center, Postbus 9517, 2300 RA Leiden, the Netherlands Naturalis Biodiversity Center Leiden Netherlands

**Keywords:** Key, new record, new species, new synonymy, *
Phanerotoma
*, *
Phanerotomella
*, Saudi Arabia, United Arab Emirates, Yemen

## Abstract

For the first time the tribe Phanerotomini (Braconidae, Cheloninae) of the Arabian Peninsula is revised, illustrated by colour photographs and keyed. It resulted in twenty-one new species (of which 20 species belong to the genus *Phanerotoma* and representing 75% of the reported species): *Phanerotomella yemenitica***sp. nov.**, *Phanerotoma angusticrus***sp. nov.**, *P. artocornuta***sp. nov.**, *P. aspidiota***sp. nov.**, *P. brunneivena***sp. nov.**, *P. caudatoides***sp. nov.**, *P. glabritemporalis***sp. nov.**, *P. granulata***sp. nov.**, *P. ejuncida***sp. nov.**, *P. hellyeri***sp. nov.**, *P. latifemorata***sp. nov.**, *P. lepta***sp. nov.**, *P. longivena***sp. nov.**, *P. mesocellata***sp. nov.**, *P. microdonta***sp. nov.**, *P. micrommata***sp. nov.**, *P. sculptilis***sp. nov.**, *P. signifera***sp. nov.**, *P. spuriserrata***sp. nov.**, *P. stenochora***sp. nov.**, and *P. vanharteni***sp. nov.** Reported as new for United Arab Emirates and Yemen are *Phanerotoma graciloides* van Achterberg, 1990, *P. masiana* Fahringer, 1934, and *P. leucobasis* Kriechbaumer, 1894 (the latter also for Saudi Arabia), for United Arab Emirates *P. ocularis* Kohl, 1906, and *P. robusta* Zettel, 1988, and for Yemen *P. bilinea* Lyle, 1924, *P. flavivena* Edmardash & Gadallah, 2019, and *P. permixtellae* Fischer, 1968. *Phanerotoma caboverdensis* Hedqvist, 1965, **syn. nov.** is synonymised with *P. leucobasis* Kriechbaumer, 1894.

## Introduction

Tony van Harten brought together the largest known collection of the tribe Phanerotomini Baker, 1926 (Hymenoptera, Braconidae, Cheloninae) from the Arabian Peninsula, originating from many localities in the United Arab Emirates and Yemen. The Cheloninae is one of the more easily recognizable groups of the Braconidae because of the metasomal carapace (Figs 282, 291, 387) and the presence of the complete postpectal carina (Figs 250, 276). It is a moderately large subfamily with almost 1,500 described species worldwide and is divided into four tribes, of which three are present on the Arabian Peninsula: Chelonini Foerster, 1863 (1170+ spp.), Phanerotomini Baker, 1926 (320+ spp.), and Adeliini Viereck, 1918 (30+ spp.).

This paper is an attempt to give an overview of the large diversity of the tribe Phanerotomini in the Arabian Peninsula, with special reference to the United Arab Emirates and Yemen. The tribe has a world-wide distribution but occurs predominantly in the subtropical and tropical areas. The most common genus in the Arabian Peninsula is *Phanerotoma* Wesmael, 1838, a large genus of usually largely yellowish and often medium-sized species that are commonly attracted to light. *Phanerotoma* contains solitary ovo-larval koinobiont endoparasitoids of mainly Pyralidae and Tortricidae (Lepidoptera) and to a lesser extent of other Lepidoptera (Blastobasidae, Coleophoridae, Cosmopterygidae, Gelechiidae, Gracillariidae, Lasiocampidae, Lymantriidae, Noctuidae, Nolidae, Oecophoridae and Yponomeutidae) (Yu et al. 2016). The preferred use of Pyralidae as host seems to be a special development within the Phanerotomini. In other Cheloninae the use of Pyralidae as hosts is exceptional; it is only known for a few species of the genus *Chelonus* Panzer (Jones, 1985).

In addition, a new species from Yemen of the medium-sized genus *Phanerotomella* Szépligeti, 1900, is described and the genus is new for the Arabian Peninsula. The biology of this genus is largely unknown, only one species is known to be a parasitoid of Oecophoridae (Yu et al. 2016). For a summary of the biology of Phanerotomini, see van Achterberg (1990), Yu et al. (2016), and van Achterberg et al. (2017).

It is likely that still several species remain to be discovered considering the small part of the peninsula examined and the amount of unique specimens among the large (> 5,500) collection. Specimens are often difficult to identify because of the many very similar species and the lack of comprehensive and well-illustrated modern revisions. It is important that the taxonomy of the Phanerotomini is properly understood because it includes parasitoids of several species which may be pests in orchards, e.g., of citrus, lychee and coffee. Two European species (*P. fracta* and *P. flavitestacea*) have been introduced into the U.S.A. for biological control purpose (the first under the incorrect name of *P. planifrons*).

The extremely variable colour of several species in the Phanerotomini is a problem in their identification as in many other groups of Braconidae. Also, the variable shape of the third metasomal tergite (including presence or absence of an apical emargination) of some species, the pronounced sexual dimorphism, and the variation in sculpture may cause problems in the species recognition. Large and preferably reared series are essential to recognize the species limits. The association of males with females is provisional in most cases, especially when there are no reared series available.

## Materials and methods

The specimens are deposited in the collection of Naturalis Biodiversity Center, Leiden (**RMNH**) and in the United Arab Emirates Insect Collection (**UAEIC**). If no collector is mentioned, the specimens have been collected by A. van Harten. Important to note is the correct view on the more or less compressed apical half of the female antenna; the view at the maximum width is used for measurements and illustrations.

For the terminology used in this paper, see van Achterberg (1988, 1993), for identification of the subfamily Cheloninae, see van Achterberg (1993), for the genera *Phanerotoma* and *Phanerotomella*, see van Achterberg (1990), and for the existing literature, see Yu et al. (2016). An asterisk indicates if a species is newly recorded for a country. Some of the measurements have numbers in parenthesis which means that this concerns one or more exceptional specimen(s). For example, length of fore wing (1.5–)1.9–2.9 mm means that nearly all specimens have the fore wing 1.9–2.9 mm, but exceptionally specimens occur with fore wing shorter than 1.9 mm to as low as 1.5 mm. The number in parenthesis after a locality concerns the collection number given by A. van Harten.

**Abbreviations used**:

**MG** Museum of Natural History, Genève;

**MMB**Moravian Museum, Brno;

**NHMUK**Natural History Museum, London;

**NMW**Naturhistorisches Museum Wien;

**RMNH**Naturalis Biodiversity Center, Leiden;

**UAE** United Arab Emirates.

## Results

### Braconidae Nees, 1811

**Cheloninae Foerster, 1863**

**Phanerotomini Baker, 1926**

### Key to Arabian species of the tribe Phanerotomini Baker

**Table:** 

1	Vein 2-R1 of fore wing distinctly developed (Fig. 391); vein CU1b of fore wing absent, resulting in an open first subdiscal cell apico-posteriorly (Fig. 391); antennal segments 33–35; vein r of hind wing absent; vein M+CU of hind wing shorter than vein 1-M (Fig. 391); [***Phanerotomella*** Szépligeti, 1900]	***P. yemenitica* sp. nov.**
–	Vein 2-R1 of fore wing absent or as a short stub (Figs 58, 71, 156, 210); short vein CU1b of fore wing usually present, resulting in a closed first subdiscal cell apico-posteriorly (Figs 40, 58, 71, 210); antennal segments (of both sexes) 23, rarely up to 27; complete vein r of hind wing usually present (Figs 58, 210); vein M+CU of hind wing equal to vein 1-M or longer (Figs 40, 348); [***Phanerotoma*** Wesmael, 1838]	**2**
2	First discal cell of fore wing as high as first subdiscal cell (Fig. 363); vein 1-R1 of fore wing ca. ½ as long as distance between apex of vein 1-R1 and apex of wing (Fig. 363); third metasomal tergite shiny, dark brown (but sometimes brown), mostly smooth and flat apically (Figs 365, 366); malar space long in lateral view (Fig. 371); mesoscutum (Fig. 364) and vertex (Fig. 369) coarsely rugose; [parastigma large; third tergite 1.8–2.2 × longer than second tergite]	***P. stenochora* sp. nov.**
–	First discal cell of fore wing much higher than first subdiscal cell (Figs 253, 325, 348); vein 1-R1 of fore wing at least approx. as long as distance between apex of vein 1-R1 and apex of wing (Figs 253, 348, 377); third tergite usually with satin sheen or matt, often brownish yellow and at least partly superficially sculptured and often convex apically (Figs 255, 256, 350, 351, 379, 380); malar space short to medium-sized in lateral view (Figs 232, 333, 356, 385); mesoscutum and vertex finely rugose or rugulose	**3**
3	Third tergite of ♀ 1.8–2.1 × as long as second tergite and laterally straight or weakly curved, with metasomal sutures comparatively wide (Figs 60, 101, 212); antenna of ♀ at most with five moniliform apical segments (Figs 67, 103, 220); [if vein 1-R1 ca. ½ as long as pterostigma, cf. *P. cyrenaica* Masi, 1932, from N. Africa]	**4**
–	Third tergite of ♀ 1.0–1.9 × as long as second tergite (Figs 143, 184, 201); if 1.7–1.9 × (Fig. 158), then third tergite curved laterally and metasomal sutures often narrower (Fig. 158) or antenna of ♀ widened and with ca. ten moniliform apical segments (Fig. 166)	**6**
4	Vein 3-SR of fore wing approx. as long as vein r (Fig. 210); vein r of fore wing dark brown; ovipositor sheath moderately wide and apical half dark brown (Fig. 213); inner tooth of mandible large, 0.8 × as long as apical tooth (Fig. 215); clypeus less transverse (Fig. 218); length of eye ca. 3.4 × temple in dorsal view (Fig. 217) and ca. 2.3 × wider in lateral view (Fig. 219); apical half of pterostigma dark brown	***P. longivena* sp. nov.**
–	Vein 3-SR of fore wing 1.5–5.0 × as long as vein r (Figs 58, 99), if 1.5–2.5 × then vein r of fore wing yellow; ovipositor sheath narrow, needle-shaped and apical half yellow (Figs 61, 102; inner tooth of mandible small, 0.1–0.3 × as long as apical tooth (Figs 68, 104); clypeus more transverse (Figs 64, 106); length of eye 1.7–2.5 × in dorsal view (Figs 63, 105) and 1.6–1.9 × wider in lateral view (Figs 65, 107); apical half of pterostigma yellow, at most somewhat darkened	**5**
5	Temple mostly smooth, posteriorly finely aciculate, anteriorly at most punctulate and very shiny (Fig. 107); vein r of fore wing yellow and 0.4–0.7 × as long as vein 3-SR (Fig. 99); vein SR1 of fore wing nearly straight (Fig. 99); apical triangular appendage of hypopygium of ♀ short (Fig. 102)	***P. flavivena* Edmardash & Gadallah, 2019**
–	Temple rugulose or striate, matt to slightly shiny (Fig. 65); vein r dark brown and ca. 0.2 × as long as vein 3-SR (Fig. 58); vein SR1 of fore wing distinctly curved (Fig. 58); apical triangular appendage of hypopygium of ♀ medium-sized (Fig. 61); [lamella of third tergite more or less protruding latero-apically (Fig. 62)]	***P. brunneivena* sp. nov.**
6	Ventral half of temple very shiny, mostly smooth, at most punctulate (Fig. 121) and subapical antennal segments without minute subapical protuberances (Fig. 117); vein 2-SR of fore wing distinctly curved (Fig. 112); median carina of frons absent (Fig. 119); face and frons medially at least partly smooth and very shiny (Fig. 120); apical antennal segments of ♀ non-moniliform and segments cylindrical (Fig. 117); [vein SR1 of fore wing straight; vein 1-R1 of fore wing 3.0–3.6 × distance between apex of marginal cell and apex of wing (Fig. 112); temple distinctly convex (Fig. 119)]	***P. glabritemporalis* sp. nov.**
–	Ventral half of temple matt to slightly shiny, granulate, rugulose or striate (Figs 65, 135, 149, 176, 206); if very shiny (Fig. 273) and more or less finely aciculate then subapical antennal segments somewhat serrate, because of minute subapical protuberances (Fig. 275), vein 2-SR of fore wing straight (Fig. 265) and median carina of frons present (Fig. 272); face nearly entirely densely sculptured and with satin sheen or rather shiny (Figs 24, 64, 300); frons often rugose (Figs 24, 63); apical antennal segments of ♀ either moniliform (Figs 249, 304, 335, 346, 382) or somewhat widened subapically (Figs 27, 358)	**7**
7	Fore tarsus long setose, several setae approx. as long as twice width of tarsal segments (Fig. 319); maximum width of head 0.8–0.9 × maximum width of mesoscutum (Fig. 317); length of fore wing 5.4–5.5 mm; [length of body ca. 7 mm; malar space 0.7 × basal width of mandible]	***P. robusta* Zettel, 1988**
–	Fore tarsus normally setose, setae at most as long as width of tarsal segments (Fig. 320); head wider than maximum width of mesoscutum (Fig. 318); length of fore wing at most 4.5 mm	**8**
8	Marginal cell of fore wing of ♀ small, distance between wing apex and apex of marginal cell 0.6–1.1 × vein 1-R1 (Figs 141, 224, 253); vein 1-R1 of fore wing 0.7–0.9 × as long as pterostigma (Figs 141, 224, 253); temple mainly granulate (Figs 149, 232); ocelli small (Figs 147, 230, 259); vein 3-SR of fore wing usually shorter and at most somewhat longer than vein r (Figs 141, 224, 253); [parastigma medium-sized (Fig. 224)]	**9**
–	Marginal cell of fore wing of ♀ medium-sized to large, distance between wing apex and apex of marginal cell 0.1–0.5 × vein 1-R1 ((Figs 156, 168, 199, 239, 265; up to 0.7 × in ♂); vein 1-R1 of fore wing 0.9–1.4 × as long as pterostigma (Figs 156, 199); if intermediate then ocelli medium-sized or large (Figs 133, 188) and temple rugulose or finely striate (Fig. 135) or vein 3-SR of fore wing much longer than vein r (Fig. 182)	**11**
9	Second tooth of mandible small (0.3–0.4 × as long as apical tooth; Fig. 258); hind tibia with distinct dark brown subbasal patch (Fig. 257), rarely only brownish; four or five apical segments of ♀ antenna moniliform (Fig. 262); [scapus darker than third antennal segment (Fig. 263)]	***P. microdonta* sp. nov.**
–	Second tooth of mandible medium-sized to large (0.6–0.8 × as long as apical tooth; Figs 151, 229); hind tibia usually with faint brownish subbasal patch (Figs 145, 233); antenna of ♀ usually with > five moniliform apical segments (Figs 150, 234)	**10**
10	Third metasomal tergite acute apically in lateral view and without transverse depression (Fig. 227), partly smooth and shiny medially (Fig. 226); area of mesosternum near mesosternal sulcus shiny and superficially sculptured or smooth; ocelli larger (Fig. 230); [antenna of ♀ with at least seven moniliform apical segments (Fig. 234)]	***P. masiana* Fahringer, 1934**
–	Third tergite more or less obtuse apically in lateral view (Fig. 144) or with transverse depression, finely sculptured and matt medially (Fig. 143); area of mesosternum near mesosternal sulcus rather matt and distinctly granulate; ocelli smaller (Fig. 147)	***P. granulata* sp. nov.**
11	Hypopygium of ♀ straight apically in lateral view (Figs 19, 88, 171, 268, 351), truncate in ventral view, without apical triangle or spine-like protuberance; apical half of antenna of ♀ more or less serrate in lateral view because of small subapical protuberances on segments, sixth segment subapically narrowed (Figs 27, 95, 275, 358, but hardly so in *P. latifemorata*: Fig. 178); length of vein r of fore wing variable, if short compared to vein 3-SR (Fig. 85), then vein cu-a of fore wing much longer than vein 1-CU1 (Fig. 85) or first discal cell of fore wing very wide anteriorly (Fig. 168) and middle tibia narrow (Fig. 173)	**12**
–	Hypopygium of ♀ protruding apically in ventral view, spine-like (Figs 32, 47, 74) or with up curved apical triangle in lateral view (Figs 5, 130, 185, 283, 297); apical half of antenna of ♀ normal in lateral view, sixth segment from apex cylindrical and truncate apically (Figs 12, 50, 81, 132); if sixth segment from apex more or less narrowed subapically (Figs 166, 290) and vein r of fore wing short compared to vein 3-SR (Figs 156, 199, 280) then vein cu-a at most somewhat longer than 1-CU1 or shorter (Figs 156, 199, 280), or first discal cell narrower anteriorly (Fig. 199) or middle tibia rather wide (Figs 198, 279); [scutellar sulcus narrow]	**17**
12	Vein cu-a of fore wing 1.7–2.2 × as long as vein 1-CU1 (Fig. 85); intertentorial distance of clypeus 4.0–5.0 × minimum distance between clypeus and eye, clypeus approx. as wide as face and very shiny (Fig. 91); [hind femur and tibia of ♀ and tarsal claws rather slender (Fig. 82); vein r of fore wing 0.5–1.0 × vein 3-SR and forming an angle (Fig. 85)]	***P. ejuncida* sp. nov.**
–	Vein cu-a of fore wing approx. as long as vein 1-CU1 or slightly longer (Figs 168, 265, 377); intertentorial distance of clypeus 1.2–3.3 × minimum distance between clypeus and eye, width of clypeus 0.7–0.9 × minimum width of face and less shiny (Figs 175, 272, 383)	**13**
13	Temple narrow in lateral view (width of eye 2.0–2.5 × median width of temple; Figs 273, 385) and directly narrowed behind eyes in dorsal view (Figs 271, 384); face dorsally or frons anteriorly with short median carina (Figs 272, 383); [width of clypeus 0.9 × minimum width of face (Figs 272, 383)]	**14**
–	Temple wider in lateral view (width of eye 1.3–1.6 × median width of temple; Figs 25, 176, 356) and gradually narrowed behind eyes in dorsal view (Figs 174, 354, but directly narrowed in *P. artocornuta*: Fig. 23); face dorsally or frons anteriorly without median carina (Figs 24, 175, 355)	**15**
14	Apical antennal segments of ♀ somewhat serrate, because of minute subapical protuberances and elongate (Fig. 275); hind tibia slender medially (Fig. 269); face shiny and less sculptured (Fig. 272); ventral half of temple shiny and more or less finely aciculate (Fig. 273); clypeus more transverse (Fig. 272); hind tibia subbasally brownish (Fig. 269)	***P. micrommata* sp. nov.**
–	Apical antennal segments of ♀ distinctly moniliform and rather short (Fig. 382); hind tibia widened medially (Fig. 381); face usually densely sculptured and rather matt (Fig. 383); temple rather matt and densely striate (Fig. 385); clypeus less transverse (Fig. 383); hind tibia subbasally usually partly dark brown (Fig. 381)	***P. vanharteni* sp. nov.**
15	Vein r of fore wing 0.2 × as long as vein 3-SR and distinctly angled with vein 3-SR (Fig. 168); vein 2-SR of fore wing distinctly bent (Fig. 168); clypeus 0.9 × minimum width of face and very shiny (Fig. 175); [hind femur and tibia of ♀ and tarsal claws robust (Figs 167, 172)]	***P. latifemorata* sp. nov.**
–	Vein r of fore wing 0.6–1.7 × as long as vein 3-SR and less angled with vein 3-SR (Figs 16, 348); vein 2-SR of fore wing straight or weakly curved (Figs 16, 348); width of clypeus 0.7–0.8 × minimum width of face and less shiny (Figs 24, 355)	**16**
16	Clypeus semi-circular and distinctly protruding medio-ventrally (Fig. 355); parastigma at least partly brown and medium-sized (Fig. 348); vein 2-SR of fore wing straight (Fig. 348); part of pterostigma and vein r brown (Fig. 348); hind tibia of ♀ slenderer (Fig. 352); temples gradually narrowed behind eyes (Fig. 354); length of fore wing 2.8–3.8 mm	***P. spuriserrata* sp. nov.**
–	Clypeus comparatively transverse and hardly protruding medio-ventrally (Fig. 24); parastigma yellow and larger (Fig. 16); vein 2-SR of fore wing weakly curved (Fig. 16); pterostigma and vein r pale yellow (Fig. 16); hind tibia of ♀ rather swollen (Fig. 20); temples directly narrowed behind eyes (Fig. 23); length of fore wing 1.8–2.4 mm; [if vein r of fore wing short and subapical antennal segments with erect subapical bristle, cf. males of *P. lepta*]	***P. artocornuta* sp. nov.**
17	Vein r of fore wing 0.7–1.5 × as long as vein 3-SR (Figs 29, 127); if 0.7–0.9 × (Figs 29, 294) then either second tooth of mandible large compared to apical tooth (Fig. 302) or mesoscutum very finely sculptured (Fig. 30) and third metasomal tergite broadly truncate posteriorly in dorsal view (Fig. 31); [vein 1-M of fore wing pale yellowish or brown; head directly narrowed behind eyes (Figs 35, 133), but more gradually in *P. permixtellae*: Fig. 299)]	**18**
–	Vein r of fore wing 0.2–0.9 × as long as vein 3-SR (Figs 44, 71, 156, 182, 239, 280); if 0.7–0.9 × then second tooth of mandible much smaller than apical tooth (Fig. 206) and mesoscutum somewhat coarser sculptured (Fig. 200); third tergite rounded posteriorly or emarginate in dorsal view (Figs 46, 73, 158, 184, 201, 241, 282)	**20**
18	Vein r of fore wing slightly reclivous (in relation to pterostigma; Fig. 127); third metasomal tergite rather shiny, often superficially sculptured or smooth (Fig. 129); [inner tooth of mandible ca. 0.8 × apical tooth (Fig. 137); third tergite flat medially and distinctly acute posteriorly in lateral view (Fig. 130)]	***P. graciloides* van Achterberg, 1990**
–	Vein r of fore wing vertical (Fig. 294); third tergite rather matt and densely sculptured (Fig. 296)	**19**
19	Third metasomal tergite slightly convex medially (Fig. 297), rounded posteriorly in dorsal view (Fig. 296) and obtuse posteriorly in lateral view (Fig. 297); hypopygium of ♀ with short triangular protuberance (Fig. 297); apical antennal segments of ♀ stout (Fig. 304); inner tooth of mandible ca. 0.9 × apical tooth (Fig. 302)	***P. permixtellae* Fischer, 1968**
–	Third tergite flat medially (Fig. 32), broadly truncate posteriorly in dorsal view (Fig. 31) and acute posteriorly in lateral view (Fig. 32); hypopygium of ♀ with narrow and long spine-like protuberance apically (Fig. 32); apical antennal segments of ♀ slenderer (Fig. 39); inner tooth of mandible ca. 0.5 × apical tooth (Fig. 34)	***P. aspidiota* sp. nov.**
20	Hypopygium of ♀ with long spine-like, acute triangular protuberance (Figs 47, 74); apical half of hind tibia with large dark brown patch laterally and yellowish or brownish ventrally (Figs 54, 75); inner tooth of mandible 0.4–0.5 × apical tooth (Figs 49, 77); third tergite at least partly dark brown medially (Figs 46, 73); [medially third tergite 1.0–1.2 × longer than second tergite and excavated posteriorly (Figs 46, 73)]	**21**
–	Hypopygium of ♀ with short acute triangular protuberance (Figs 159, 202, 242, 283, 340); apical half of hind tibia yellowish brown or largely dark brown laterally, ventrally more or less similarly coloured (Figs 160, 186, 203, 243, 284); inner tooth of mandible often 0.1–0.2 × apical tooth of mandible (Figs 193, 206, 244, 285, 345); third tergite pale yellowish, brownish yellow or brown medially (Figs 184, 201, 282)	**22**
21	Ocelli medium-sized (POL shorter than diameter of posterior ocellus; Fig. 78); third tergite posteriorly yellowish brown, widely excavated and laterally nearly straight (Fig. 73); pale basal part of pterostigma medium-sized and contrasting with dark brown middle of pterostigma (Fig. 71); head distinctly excavated posteriorly (Fig. 78); whitish blister of middle tibia medium-sized and distinctly protruding out of its dark brown surroundings (Fig. 76)	***P. caudatoides* sp. nov.**
–	Ocelli small (POL equal to diameter of posterior ocellus or longer; Fig. 51); third tergite posteriorly dark brown, moderately excavated and laterally curved (Fig. 46); pale basal part of pterostigma small and less contrasting with dark brown middle (Fig. 44); head less excavated posteriorly (Fig. 51); whitish blister of middle tibia minute and less contrasting with its surroundings (Fig. 42)	***P. bilinea* Lyle, 1924**
22	Distance between apex of marginal cell and apex of fore wing 0.3–0.5 × vein 1-R1 (Figs 2, 182, 337); vertex finely rugulose (Figs 10, 190, 345); eye in lateral view 1.3–2.1 × as wide as temple measured medially (Figs 10, 190, 345); ocelli often smaller (POL 0.8–1.3 × width of posterior ocellus; Figs 8, 188, 343); third tergite of metasoma flattened in lateral view (Figs 185, 340, but less so in *P. angusticrus*: Fig. 5); second submarginal cell of fore wing smaller (Figs 2, 182, 337); anterior half of vein 1-M yellow (Figs 182, 337, but less so in *P. angusticrus*: Fig. 2); head less emarginate medio-posteriorly (Figs 8, 188, 343); [inner tooth of mandible small or minute: (Figs 193, 345), but larger in *P. angusticrus*: Fig. 7)]	**23**
–	Distance between apex of marginal cell and apex of fore wing 0.1–0.3 × vein 1-R1 (Figs 156, 199, 239); if 0.3 × then vertex distinctly rugose or temple in lateral view comparatively wide (Figs 206, 247); ocelli often larger (POL 0.5–1.0 × width of posterior ocellus (Figs 162, 245); third tergite of metasoma convex in lateral view (Figs 159, 202, 242); anterior half of vein 1-M more or less infuscate (Figs 156, 199, 239); second submarginal cell of fore wing medium-sized (Figs 156, 199, 239); head usually distinctly emarginate medio-posteriorly (Figs 204, 245, but hardly emarginate in *P. hellyeri*: Fig. 162); [hind tibia stout; vein 2-SR more or less curved or bent (Fig. 156, 199, 239), angle between veins 2-SR and 3-SR > 90°; scutellar sulcus rather wide (Figs 157, 200, 240); inner tooth of mandible 0.1–0.2 × apical tooth (Figs 206, 244)]	**25**
23	Eye in lateral view approx. twice as wide as temple measured medially (Fig. 10); hind femur and tibia of ♀ slender (Fig. 6); vein SR1 of fore wing sinuate or curved (Fig. 2); blister of middle tibia hardly developed (Fig. 1); clypeus comparatively large (Fig. 9)	***P. angusticrus* sp. nov.**
–	Eye in lateral view 1.3–1.4 × as wide as temple measured medially (Figs 190, 345); hind femur and tibia of ♀ wider (Figs 186, 341); vein SR1 of fore wing straight (Figs 182, 337); blister of middle tibia small to medium-sized; clypeus smaller (Figs 189, 344)	**24**
24	Second submarginal cell of fore wing small (Fig. 337); vein r of fore wing ca. 0.7 × vein 3-SR (Fig. 337); head 1.4 × wider than high medially in anterior view (Fig. 344); pterostigma conspicuously dark and large compared to weakly pigmented venation (Fig. 337); POL 1.2–1.3 × width of posterior ocellus (Fig. 343); head distinctly emarginate posteriorly in dorsal view (Fig. 343)	***P. signifera* sp. nov.**
–	Second submarginal cell of fore wing larger (Fig. 182); vein r of fore wing 0.3–0.4 × vein 3-SR (Fig. 182); head 1.5 × wider than high medially in anterior view (Fig. 189); pterostigma and venation normally pigmented (Fig. 182); POL 0.8–1.0 × width of posterior ocellus (Fig. 188); head hardly emarginate posteriorly in dorsal view (Fig. 188)	***P. lepta* sp. nov.**
25	Seventh-ninth antennal segments from apex of ♀ stocky, matt or slightly shiny (Figs 166, 249); antenna of ♀ with eight–thirteen short and more or less moniliform segments (Figs 166, 249); vein 1-M (as usually parastigma) slightly darker than yellow M+CU1 of fore wing (Figs 156, 239); eye in lateral view 1.4–2.1 × as wide as medial width of temple because of large eyes (Figs 164, 247); if 1.4 × then mesosternum more or less shiny; ovipositor sheath narrow apically (Figs 159, 242)	**26**
–	Seventh–ninth antennal segments from apex of ♀ less stocky and moderately to distinctly shiny (Figs 208, 290, 335); antenna of ♀ with five–seven moniliform segments and basal segments longer (Figs 208, 290, 335); vein 1-M usually distinctly darker (as usually most of parastigma) than yellow M+CU1 of fore wing (Figs 199, 280, 325); eye in lateral view 1.0–1.6 × as wide as maximum width of temple because of medium-sized eyes (Figs 206, 333); if 1.3–1.6 × then mesosternum with satin sheen or matt and finely granulate; ovipositor sheath slightly wider apically (Figs 202, 283), but intermediate in *P. sculptilis*: Fig. 328)	**27**
26	Eye 2.0–2.2 × as wide as median width of temple in lateral view (Fig. 164); stemmaticum brownish yellow (Fig. 162), very rarely infuscate; eighth–tenth antennal segments of ♀ distinctly moniliform and 14^th^ segment from apex approx. as long as wide (Fig. 166); second submarginal cell of fore wing somewhat longer (Fig. 156); POL of ♀ 0.6–0.9 × diameter of posterior ocellus (Fig. 162)	***P. hellyeri* sp. nov.**
–	Eye 1.2–1.8 × as wide as median width of temple in lateral view (Fig. 247); stemmaticum black or dark brown (Fig. 245), but rarely brownish yellow; eighth–tenth antennal segments of ♀ less moniliform and 14^th^ segment from apex somewhat longer than wide (Fig. 249); second submarginal cell of fore wing shorter (Fig. 239); POL of ♀ 0.4–0.6 × width of posterior ocellus (Fig. 245)	***P. mesocellata* sp. nov.**
27	Ocelli moderately large, POL of ♀ 0.4–0.5 × width of posterior ocellus (Fig. 286); antenna of ♀ more abruptly narrowed apically (Fig. 289); temple narrower in lateral view (Fig. 288); blister of middle tibia more differentiated and tibia rather robust (Fig. 279); [third tergite evenly brown (Fig. 282); first discal cell wide anteriorly (Fig. 280); tenth segment from apex of antenna of ♀ 1.0–1.2 × as long as wide; hind femur of ♂ 1.1–1.2 × as wide as hind tibia (Fig. 278)]	***P. ocularis* Kohl, 1906**
–	Ocelli distinctly smaller, POL of ♀ 0.6–1.0 × width of posterior ocellus (Figs 204, 331); antenna of ♀ often more gradually narrowed apically (Figs 207, 334); temple wider in lateral view (Figs 206, 333); blister of middle tibia less differentiated and tibia slenderer (Fig. 198)	**28**
28	Mesosternum granulate and matt; third metasomal tergite in lateral view distinctly convex and apically obtuse (Fig. 202); hind tibia wider medially (Fig. 203); clypeus moderately shiny (Fig. 205); first discal cell of fore wing narrower anteriorly (Fig. 199); apical antennal segments of ♀ non-moniliform (Fig. 208); [third tergite sometimes laterally darker than medially (Fig. 201); hind tibia more or less maculate (Fig. 203)]	***P. leucobasis* Kriechbaumer, 1894**
–	Mesosternum punctulate and shiny; third metasomal tergite in lateral view flattened and apically acute (Fig. 328); hind tibia narrowed medially (Fig. 329); clypeus very shiny (Fig. 332); first discal cell of fore wing wider anteriorly (Fig. 325); apical antennal segments of ♀ moniliform (Fig. 335)	***P. sculptilis* sp. nov.**

### Descriptions

#### Phanerotoma

Taxon classificationAnimaliaHymenopteraBraconidae

Wesmael, 1838

32337F30-D2BC-55C3-8957-D37D6C7F5F45

Figs 1 , 2–12 , 13–15 , 16–27 , 28 , 29–39 , 40–43 , 44–54 , 55–57 , 58–68 , 69, 70 , 71–81 , 82–84 , 85–95 , 96–98 , 99–108 , 109–111 , 112–123 , 124–126 , 127–137 , 138–140 , 141–151 , 152–155 , 156–166 , 167 , 168–178 , 179–181 , 182–193 , 194–198 , 199–208 , 209 , 210–220 , 221–223 , 224–234 , 235–238 , 239–249 , 250–252 , 253–263 , 264 , 265–275 , 276–279 , 280–290 , 291–293 , 294–304 , 305 , 306–316 , 317–320 , 321–324 , 325–335 , 336 , 337–346 , 347 , 348–358 , 359–362 , 363–373 , 374–376 , 377–386

Phanerotoma Wesmael, 1838: 165. Type-species: Chelonus dentatus Panzer, 1805. Designated by Haliday 1840: 63; Shenefelt, 1973: 909–910.
Phanerogaster Wesmael, 1838: 165 (unavailable name, published in synonymy with Phanerotoma)
Sulydus du Buysson, 1897: 354. Type-species: Sulydus marshalli du Buysson, 1897 (examined). Monotypic.
Ichneutipterus Vachal, 1907: 122. Type-species: *Sigalphus? ichneutipterus* Vachal, 1907. Synonymized by van Achterberg (1990) (examined). Monotypic.
Neophanerotoma Szépligeti, 1908b: 227. Type-species: Phanerotoma orientalis Szépligeti, 1902 (examined). Designation by Viereck 1914: 99.
Tritoma Szépligeti, 1908a: 410 (not Fabricius, 1775). Type-species: Chelonus tritomus Marshall, 1898. Synonymized by van Achterberg (1990) (examined). Monotypic.
Bracotritoma Csiki, 1909: 13. Synonymized by van Achterberg (1990). Replacement name for Tritoma Szépligeti.
*Szépligetia* Schulz, 1911: 89. Synonymized by van Achterberg (1990). Replacement name for Tritoma Szépligeti.
Neoacampis Szépligeti, 1914: 210. Type-species: Neoacampis gracilipes Szépligeti, 1914. Synonymized by van Achterberg (1990) (examined). Monotypic.
Tritomios Strand, 1921: 174. Synonymized by van Achterberg (1990). Replacement name for Tritoma Szépligeti.
Phanerotomina Shestakov, 1930: 100. Type-species: Phanerotomina gussakovskii Shestakov, 1930 (= Phanerotoma parva Kokujev, 1903). Monotypic.
Unica Šnoflák, 1951: 7, 9. Type-species: Phanerotoma moravica Snoflák, 1951 (examined). Monotypic.

#### Phanerotoma angusticrus sp. nov.

Taxon classificationAnimaliaHymenopteraBraconidae

2F96C1A3-DD1C-5D09-9288-7F3B4C0591CB

http://zoobank.org/3D0C5AB1-1302-4149-A2AE-1095D3B21919

Figs 1 , 2–12

##### Type material.

***Holotype***, ♀ (RMNH), “**United Arab Emirates**, al-Ajban (6418), Malaise & light tr[ap], 7–28.xii.2006, 24°36'N, 55°01'E, A. v. Harten, RMNH’07”. ***Paratypes***: 1♀: “**Yemen**: Al Kowd (8136), ix.2003, light trap, A. v. Harten & S. Al Haruri, RMNH’03”; 1♀: Idem, v.–vi.2000; 1♀, “Yemen: Al Kadan (6699), iv.2002, light trap; A. v. Harten & T. Abdul-Haq, RMNH’03”; 1 ♀: “Yemen (5404), near Hamman’Ali, from coffee-berries (with *Ceratitis capitata*?), 14.viii.2001, A. v. Harten, RMNH’02”.

**Figure 1.**
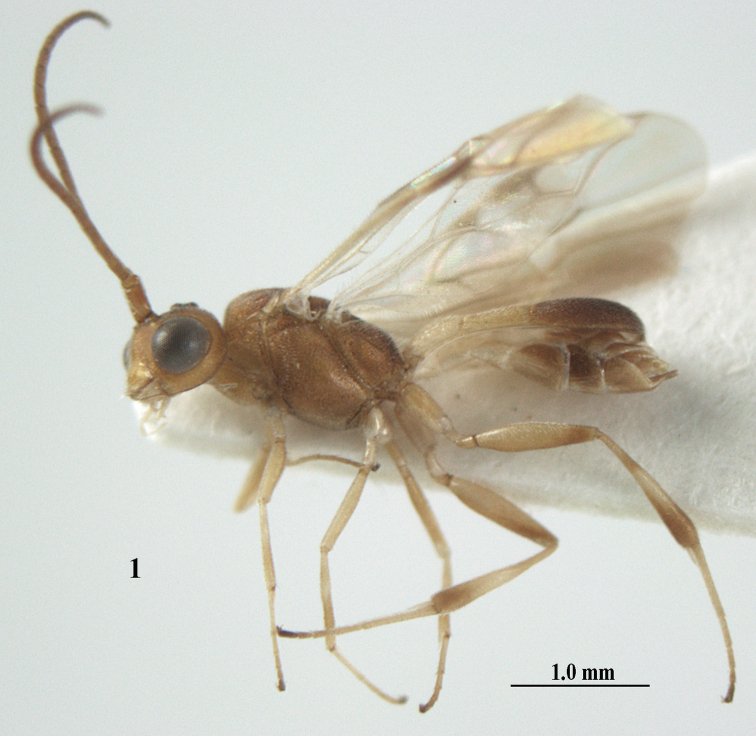
*Phanerotoma angusticrus* van Achterberg sp. nov., ♀, holotype, habitus lateral.

##### Diagnosis.

**Figures 2–12.**
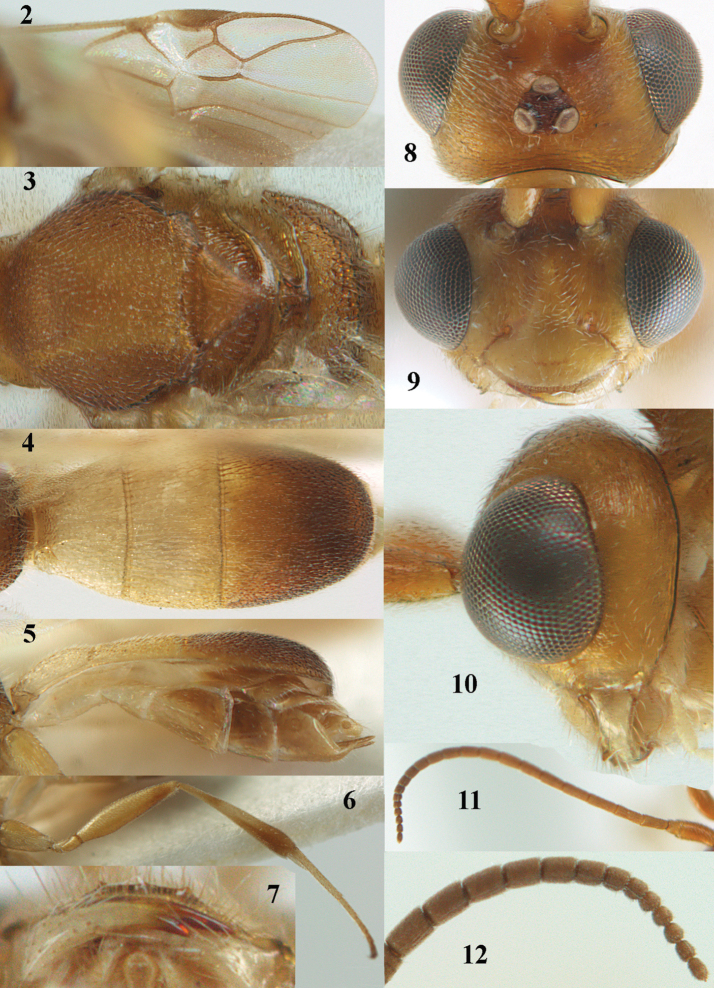
*Phanerotoma angusticrus* van Achterberg, sp. nov., ♀, holotype **2** wings **3** mesosoma dorsal **4** first–third metasomal tergites dorsal **5** metasoma lateral **6** hind leg lateral **7** mandible ventral **8** head dorsal **9** head anterior **10** head lateral **11** antenna lateral **12** apical third of antenna lateral.

Apical half of antenna of ♀ cylindrical in lateral view, not widened and subapical segments short, six segments moniliform or submoniliform (Fig. 12); vertex with satin sheen; hind femur and tibia of ♀ slender, hind femur 4.4–4.5 × longer than wide; temple in lateral view hardly widened dorsally and with satin sheen (Fig. 10); frons without median carina; face nearly entirely densely sculptured and shiny; head hardly emarginate posteriorly (Fig. 8); vein 2-SR of fore wing nearly straight (Fig. 2); vein cu-a of fore wing 1.0–1.3 × vein 1-CU1; Fig. 2); blister of middle tibia hardly developed; hypopygium of ♀ usually dark brown (Fig. 5); third tergite 1.7–1.9 × as long as second tergite, curved laterally, densely sculptured, rather dull and convex. Easily confused with *P. leucobasis*, but differs because of the narrow hind femur and tibia (Fig. 6), the less emarginate head, the flattened and longer third metasomal tergite, the somewhat wider clypeus and the less sculptured temple.

##### Description.

Female, holotype, length of body (excluding ovipositor and hypopygium) 3.8 mm; antenna 2.9 mm; fore wing 3.0 mm; visible and setose part of ovipositor sheath 0.2 mm.

***Head*.** Width 1.5 × median length in anterior view, hardly emarginate posteriorly in dorsal view and part of head above eye in lateral view 0.3 × height of eye (Figs 8, 10); antenna with 23 segments, with apical spine and approx. as long as fore wing, segments slender and gradually shortened, segments of apical half without minute subapical protuberances and cylindrical, six apical segments moniliform and narrowed basally (Figs 11, 12), third, fourth and penultimate segments 2.8, 2.6 and 1.3 × longer than wide in lateral view, respectively; area of stemmaticum superficially rugulose; OOL: diameter of posterior ocellus: POL = 16: 6: 4; length of eye 2.4 × temple in dorsal view (Fig. 8); frons micro-sculptured and shiny anteriorly, without distinct median carina and rugose laterally (Fig. 8); vertex finely transversely rugose and with satin sheen; temple granulate near eye and with fine longitudinal rugulae posteriorly, rather convex and with satin sheen; clypeus 0.9 × as wide as minimum width of face (intertentorial distance 3.8 × minimum distance between clypeus and eye ventrally), rather flat ventrally, with long erect setae, smooth and shiny (Fig. 9); face rather shiny and distinctly transversely rugulose, without median carina dorsally; clypeus with three obsolescent teeth medio-ventrally (Fig. 7); eye large, strongly convex and in lateral view 2.1 × temple (measured medially) and hardly widened dorsally (Fig. 10), in anterior view its height 0.8 × minimum width of face; upper condyle of mandible near lower level of eyes (Fig. 9); malar space coriaceous-rugulose and 0.5 × as basal width of mandible; lower tooth of mandible small and 0.4 × as long as apical tooth (Fig. 7).

***Mesosoma*** (Figs 1, 3). Length 1.4 × its width in lateral view; side of pronotum coarsely rugose and shiny; mesosternum superficially granulate and shiny; mesoscutum largely granulate-coriaceous, but medio-posteriorly distinctly rugose and with satin sheen; scutellar sulcus wide, with nine carinae (Fig. 3); scutellum widely triangular, densely finely granulate-rugulose (nearly up to posterior margin), slightly convex and with satin sheen; metanotum with nearly complete median carina and medio-posteriorly with minute tooth; propodeum coarsely reticulate-rugose, without distinct median and transverse carinae and latero-posteriorly not tuberculate. ***Wings*.** Fore wing 2.6 × longer than its maximum width; length of 1-R1 1.1 × as long as pterostigma; r issued rather far beyond middle of pterostigma and 0.4 × 3-SR; distance between 1-R1 and wing apex 0.5 × 1-R1; 2-SR slightly curved and distally converging to posterior margin of pterostigma (Fig. 2); SR1 curved; 2-SR+M present, because of narrowly postfurcal m-cu; parastigma large; 1-CU1 0.3 × as long as vein 2-CU1, cu-a strongly inclivous and 1.2 × as long as 1-CU1; r:3-SR:SR1 = 5:13:42; 2-SR:3-SR:r-m = 18:13:7; r-m reclivous; 2-M weakly curved (Fig. 2). Hind wing: M+CU:1-M:1r-m = 22:14:10. ***Legs*.** Hind femur narrow (especially apically) and 4.4 × as long as wide (Fig. 6); hind tibia rather narrow; middle tibia with small ivory blister; inner spur of middle tibia 0.5 × its basitarsus; hind coxa mostly smooth, but partly superficially granulate and shiny; hind basitarsus and tarsal claws slender (Fig. 6).

***Metasoma*** (Figs 4, 5). Elliptical in dorsal view, twice as long as wide and 1.3 × as long as mesosoma; first and second tergites finely and densely longitudinally rugose; second suture narrow; third tergite 1.7 × longer than second tergite and laterally weakly curved, in lateral view slightly convex posteriorly, in dorsal view convex medially, densely and finely rugulose and medio-posteriorly truncate (Fig. 4), lateral lamella narrow, not protruding latero-apically and medio-apically truncate and wide; ovipositor sheath narrow and parallel-sided (Fig. 5), its visible part 0.07 × as long as fore wing and 0.12 × metasomal carapace and with some long and erect setae; hypopygium apically robust, no spine, but with short up curved and setose triangle (Fig. 5).

***Colour*.** Yellowish brown; palpi, mandible (except dark brown teeth), tegulae, mesoscutum medially, legs (but hind femur ventrally, and hind tibia apically and subbasally brownish), first and second metasomal tergites and basal half of metasoma ventrally largely pale yellow or ivory; clypeus, malar space and parastigma pale yellowish; apical third of antenna, ovipositor sheath and pterostigma (but basally pale yellowish) largely brown; stemmaticum dark brown; wing membrane subhyaline but below dark part of pterostigma and near vein CU1 slightly infuscate; vein 1-M largely pale brown; veins 1- & 2-CU1, r and 3-SR of fore wing brown.

##### Male.

Unknown.

##### Variations.

Length of fore wing 2.6–3.1 mm; third tergite 1.7–1.9 × longer than second tergite; vein cu-a of fore wing 1.0–1.3 × as long as vein 1-CU1.

##### Biology.

Unknown.

##### Distribution.

United Arab Emirates, Yemen.

##### Etymology.

The name is a combination of *angustus* (Latin for narrow) and *crus* (Latin for leg), because of the slender hind femur and tibia.

#### Phanerotoma artocornuta sp. nov.

Taxon classificationAnimaliaHymenopteraBraconidae

4CFEA1B0-85D7-5E6A-9A73-D34DEAA08C4A

http://zoobank.org/63840FF1-5325-4F9C-9990-030C7F657B05

Figs 13–15 , 16–27

##### Type material.

***Holotype***, ♀ (RMNH), “**Yemen**: Al Kowd (4054), viii.1999, light trap, A. v. Harten & S. Al Haruri, RMNH’00”. ***Paratypes***: 2♀: Idem, i.–iii.2003; 5♀: Idem, iv.2001; 2♀: Idem, vii.–ix.2001; 2♀: Idem, ix.2003; 1♀: Idem, 17–21.vii.2001; 1♀: Idem, 21–25.viii.2001; 1♀: Idem, 8–12.vii.2001; 1♀: Idem, vi.2002; 1♀: Idem, v.–vi.2000; 1♂: Idem, vii.1999; 1♀, “Yemen: Ar Rujum (5700), 9.iv.–5.vi.2001, Mal. trap, A. v. Harten, RMNH’02”; 1♀, “Yemen: Al Kadan (7501), i.2003, light trap; A. v. Harten & T. Abdul-Haq, RMNH’03”; 4♀: Idem, v.2002; 1♂: “**United Arab Emirates**, Fujairah (1224), light tr[ap], 5–24.iii.2005, 25°08'N, 56°21'E, A. v. Harten, RMNH’06”.

**Figures 13–15.**
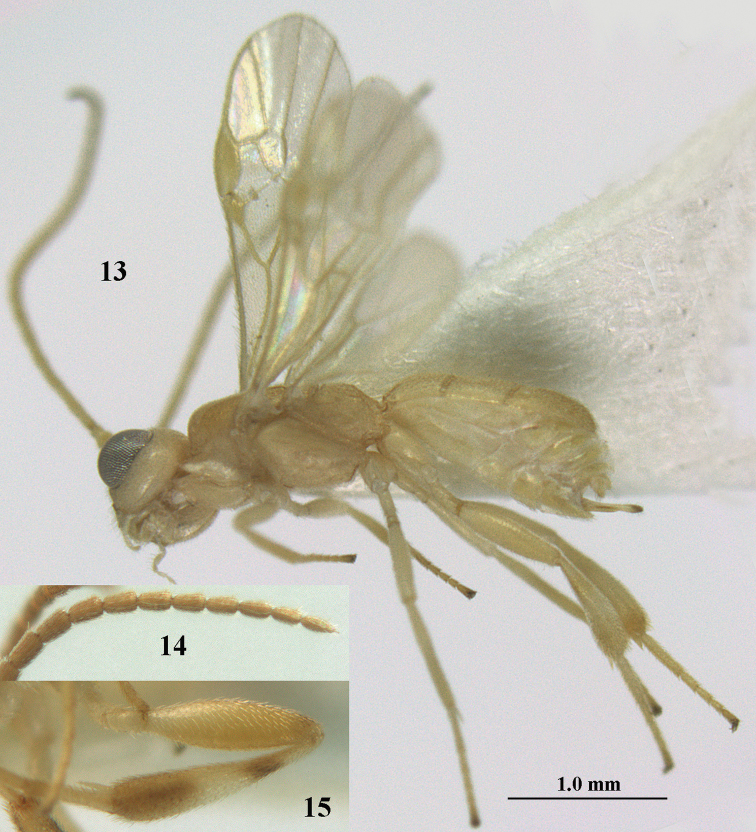
*Phanerotoma artocornuta* van Achterberg, sp. nov. ♀, holotype (but **14** and **15** of ♂, paratype) **13** habitus lateral **14** apical third of antenna lateral **15** hind femur and tibia lateral.

##### Diagnosis.

**Figures 16–27.**
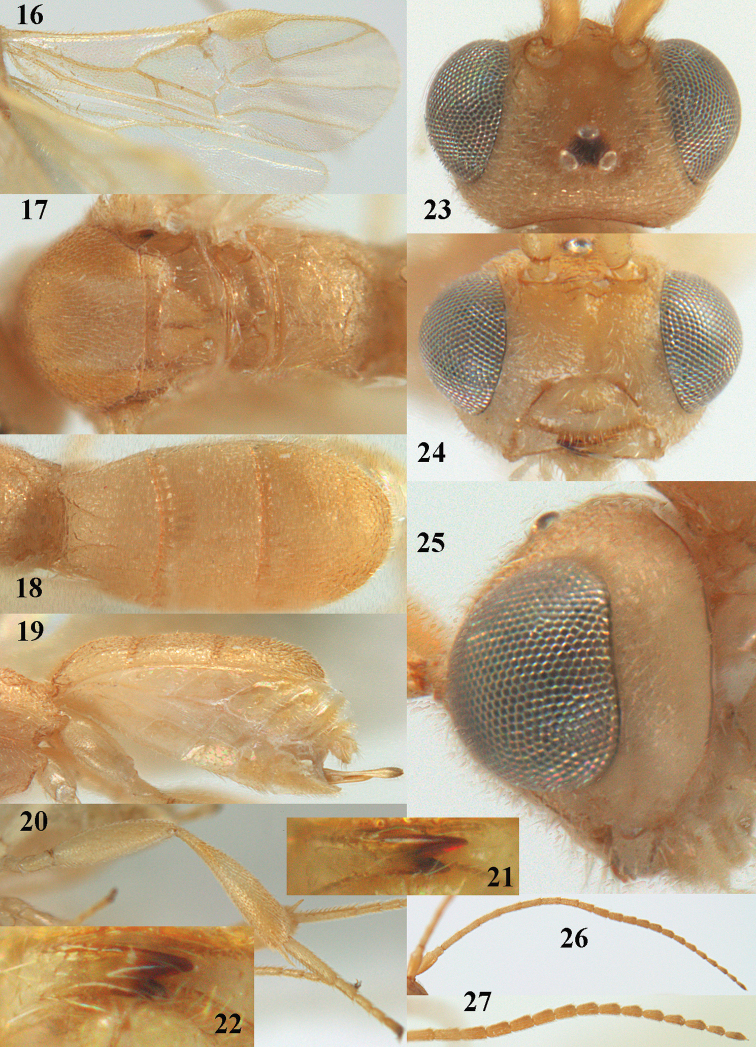
*Phanerotoma artocornuta* van Achterberg, sp. nov., ♀, holotype (but **21** of ♀ paratype) **16** wings **17** mesosoma dorsal **18** first–third metasomal tergites dorsal **19** metasoma lateral **20** hind leg lateral **21, 22** mandible ventral **23** head dorsal **24** head anterior **25** head lateral **26** antenna lateral **27** apical half of antenna lateral.

Subapical antennal segments of ♀ rather slender, sixth segment from apex narrowed basally and subapically widened and with small and round protuberances near apex, resulting in a somewhat serrate margin of antenna (Fig. 27); antenna of ♀ approx. as long as body and eight–eleventh segments from apex elongate; clypeus comparatively transverse and hardly protruding medio-ventrally (Fig. 24); parastigma yellow and comparatively large (Fig. 16); scutellar sulcus narrow to rather wide; parastigma, pterostigma and all veins pale yellow; antenna of ♀ 1.0–1.1 × as long as body; second tooth of mandible 0.4–0.5 × as long as apical tooth; maximum width of clypeus 0.8 × minimum width of face; temple densely striate and rather shiny; clypeus with 3 minute teeth; length of malar space 0.7–0.8 × basal width of mandible; vein cu-a of fore wing distinctly inclivous; vein r 0.9–1.7 × vein 3-SR and forming an angle (Fig. 16); third metasomal tergite 1.4–1.5 × as long as second tergite and with curved sides; hypopygium without up curved triangle or spine apically (Fig. 19); length of fore wing 1.8–2.4 mm. *Phanerotoma artocornuta* is similar to *P. longiradialis* because of similar antenna, inner tooth of mandible and not angled veins r and 3-SR. *P. artocornuta* differs by having apical third of antenna pseudo-serrate (cylindrical in *P. longiradialis*), anterior tentorial pits distinctly above lower level of eyes (at lower level of eyes), temples directly narrowed behind eyes (roundly narrowed), pterostigma yellow (dark brown) and vein r approx. as long as vein 3-SR (distinctly shorter).

##### Description.

Female, holotype, length of body (excluding ovipositor) 3.1 mm; antenna 2.5 mm; fore wing 2.3 mm; visible part of ovipositor sheath 0.35 mm.

***Head*.** Width 1.6 × median length in anterior view and part of head above eye in lateral view 0.35 × height of eye (Fig. 25); antenna with 23 segments and 1.1 × as long as fore wing, segments near apical quarter of antenna elongate and longer than wide, widened subapically because of small round protuberances and seven apical segments rather moniliform (Fig. 27) and apical segment with distinct spine; third, fourth, and penultimate segments 3.8, 3.4 and 2.0 × longer than wide in lateral view, respectively; area of stemmaticum coriaceous; OOL: diameter of posterior ocellus: POL = 12: 4: 5; length of eye 4.0 × temple in dorsal view (Fig. 23); frons rugose laterally and densely rugulose medially, and rather shiny, with median carina posteriorly; vertex coarsely transversely rugose-striate and with satin sheen; temple largely striate and rather shiny, its median width 0.6 × width of eye in lateral view; face finely reticulate-rugose and with median ridge dorsally, but no distinct median carina; clypeus mostly smooth, shiny, 0.8 × wider than minimum width of face (intertentorial width 2.2 × minimum distance between clypeus and eye) and with three minute teeth medio-ventrally (Fig. 24); eye large, strongly convex and in lateral view 1.3 × (measured medially) as wide as temple (Fig. 25), in anterior view its height equal to minimum width of face; upper condyle of mandible below lower level of eyes (Fig. 24); malar space rugulose, with satin sheen and 0.7 × basal width of mandible; lower tooth of mandible 0.5 × as long as apical tooth (Fig. 22).

***Mesosoma*** (Figs 13, 17). Length 1.8 × its width in lateral view; side of pronotum only medially and posteriorly rugose, remainder rugulose or superficially coriaceous; propleuron posteriorly weakly convex; mesosternum densely granulate and rather matt; mesoscutum densely rugulose; scutellum flat, densely granulate but smooth posteriorly and with satin sheen; notauli not indicated; scutellar sulcus wide and with eight carinae (Fig. 17); metanotum without short median carina anteriorly and some micro-sculpture posteriorly; propodeum coarsely rugose-reticulate, without distinct median and transverse carinae, and latero-posteriorly slightly tuberculate. ***Wings*.** Fore wing 3.0 × longer than its maximum width; 1-R1 as long as pterostigma; distance between wing apex and 1-R1 0.3 × length of vein 1-R1; r issued distinctly beyond middle of pterostigma, angled to 3-SR and 1.6 × 3-SR; 2-SR weakly curved and distally converging to posterior margin of pterostigma (Fig. 16); SR1 straight; 2-SR+M short, m-cu slightly postfurcal; parastigma rather large; first discal cell of fore wing much higher than first subdiscal cell; 1-CU1 0.45 × as long as vein 2-CU1, cu-a 0.9 × 1-CU1; r:3-SR:SR1 = 8:5:47; 2-SR:3-SR:r-m = 19:5:7; r-m nearly vertical; 2-M slightly curved (Fig. 16). Hind wing: M+CU:1-M:1r-m = 26:21:10. ***Legs*.** Hind femur with satin sheen, 3.3 × as long as wide and rather widened submedially; hind tibia rather swollen; middle tibia with medium-sized yellowish blister; inner spur of middle tibia 0.5 × its basitarsus; hind coxa superficially granulated and shiny.

***Metasoma*** (Figs 18, 19). Elliptical in dorsal view, 1.9 × as long as wide and 1.1 × as long as mesosoma; first and second tergites densely and rather coarsely longitudinally rugose; metasomal sutures medium-sized; third tergite convex medially, 1.4 × longer than second tergite and laterally curved, in lateral view rather convex, largely densely reticulate-rugulose and with satin sheen (Fig. 19), lateral lamella narrow laterally, posteriorly rather wide and not protruding latero-apically, medio-apically truncate; ovipositor sheath widened apically, its visible part 0.1 × as long as fore wing and 0.3 × metasomal carapace and its setose apical part with medium-sized setae and 0.05 × as long as fore wing; hypopygium setose and acute apically, without up curved triangle apically or apical spine (Fig. 19).

***Colour*.** Pale yellowish; apical antennal segments apically and apex of ovipositor sheath brown; stemmaticum blackish; telotarsi brownish yellow; veins (including 1-M), parastigma and pterostigma pale yellowish; wing membrane subhyaline.

##### Male.

Very similar to female, but subapical antennal segments more elongate and with an erect apical bristle (Fig. 14), hind femur somewhat widened (Fig. 15), vein 1-M, parastigma and pterostigma partly dark brown or brown.

##### Variations.

Length of fore wing 1.8–2.4 mm; inner tooth of mandible rather robust and 0.4–0.5 × as long as apical tooth; vein r of fore wing 0.5–1.7 × vein 3-SR.

##### Biology.

Unknown.

##### Distribution.

United Arab Emirates, Yemen.

##### Etymology.

From *artus* (Latin for narrow) and *cornutus* (Latin for horned) because of the slender antenna.

#### Phanerotoma aspidiota sp. nov.

Taxon classificationAnimaliaHymenopteraBraconidae

7697D7AB-88EF-50C4-9880-4735B810C628

http://zoobank.org/A3E50FFB-6935-437A-9395-7F720D0DC4FD

Figs 28 , 29–39

##### Type material.

***Holotype***, ♀ (RMNH), “**Yemen** (8136), Al Kowd, light trap, ix.2003, A. v. Harten & S. Al Haruri, RMNH’03”.

##### Diagnosis.

Third tergite flat medially, broadly truncate posteriorly in dorsal view (Fig. 31) and acute posteriorly in lateral view (Fig. 32); apical antennal segments of ♀ rather slender (Fig. 39) and 3 apical segments moniliform; head directly narrowed behind eyes (Fig. 35); inner tooth of mandible ca. 0.5 × apical tooth, stout (Fig. 34); vein r of fore wing vertical (Fig. 29); vein r of fore wing ca. 0.7 × as long as vein 3-SR; mesoscutum coriaceous-like sculptured; vein 1-R1 ca. 1.2 × as long as pterostigma; vein 1-M of fore wing brown); mesosoma approx. twice longer than high in lateral view; hypopygium of ♀ with narrow and long spine-like protuberance apically (Fig. 32).

**Figure 28.**
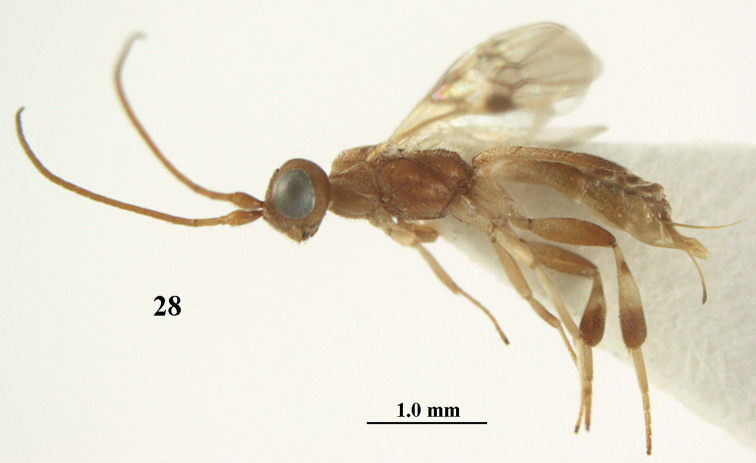
*Phanerotoma aspidiota* van Achterberg, sp. nov., ♀, holotype, habitus lateral.

**Figures 29–39.**
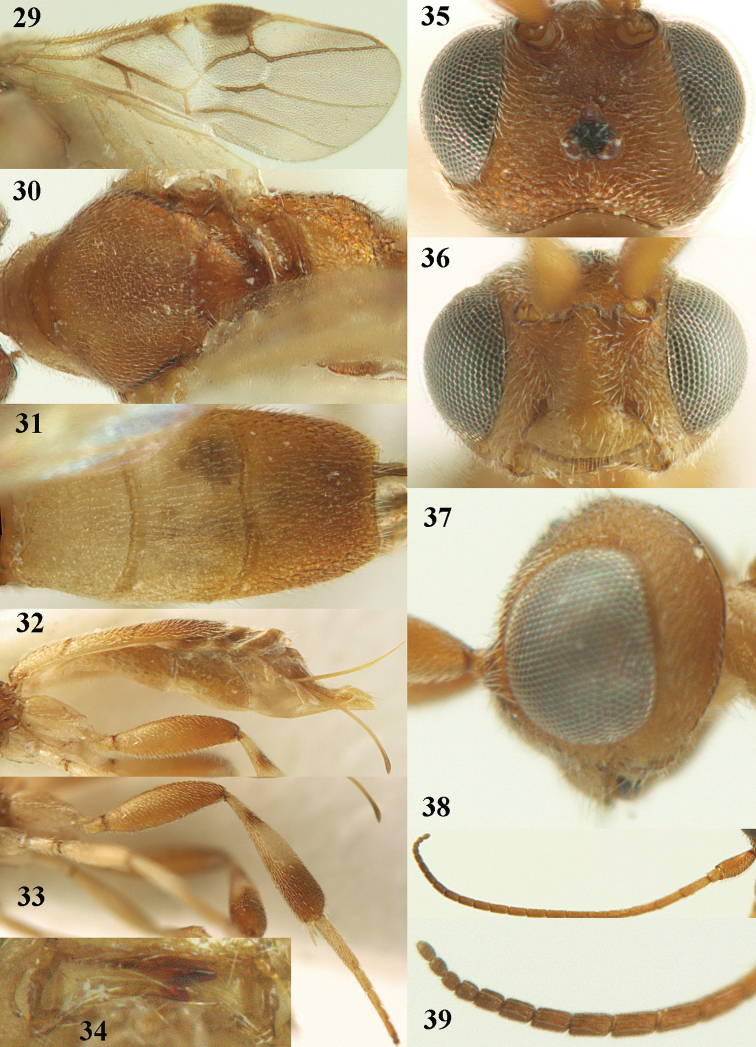
*Phanerotoma aspidiota* van Achterberg, sp. nov, ♀, holotype **29** fore wing **30** mesosoma dorsal **31** first–third metasomal tergites dorsal **32** metasoma lateral **33** hind leg lateral **34** mandible ventral **35** head dorsal **36** head anterior **37** head lateral **38** antenna, lateral **39** apical third of antenna lateral.

##### Description.

Female, holotype, length of body (excluding ovipositor) 3.4 mm; antenna 2.5 mm; fore wing 2.2 mm; visible part of ovipositor sheath 0.7 mm (0.15 mm sparsely erect setose).

***Head*.** Width 1.6 × median length in anterior view and part of head above eye in lateral view 0.2 × height of eye (Fig. 37); antenna with 23 cylindrical segments and 1.1 × longer than fore wing, three apical segments small and moniliform (Fig. 39), with short bristle apically and apical segment without spine, third, fourth and penultimate segments 3.0, 2.8 and 1.0 × longer than wide in lateral view, respectively; area of stemmaticum granulate; OOL: diameter of posterior ocellus: POL = 12: 4: 4; length of eye 3.1 × temple in dorsal view (Fig. 35); frons granulate medially, rugose laterally and without median carina; vertex rugose and with satin sheen; temple finely and densely granulate and rather dull, directly narrowed behind eyes; face transversely rugose laterally, rugulose and with obsolescent median bump and rather shiny; clypeus smooth, shiny and 0.8 × minimum width of face, intertentorial distance 2.6 × minimum width between clypeus and eye, long erect setose and with 3 indistinct blunt teeth medio-ventrally (Fig. 36); eye medium-sized, strongly convex and in lateral view 1.7 × (measured medially) wider than temple (Fig. 37), in anterior view its height equal to minimum width of face; upper condyle of mandible near lower level of eyes (Fig. 36); malar space aciculate, with satin sheen and 0.4 × as long as basal width of mandible; lower tooth of mandible half as long as apical tooth, robust (Fig. 34).

***Mesosoma*** (Figs 28, 30). Length 2.1 × its width in lateral view; side of pronotum rugose, but anteriorly punctate and dorsally granulate; propleuron posteriorly flattened; mesosternum superficially granulate and with satin sheen; mesoscutum densely and very finely rugulose, rather dull; scutellum flat, distinctly granulate (but medially partly smooth) and rather matt; scutellar sulcus narrow, with nine carinae (Fig. 30); metanotum with short median carina medially and finely serrate posteriorly; propodeum rugose, but anteriorly rugulose, without transverse carina or median carina, and latero-posteriorly weakly tuberculate. ***Wings*.** Fore wing 2.7 × longer than its maximum width; 1-R1 1.25 × as long as pterostigma; distance between wing apex and marginal cell apex 0.2 × length of 1-R1; r issued far beyond middle of pterostigma and 0.7 × 3-SR; 2-SR slightly curved and subparallel with posterior margin of pterostigma (Fig. 29); SR1 straight; 2-SR+M short; m-cu just postfurcal; parastigma medium-sized; 1-CU1 0.4 × as long as vein 2-CU1, cu-a 0.8 × 1-CU1; r:3-SR:SR1 = 5:7:34; 2-SR:3-SR:r-m = 18:7:7; r-m nearly vertical; 2-M slightly curved (Fig. 29). Hind wing: M+CU:1-M:1r-m = 16:14:5. ***Legs*.** Hind femur matt, 3.1 × as long as wide and robust; middle tibia with ivory blister; inner spur of middle tibia 0.4 × its basitarsus; hind tibia wide medially (Fig. 33); hind coxa largely coriaceous but dorsally mostly smooth and shiny.

***Metasoma*** (Figs 31, 32). Nearly parallel-sided in dorsal view, 1.9 × as long as wide and 1.4 × as long as mesosoma; first and second tergites densely and coarsely longitudinally rugose; second metasomal suture rather wide and slightly sinuate; third tergite 1.3 × longer than second tergite and straight, flat medially, broadly truncate posteriorly in dorsal view (slightly concave: Fig. 31), acute posteriorly in lateral view (Fig. 32), reticulate-rugose and with satin sheen (Fig. 31), lateral lamella narrow, wider latero-apically and medio-apically narrow; ovipositor sheath narrow, apically somewhat widened and darkened, its visible part 0.32 × as long as fore wing and 0.58 × metasomal carapace and sparsely setose part 0.07 × fore wing and with few erect setae; hypopygium of ♀ with long narrow triangular and spine-like protuberance apically (Fig. 32) and rather sparsely setose.

***Colour*.** Yellowish brown (including most of hind femur); apex of antenna, hind tibia apically and subbasally, parastigma, vein 1-M and apex of ovipositor sheath brown; stemmaticum dark brown; clypeus, palpi, tegulae, remainder of legs first and second tergites and metasoma ventrally pale yellowish or ivory; pterostigma (but basally and apically pale yellowish) dark brown; wing membrane slightly brownish below pterostigma.

##### Male.

Unknown.

##### Biology.

Unknown.

##### Distribution.

Yemen.

##### Etymology.

From *aspidiotes* (Greek for shield-bearer) because of the truncated shield-like third metasomal tergite.

#### Phanerotoma bilinea

Taxon classificationAnimaliaHymenopteraBraconidae

Lyle, 1924

E3080B30-01EA-55B9-A212-8AFD98161CBB

Figs 40–43 , 44–54

Phanerotoma bilinea Lyle, 1924: 101; Zettel, 1987: 364; van Achterberg, 1990: 24–25 (redescription, lectotype designation).
Bracotritoma bilinea ; Shenefelt, 1973: 909.
Phanerotoma gregori Snoflák, 1951: 13; Shenefelt, 1973: 916 (synonymised by Zettel (1987)).

##### Type material.

***Holotype*** ♀ of *P. gregori* (MMB); lectotype ♀ of *P. bilinea* (NHMUK; collected from a *Quercus* tree) .

##### Additional material.

**Yemen** (Ar Rujum, Malaise trap; Sana’a, Malaise, light and pitfall traps; Al Kadan, light trap; Ta’izz, light trap; 12 km NW of Manakhah, Malaise trap).

##### Diagnosis.

**Figures 40–43.**
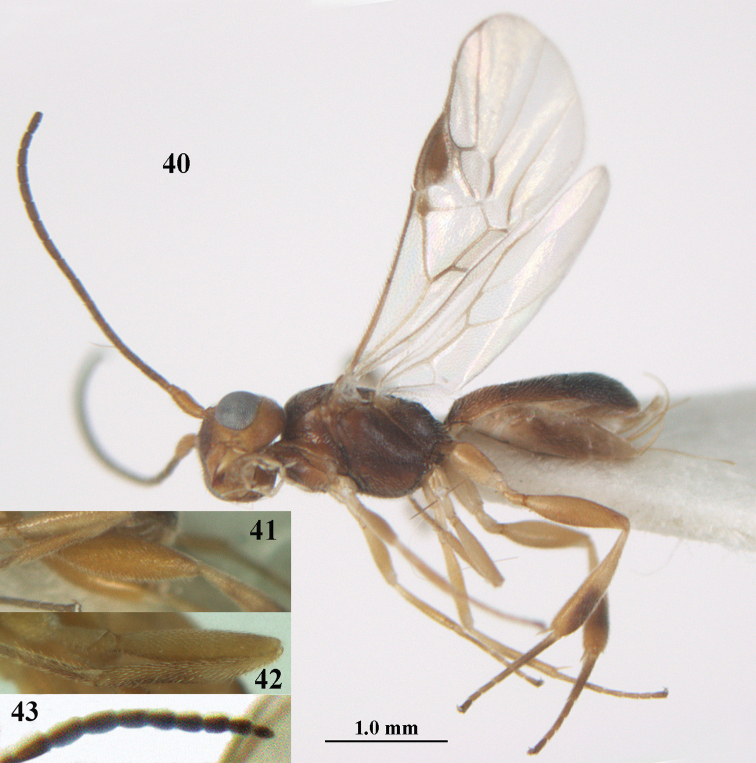
*Phanerotoma bilinea* Lyle, Yemen, ♀ (**40**) and ♂ (**41–43**) **40** habitus lateral **41** hind femur, lateral **42** middle tibia lateral **43** apical third of antenna lateral.

Hypopygium of ♀ with spine-like, long and acute narrow triangular protuberance (Figs 40, 47); fourth sternite of ♀ enlarged; inner tooth of mandible 0.4 × apical tooth (Fig. 49); vein r of fore wing 0.2–0.4 × as long as vein 3-SR; apical half of hind tibia with large dark brown patch laterally; medially third tergite 1.0–1.2 × longer than second tergite, tergite dark brown medially, rounded laterally and moderately concave medio-posteriorly in dorsal view (Fig. 46); ovipositor sheath narrow and only apically with some erect setae. Similar to *P. maculata* (Wollaston, 1858) from Madeira; but *P. bilinea* has the third metasomal tergite 1.0–1.2 × as long as the second tergite medially (1.4–1.7 × longer than second tergite in *P. maculata*) and its apical half distinctly sculptured and slightly shiny (at least partly smooth and distinctly shiny), middle tibia pale yellow (surroundings of blister dark brown) and vein 2-SR distinctly curved (straight).

##### Variations.

(Yemen). Length of fore wing of ♀ 2.3–3.0 mm, of ♂ 2.1–2.7 mm; parastigma and vein 1-M of fore wing yellowish, brown to rather dark brown; third metasomal tergite 1.0–1.2× as long as the second tergite medially; third and fourth antennal segments in ventral view distinctly darker than scapus and only very rarely similar brownish; apical half of hind tibia yellowish ventrally and outer side largely dark brown (but apically yellowish brown).

##### Biology.

Parasitoid of *Argyrotaenia ljungiana* (Thunberg, 1797) (Tortricidae) and *Prays citri* (Milliere, 1873) (Yponomeutidae).

##### Distribution.

Austria; Azerbaijan; Belgium; Czech Republic; France; Germany; Greece; Hungary; Japan; Korea; Moldova; Netherlands; Poland; Romania; Russia (including Far East Russia); Slovakia; Spain; Switzerland; Ukraine; United Kingdom; Serbia; *Yemen.

**Figures 44–54.**
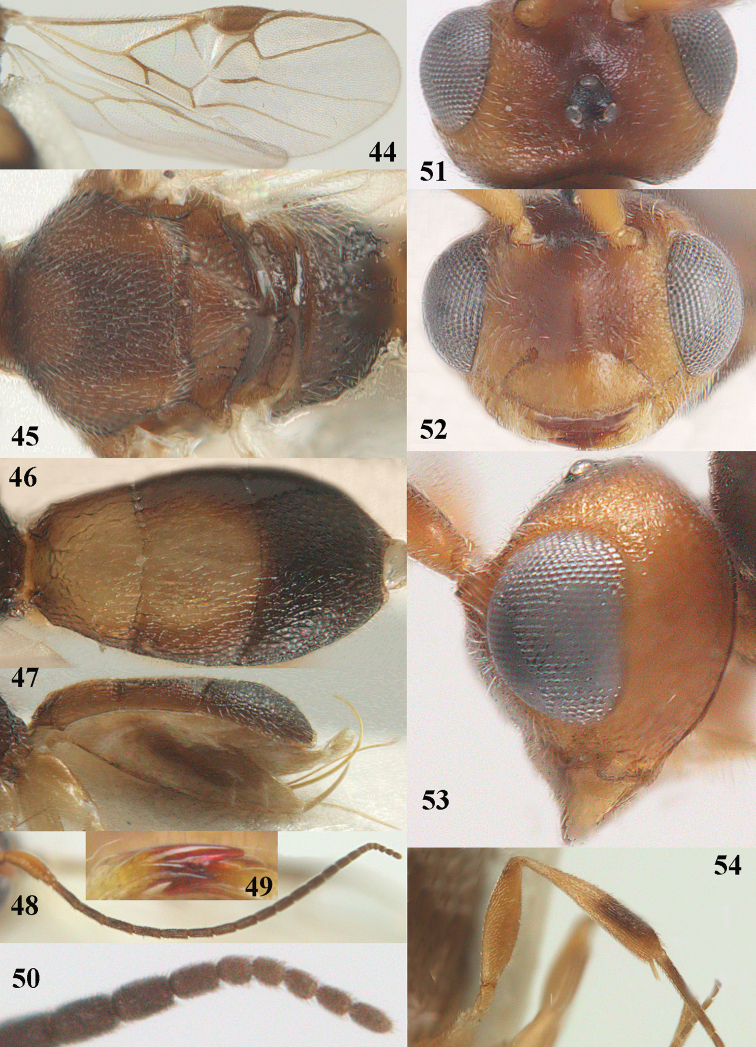
*Phanerotoma bilinea* Lyle, ♀, Yemen **44** wings **45** mesosoma dorsal **46** first–third metasomal tergites dorsal **47** metasoma lateral **48** antenna lateral **49** mandible ventral **50** apical third of antenna lateral **51** head dorsal **52** head anterior **53** head lateral **54** hind leg lateral.

#### Phanerotoma brunneivena sp. nov.

Taxon classificationAnimaliaHymenopteraBraconidae

45A7FD2B-BC9F-59B5-A107-88318F55B241

http://zoobank.org/3233C1CD-45CE-4ABB-8A4D-5841BFF2CAFC

Figs 55–57 , 58–68

##### Type material.

***Holotype***, ♀ (RMNH), “**United Arab Emirates**, Fujairah (1484), light trap, 19.iv.–2.v.2005, 25°08'N, 56°21'E, A. v. Harten, RMNH’06”. ***Paratypes***: 28♀, 1♂: Idem, 2.v.–5.vi.2005; 14♀: Idem, 19.iv.–2.v.2005; 4♀: Idem, 5–24.iii.2005; 3♀, 1♂: Idem, 24.iii.–6.iv.2005; 7♀, 2♂: Idem, 13–19.iv.2005; 4♀: Idem, 2–13.v.2005; 1♀: Idem, 16–24.ii.2005; 3♀: Idem, 13–29.xi.2005; 2♀: Idem, 29.xi.2005–2.i.2006; 1♀: Idem, 2–13.v.2005; 1♀: Idem, 24.ii.–5.iii.2005; 1♀: “United Arab Emirates, Sharjah Desert Park (1556), light tr[ap], 30.iv.–7.v.2005, 25°17'N, 55°42'E, A. v. Harten, RMNH’06”; 1♀: Idem, 21–28.v.2007; 1♀: Idem, 20–21.iv.2006, M. Fibiger; 1♀: “Wadi Safad (5015), white & yellow pan tr., 2–26.i.2006, 25°13'N, 56°19'E, A. v. Harten, RMNH’06”; 1♀: “Wadi Maidaq (3808), at light, 21.xii.2005–2.ii.2006, 25°18'N, 56°07'E, A. v. Harten, RMNH’06”; 2♀: Idem, 27.iv.–4.v.2006; 1♀: “United Arab Emirates, Sharjah x Khor Kalba (3851), light trap, 24°59'N, 56°09'E, 17–18.iv.2006, M. Fibiger, RMNH’06”; 1♀: “United Arab Emirates, NARC near Sweihan (1299), light trap, 9–20.iv.2005, 24°24'N, 55°26'E, A. v. Harten, RMNH’06”; 1♀, “**Yemen** (7501), Al Kadan, light trap, i.2003, A. v. Harten & T. Abdul-Haq, RMNH’03”; 1♀: Idem, x.2001; 1♀: Idem, xi.2001; 1♀: Idem, v.2002; 1♀, “Yemen, Al Kowd, light tr[ap], v.–vi.2000, no. 4719, A. v. Harten & S. Al Haruri, RMNH’01”.

##### Comparative diagnosis.

Very similar to *P. flavivena* because of the needle-shaped ovipositor sheath of which the apical half is yellow, the yellow pterostigma, the long third tergite (1.8–2.1 × as long as second tergite), the distinctly convex temple and short inner tooth of the mandible. It differs by the rugulose or striate and matt to slightly shiny temple (mostly smooth and shiny in *P. flavivena*), the dark brown vein r of fore wing ca. 0.2 × as long as vein 3-SR (yellow and 0.4–0.7 ×), the vein SR1 of fore wing distinctly curved (straight or nearly so) and the longer apical triangular appendage of the hypopygium.

##### Description.

Female, holotype, length of body (excluding ovipositor) 5.7 mm; antenna 3.8 mm (but apical segment missing); fore wing 3.9 mm; visible part of ovipositor sheath 0.85 mm (only apex setose).

**Figures 55–57.**
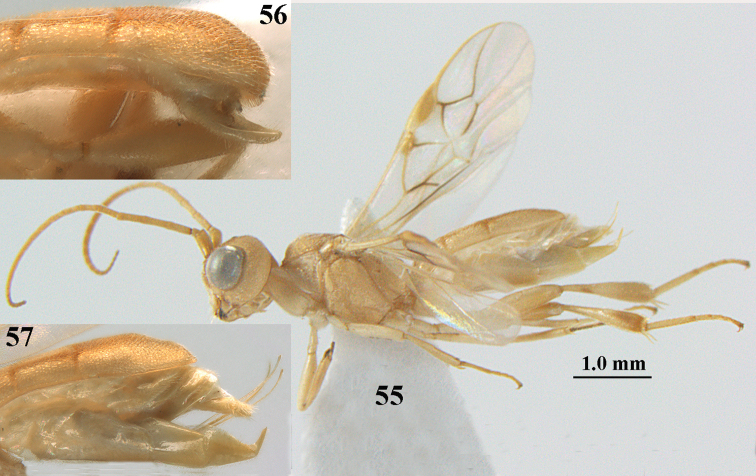
*Phanerotoma brunneivena* van Achterberg, sp. nov., ♀, holotype (but **57** of paratype and **56** of paratype ♂) **55** habitus lateral **56, 57** apical half of metasoma lateral.

***Head*.** Width 1.5 × median length in anterior view and part of head above eye in lateral view 0.3 × height of eye (Fig. 65); antenna with 23 segments and 1.2 × longer than fore wing, segments gradually shortened, narrowed apically and five apical segments moniliform (Fig. 67), third, fourth and penultimate segments 3.2, 3.0 and 1.4 × longer than wide in lateral view, respectively; area of stemmaticum coriaceous; OOL: diameter of posterior ocellus: POL = 20: 4: 10; length of eye 1.7 × temple in dorsal view (Fig. 63); frons coarsely rugose and with median carina; vertex coarsely and densely reticulate-rugose, and rather dull; temple densely and finely rugose, dull; face coarsely rugose and without distinct median ridge or median carina; clypeus mostly smooth, rather shiny and with three minute teeth medio-ventrally, partly hidden by conspicuous fringe (Fig. 64); eye medium-sized, strongly convex and in lateral view 1.3 × (measured medially) temple (Fig. 65), in anterior view 0.8 × minimum width of face; upper condyle of mandible near lower level of eyes (Fig. 64); malar space rugose, shiny and 0.5 × as basal width of mandible; lower tooth of mandible 0.1 × as long as apical tooth (Fig. 68).

**Figures 58–68.**
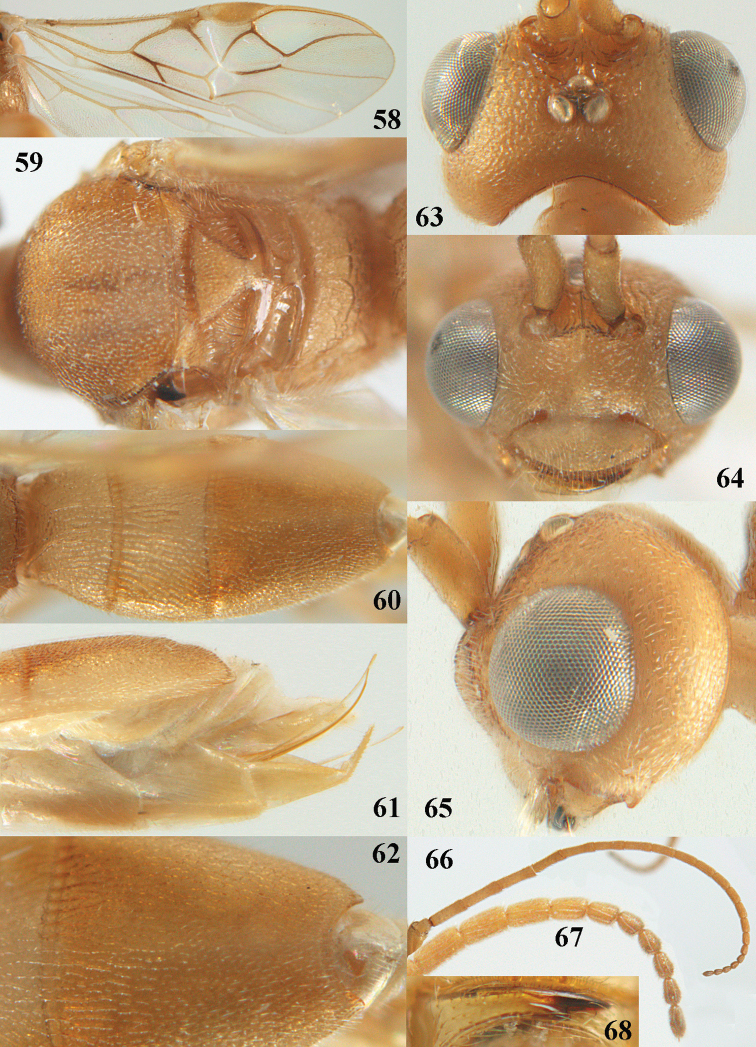
*Phanerotoma brunneivena* van Achterberg, sp. nov., ♀, holotype **58** wings **59** mesosoma dorsal **60** first–third metasomal tergites dorsal **61** metasoma lateral **62** apex of third metasomal tergite dorsal **63** head, dorsal **64** head anterior **65** head lateral **66** antenna lateral **67** apical third of antenna lateral **68** mandible ventral.

***Mesosoma*** (Figs 55, 59). Length 1.5 × its width in lateral view; side of pronotum mainly rugose, but dorsally nearly smooth; mesoscutum densely rugose and rather shiny, densely setose; notauli hardly indicated; scutellar sulcus wide and with seven carinae (Fig. 59); scutellum triangular, largely punctate and rather shiny; metanotum with short median carina anteriorly and small tooth protruding posteriorly; propodeum coarsely reticulate-rugose, without distinct median and transverse carinae, latero-posteriorly tuberculate. ***Wings*.** Fore wing 2.6 × longer than its maximum width; length of 1-R1 1.4 × as long as pterostigma; r issued far beyond middle of pterostigma and 0.2 × 3-SR; 2-SR curved and distally subparallel with posterior margin of pterostigma (Fig. 58); SR1 curved; 2-SR+M absent because of interstitial m-cu; parastigma large; 1-CU1 0.5 × as long as vein 2-CU1; r:3-SR:SR1 = 2:10:25; 2-SR:3-SR:r-m = 25:10:7; r-m reclivous; 2-M weakly curved (Fig. 58). Hind wing: M+CU:1-M:1r-m = 21:20:10; r feebly indicated. ***Legs*.** Hind femur 3.4 × as long as wide and widened subbasally; middle tibia with small ivory blister; inner spur of middle tibia 0.5 × its basitarsus; hind coxa mostly smooth and shiny.

***Metasoma*** (Figs 57, 60–62). Elongate elliptical in dorsal view, twice as long as wide and 1.7 × as long as mesosoma; first and second tergites coarsely longitudinally rugose and rugae with interconnections; third tergite 2.1 × longer than second tergite and laterally nearly straight, in lateral view rather flat, densely reticulate-rugulose and medio-posteriorly distinctly concave (Figs 60, 62), lateral lamella narrow, protruding latero-apically and medio-apically absent (Fig. 62); ovipositor sheath narrow, needle-shaped (Fig. 61), its visible part 0.22 × as long as fore wing and 0.38 × metasomal carapace and only its apex with small cluster of setae; hypopygium apically with medium-sized and slender bent up triangle, without apical spine and with medium-sized sparse setae.

***Colour*.** Pale brownish yellow; palpi, mandible (except dark brown teeth), clypeus, pronotum, legs (but hind tibia rather darkened apically) first and second tergites largely and metasoma ventrally pale yellow or ivory; apical antennal segments and stemmaticum brown; pterostigma brownish yellow but anteriorly pale yellowish (Fig. 58); wing membrane subhyaline; parastigma (but brownish posteriorly), veins 1-M, 2-CU1 and m-cu of fore wing yellow and veins r, 1-CU1, cu-a, 2-SR, 3-SR and 2-M dark brown.

##### Male.

Similar to female but third tergite narrowed posteriorly, rounded and convex apically (Fig. 56; one male has a subapical bump) and pterostigma (except anteriorly) brown.

##### Variations.

Length of fore wing of ♀ 3.1–4.3 mm, of ♂ 3.0–3.5 mm; antenna of ♀ with 23 segments; third tergite 1.8–2.1 × longer than second tergite, of ♀ medio-apically concave or obtuse and with no or a narrow lamella, flat or with a small subapical bump; ovipositor sheath up to 0.36 × as long as fore wing if exserted; rarely scutellum laterally and propodeum apically dark brown, wing membrane below pterostigma and near vein 1-M somewhat infuscate; third tergite rarely dark brown basally or with dark triangle; temple and mesoscutum medially pale brownish yellow or ivory; sometimes pterostigma darkened but anteriorly pale yellowish.

##### Biology.

Unknown.

##### Distribution.

United Arabian Emirates, Yemen.

##### Etymology.

Named after the brownish vein r of the fore wing (*brunneus* is Latin for brown).

#### Phanerotoma caudatoides sp. nov.

Taxon classificationAnimaliaHymenopteraBraconidae

6EE236B6-6946-51C0-9CF3-2C702522A5EA

http://zoobank.org/307297BB-2C47-44D7-A48B-4760766E8128

Figs 69–70 , 71–81

##### Type material.

***Holotype***, ♀ (RMNH), “**Yemen** (6381), Ta’izz, light tr[ap], x.2000, A. van Harten & A.R. Al Yarimi, RMNH’02”.

**Figures 69, 70.**
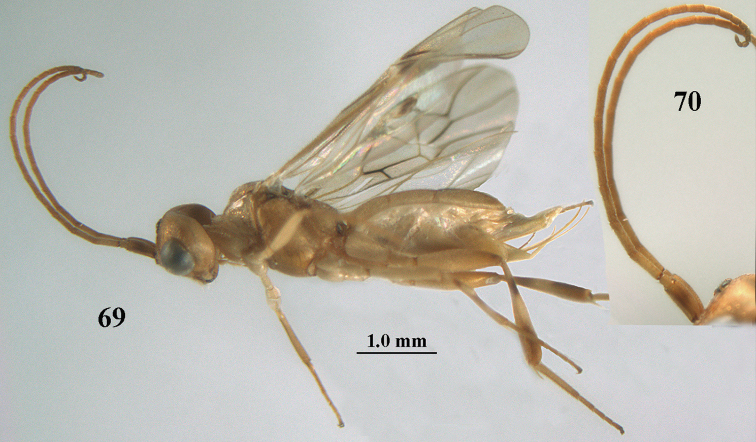
*Phanerotoma caudatoides* van Achterberg, sp. nov., ♀, holotype **69** habitus lateral **70** antenna lateral.

##### Diagnosis.

**Figures 71–81.**
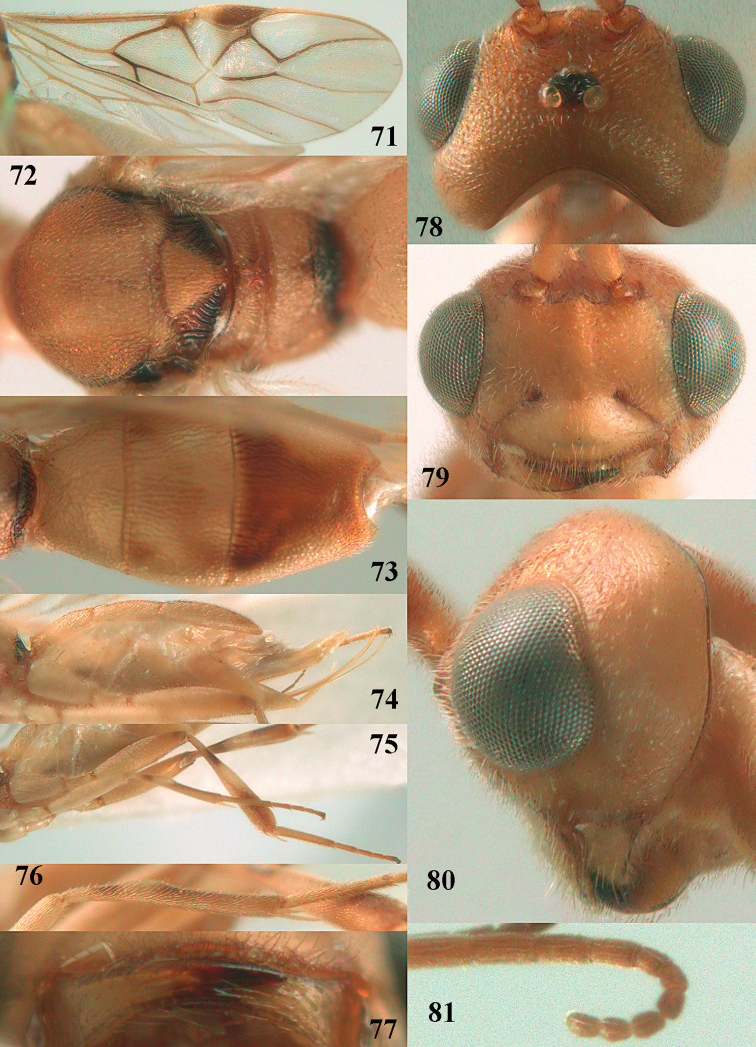
*Phanerotoma caudatoides* van Achterberg, sp. nov., ♀, holotype **71** wings **72** mesosoma dorsal **73** first–third metasomal tergites dorsal **74** metasoma lateral **75** hind leg lateral **76** middle tibia lateral **77** mandible ventral **78** head, dorsal **79** head anterior **80** head lateral **81** apical third of antenna lateral.

Hypopygium of ♀ with long spine-like, acute triangular protuberance (Fig. 74); ocelli medium-sized (POL shorter than diameter of posterior ocellus; Fig. 78); medially third tergite 1.0–1.2 × longer than second tergite, medio-posteriorly yellowish brown and widely excavated (Fig. 73), laterally nearly straight (Fig. 73); width of clypeus 0.9 × minimum width of face; pale basal part of pterostigma medium-sized and contrasting with dark brown middle of pterostigma (Fig. 71); head distinctly excavated posteriorly (Fig. 78); vein cu-a ca. 0.7 × as long as vein 1-CU1; vein 2-SR straight; vein 1-SR+M approx. as wide as vein 2-SR (Fig. 71); whitish blister of middle tibia medium-sized and contrasting with its rather dark brown surroundings (Fig. 76); apical half of hind tibia with large dark brown patch laterally and yellowish or brownish ventrally; inner tooth of mandible ca. 0.5 × apical tooth (Fig. 77); third tergite of ♀ at least partly dark brown medially and pale yellowish laterally; exerted ovipositor sheath 0.5–0.6 × length of metasomal carapace; length of body of ♀ ca. 5 mm. Very similar to *P. caudata* Granger, 1949, from Madagascar, but *P. caudatoides* has vein 2-CU1 of fore wing curved (above level of 1-CU1), vein cu-a approx. ½ as long as vein 1-CU1, vein 2-SR evenly and strongly curved, vein 1-SR+M narrower than vein 2-SR and ocelli rather small (POL equal to diameter of posterior ocellus) and length of third metasomal tergite 0.7 × basal width and laterally slightly curved. Also related to *P. nitidiventris* Zettel, 1990, from South Africa and Ethiopia; the latter is a new record and based on specimens in RMNH collected by C.J. Zwakhals. *Phanerotoma nitidiventris* has the third tergite 1.4–1.6 × as long as second tergite, vein 1-CU1 of fore wing widened, antenna (except scapus and pedicellus) often dark brown and vein 2-CU1 of fore wing curved (above level of 1-CU1). The name is confusing because only the antenna (except scapus and part of pedicellus) are usually blackish ventrally.

##### Description.

Female, holotype, length of body (excluding ovipositor) 4.9 mm; antenna 4.5 mm; fore wing 4.1 mm; visible part of ovipositor sheath 1.2 mm (only at apex some erect setae).

***Head*.** Width 1.7 × median length in anterior view and part of head above eye in lateral view 0.4 × height of eye (Fig. 80); antenna with 23 cylindrical segments, slender medially and 1.1 × longer than fore wing, five apical antennal segments small and moniliform (Fig. 81), with short bristle apically and apical segment without spine, third, fourth and penultimate segments 4.2, 4.0 and 1.3 × longer than wide in lateral view, respectively; area of stemmaticum mainly superficially granulate; OOL: diameter of posterior ocellus: POL = 17: 5: 5; length of eye 1.7 × temple in dorsal view (Fig. 78); frons with few longitudinal rugae medially, with satin sheen, rugose laterally and without median carina; vertex rugose and with satin sheen; temple longitudinally rugose, rather shiny, gradually narrowed behind eyes; face transversely or obliquely rugose and with obsolescent median bump and rather shiny; clypeus smooth, convex medially, shiny and 0.9 × minimum width of face, intertentorial distance 2.6 × minimum width between clypeus and eye, long erect setose and with 3 indistinct blunt teeth medio-ventrally (Fig. 77); eye medium-sized, strongly convex and in lateral view 1.6 × wider than temple (measured medially; Fig. 80), in anterior view its height 0.7 × minimum width of face; upper condyle of mandible near lower level of eyes (Fig. 79); malar space rugose, rather shiny and 0.5 × as long as basal width of mandible; lower tooth of mandible 0.5 × length of apical tooth, robust (Fig. 77).

**Figures 82–84.**
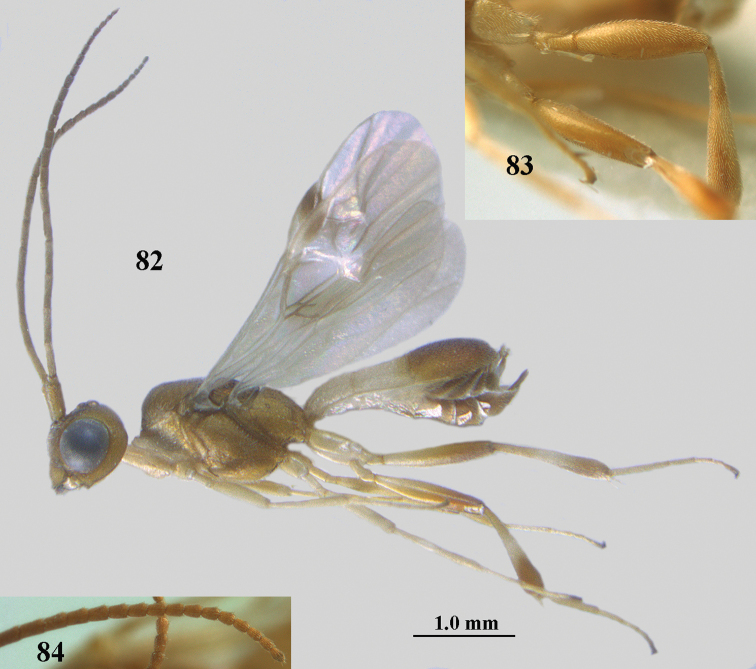
*Phanerotoma ejuncida* van Achterberg, sp. nov., ♀ holotype (**82**) and ♂ paratype (**83, 84**) **82** habitus lateral **83** hind femur and tibia lateral **84** apical half of antenna lateral.

***Mesosoma*** (Figs 69, 72). Length 1.4 × its width in lateral view; side of pronotum rugose medially and posteriorly, coriaceous antero-laterally and dorsally partly smooth; propleuron posteriorly convex and converging to median sulcus; mesosternum superficially granulate and with satin sheen; mesoscutum densely and finely rugulose, rather dull; scutellum slightly convex, longitudinally rugose and rather matt; scutellar sulcus rather wide, with nine carinae (Fig. 72); metanotum with median carina medially, posteriorly with small tooth and finely serrate posteriorly; propodeum rugose, anterior face long, with transverse carina, no median carina, and latero-posteriorly weakly tuberculate. ***Wings*.** Fore wing 3.3 × longer than its maximum width; 1-R1 1.5 × as long as pterostigma; distance between wing apex and marginal cell apex 0.2 × length of 1-R1; r issued far beyond middle of pterostigma and 0.2 × 3-SR; 2-SR strongly curved and subparallel with posterior margin of pterostigma (Fig. 71); SR1 curved; m-cu interstitial; parastigma large; 1-CU1 0.4 × as long as vein 2-CU1, cu-a 0.7 × 1-CU1; r:3-SR:SR1 = 5:25:70; 2-SR:3-SR:r-m = 32:25:8; r-m reclivous; 2-M slightly curved, oblique (Fig. 71). Hind wing: M+CU:1-M:1r-m = 14:12:10. ***Legs*.** Hind femur matt, 3.8 × as long as wide and robust; middle tibia slender and with slightly developed pale yellowish blister; inner spur of middle tibia 0.4 × its basitarsus; hind tibia slender (Fig. 75); hind coxa largely coriaceous and matt, but dorsally mostly smooth and rather shiny.

***Metasoma*** (Figs 73, 74). Nearly parallel-sided in dorsal view, 1.8 × as long as wide and 1.4 × as long as mesosoma; first and second tergites densely irregularly longitudinally rugose; second metasomal suture medium-sized and straight; third tergite 1.2 × longer than second tergite and straight laterally, convex medially, deeply emarginate posteriorly in dorsal view (Fig. 73), acute posteriorly in lateral view (Fig. 74), densely reticulate-rugose and with satin sheen (Fig. 73), lateral lamella wide laterally and latero-apically but medio-apically narrow; ovipositor sheath narrow, apically parallel-sided and pale yellowish, its visible part 0.32 × as long as fore wing and 0.58 × metasomal carapace and sparsely setose part 0.3 × fore wing and only apically with few erect setae; hypopygium of ♀ with long narrow triangular and in lateral view spine-like protuberance apically (Fig. 74) and rather sparsely setose.

***Colour*.** Brownish yellow (including most of hind femur, hind tarsus largely, tibiae subbasally and apically brown); apex of antenna and parastigma posteriorly brownish; stemmaticum, scutellum laterally, posterior face of propodeum, pterostigma (but basally and apically pale yellowish), vein 1-M and triangular patch of third tergite dark brown; remainder of third tergite brown medio-posteriorly and laterally pale yellowish; clypeus, palpi, pronotum, tegulae, remainder of legs, first and second tergites, and metasoma ventrally pale yellowish or ivory; wing membrane with brown patch below pterostigma and first subdiscal cell brownish.

##### Male.

Unknown.

##### Biology.

Unknown.

##### Distribution.

Yemen.

##### Etymology.

The suffix -*oides* (Greek for like, resembling) is added to the specific name *caudata*, indicating a similarity to *P. caudata* Granger.

#### Phanerotoma ejuncida sp. nov.

Taxon classificationAnimaliaHymenopteraBraconidae

CF54325A-2957-5368-8599-437FBC23A0F6

http://zoobank.org/F61F21D0-2A53-4651-BA54-E29135EEA19B

Figs 82–84 , 85–95

##### Type material.

***Holotype***, ♀ (RMNH), “**United Arab Emirates**, Wadi Wurajah Farms (10802), light trap, 8–15.iii.2009, 25°5'N, 56°13'E, A. v. Harten, RMNH’09”. ***Paratypes***: 2♀: Idem, 15.i.–22.ii.2009; 1♀: Idem, 22.ii.–2.iii.2009; 2♀, 1♂: “United Arab Emirates, NARC near Sweihan (1245), light trap, 28.iii.–2.iv.2005, 24°24'N, 55°26'E, A. v. Harten, RMNH’06”; 1♂: Idem, 20–30.iv.2005.

##### Diagnosis.

**Figures 85–95.**
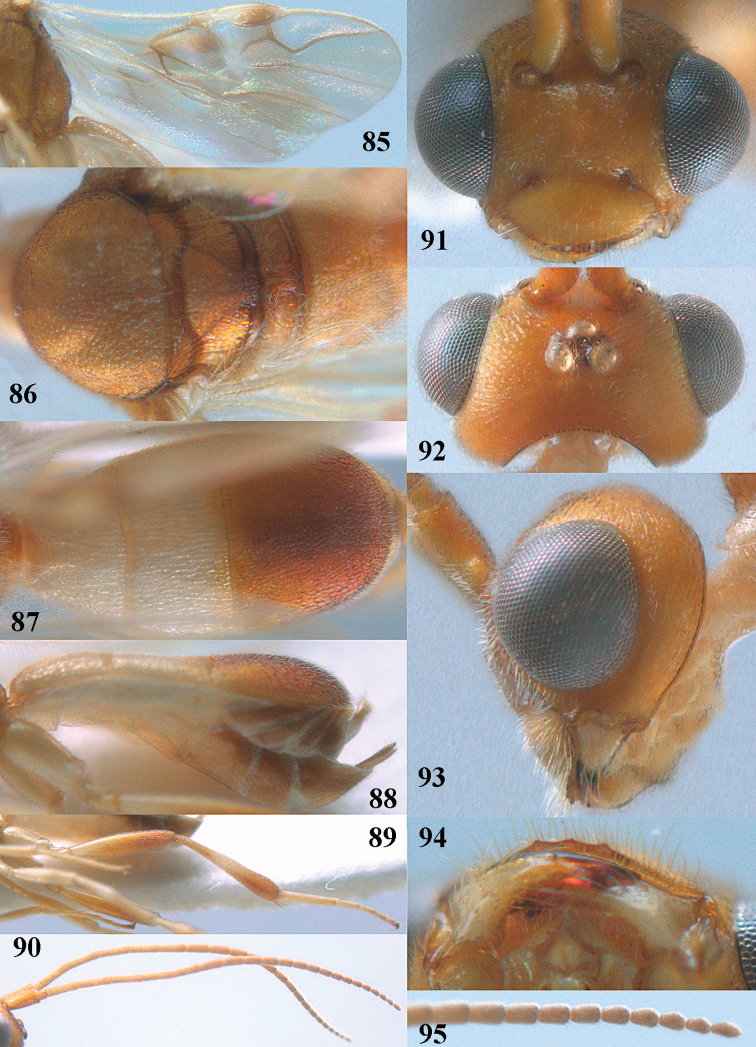
*Phanerotoma ejuncida* van Achterberg, sp. nov., ♀, holotype **85** wings **86** mesosoma dorsal **87** first–third metasomal tergites dorsal **88** metasoma lateral **89** hind leg lateral **90** antennae lateral **91** head anterior **92** head dorsal **93** head lateral **94** mandible ventral **95** apical third of antenna lateral.

Third tergite 1.5–1.8 × as long as second tergite, curved laterally, densely sculptured, rather dull and convex, its posterior lamella wide and truncate medio-apically (Fig. 87); subapical antennal segments somewhat serrate in lateral view because of small subapical protuberances, sixth segment from apex narrowed basally and subapically widened and subapical segments rather long (Fig. 95); fourth antennal segment approx. 4.5 × as long as wide; ventral half of temple matt to slightly shiny, granulate, rugulose or striate; face nearly entirely densely sculptured and shiny; frons rugose; clypeus approx. as wide as face, intertentorial distance 4–5 × minimum distance between clypeus and eye ventrally, and very shiny (Fig. 91); vein r of fore wing non-linear with vein 3-SR (Fig. 85); OOL aciculate; vertex rather shiny (Fig. 92); vein 1-CU1 of fore wing short compared to long and strongly oblique vein cu-a (vein cu-a 1.6–2.2 × as long as vein 1-CU1; Fig. 85); hind femur of ♀ elongate (Fig. 89); tarsal claws slender; length of fore wing 3.0–4.1 mm.

##### Description.

Female, holotype, length of body (excluding ovipositor) 5.1 mm; antenna 4.1 mm; fore wing 3.7 mm; visible part of ovipositor sheath 0.3 mm (setose part 0.2 mm).

***Head*.** Width 1.5 × median length in anterior view and part of head above eye in lateral view 0.3 × height of eye (Fig. 93); antenna with 23 segments and 1.2 × longer than fore wing, segments slender and gradually shortened, segments of apical half with minute subapical protuberances and widened apically (“pseudoserrate”; Fig. 95), six apical segments moniliform and narrowed basally (Figs 90, 95), third, fourth and penultimate segments 4.4, 4.4 and 1.7 × longer than wide in lateral view, respectively; area of stemmaticum aciculate; OOL: diameter of posterior ocellus: POL = 19: 10: 5; length of eye 2.1 × temple in dorsal view (Fig. 92); frons with coarse curved rugae, shiny and without median carina; vertex transversely rugose and shiny; temple longitudinally rugose and shiny, convex; clypeus approx. as wide as face (intertentorial distance ca. 4.0 × minimum distance between clypeus and eye ventrally), convex, mostly smooth and shiny (Fig. 91); face shiny and distinctly rugose, with short indistinct median ridge; clypeus with three obsolescent teeth medio-ventrally (Fig. 94); eye large, strongly convex and in lateral view 2.2 × (measured medially) temple (Fig. 93), in anterior view 0.9 × minimum width of face; upper condyle of mandible near lower level of eyes (Fig. 91); malar space aciculate, shiny, and 0.3 × as basal width of mandible; lower tooth of mandible robust and 0.5 × as long as apical tooth (Fig. 94).

***Mesosoma*** (Figs 82, 86). Length 1.5 × its width in lateral view; side of pronotum distinctly rugose; mesosternum smooth and very shiny; mesoscutum finely reticulate-rugose and rather shiny; notauli slightly indicated anteriorly; scutellar sulcus wide medially and narrow laterally, with eleven carinae (Fig. 86); scutellum widely triangular, densely finely rugose (nearly up to posterior margin), convex and rather shiny; metanotum with short median carina anteriorly and truncate posteriorly; propodeum coarsely reticulate-rugose, without distinct median and transverse carinae, latero-posteriorly not tuberculate. ***Wings*.** Fore wing 3.0 × longer than its maximum width; length of 1-R1 1.2 × as long as pterostigma; r issued rather far beyond middle of pterostigma and 0.55 × 3-SR; distance between 1-R1 and wing apex 0.3 × 1-R1; 2-SR bent and distally subparallel with posterior margin of pterostigma (Fig. 85); SR1 curved; 2-SR+M absent because of narrowly antefurcal m-cu; parastigma large; 1-CU1 0.2 × as long as vein 2-CU1, cu-a strongly inclivous and approx. twice as long as 1-CU1; r:3-SR:SR1 = 11:20:77; 2-SR:3-SR:r-m = 34:20:11; r-m reclivous; 2-M weakly curved (Fig. 8). Hind wing: M+CU:1-M:1r-m = 25:23:10; cu-a narrow. ***Legs*.** Hind femur slender, 5.0 × as long as wide and hardly widened (Fig. 89); middle tibia with ivory blister; inner spur of middle tibia 0.5 × its basitarsus; hind coxa mostly smooth and shiny; hind tibia and basitarsus slender (Fig. 89); tarsal claws slender.

***Metasoma*** (Figs 87, 88). Oval in dorsal view, 1.9 × as long as wide and 1.4 × as long as mesosoma; first and second tergites finely and densely longitudinally rugose; third tergite 1.8 × longer than second tergite and laterally curved, in lateral view rather convex, densely reticulate-rugulose and medio-posteriorly truncate (Fig. 87), lateral lamella narrow, not protruding latero-apically and medio-apically truncate and wide; ovipositor sheath moderately widened apically (Fig. 88), its visible part 0.07 × as long as fore wing and 0.12 × metasomal carapace and setose part 0.05 × fore wing, setae erect and medium-sized or long; hypopygium apically acute, lacking an up curved triangle or apical spine (Fig. 88).

***Colour*.** Brownish yellow (including tegulum); palpi, mandible (except dark brown teeth), clypeus, malar space, prothorax, legs (but apical half of middle tibia, hind femur largely, and hind tibia apically and basally brownish), first and second metasomal tergites and basal half of metasoma ventrally pale yellow or ivory; apical third of antenna, humeral plate, apex of ovipositor sheath and stemmaticum brown; pterostigma dark brown, but antero-basally and narrowly apically pale yellowish (Fig. 85); wing membrane subhyaline but below dark part of pterostigma and first subdiscal cell slightly infuscate; parastigma and vein m-cu largely pale yellow; apical half of metasoma, veins 1-M, basal half of 2-CU1, r and 3-SR of fore wing rather dark brown.

##### Male.

Similar to female (including antenna: Fig. 84), but hind femur moderately widened (3.3–3.5 × as long as wide; Fig. 83); vein r and pterostigma largely pale yellowish; third tergite brownish and 1.5–1.7 × as long as second tergite.

##### Variations.

Length of fore wing 3.0–4.1 mm; third tergite 1.5–1.8 × longer than second tergite; vein 1-R1 of fore wing 1.1–1.2 × as long as pterostigma; vein 1-M of fore wing varies from pale yellow to brownish; vein cu-a of fore wing 1.6–2.2 × as long as vein 1-CU1.

##### Biology.

Unknown.

##### Distribution.

United Arabian Emirates.

##### Etymology.

Named after its slender hind femur (*ejuncidus* is Latin for slender).

#### Phanerotoma flavivena

Taxon classificationAnimaliaHymenopteraBraconidae

Edmardash & Gadallah, 2019

E968E2DC-3FE0-55CA-8B28-9A1642750B1F

Figs 96–98 , 99–108

Phanerotoma flavivena Edmardash & Gadallah, 2019: 359–364.

##### Type material.

***Paratypes***: 6♀: “**Yemen**: Al Kowd (3901), vii.1999, light trap, A. v. Harten & S. Al Haruri, RMNH’00”; 3♀: Idem, viii.1999; 4♀: Idem, xii.1999; 5♀: Idem, i.2000; 4♀: Idem, ii.2000; 3♀: Idem, iii.2000; 5♀: Idem, iv.2000; 1♀: Idem, iv.2000; 13♀: Idem, v.–vi.2000; 1♀: Idem, vii.2000; 1♀: Idem, viii.2000; 7♀: Idem, xi.2000; 1♀: Idem, x.2000; 1♀: Idem, ii.2001; 1♀: Idem, iii.2001; 4 ♀: Idem, iv.2001; 3♀: Idem, 8–12.vii.2001; 4♀: Idem, 17–21.vii.2001; 1♀: Idem, 6–10.viii.2001; 4♀: Idem, 16–20.viii.2001; 4♀, 1♂: Idem, 27–31.vii.2001; 3♀: Idem, vii.–ix.2001; 1♂: Idem, 21–25.viii.2001; 2♀: Idem, 1–5.ix.2001; 1♀: Idem, i.–iii.2003; 6♀: Idem, Al Kowd, ix.2003; 1♀: “Yemen: Ta’izz (3066), light trap, xi.1999, A. van Harten & Ahmad Ahwad, RMNH’99”; 1♀: “Yemen: Ar Rujum (5700), 9.iv.–5.vi.2001, Mal. trap, A. v. Harten, RMNH’02”; 1♀: “Yemen: Al Kadan (7501), i.2003, light trap; A. v. Harten & T. Abdul-Haq, RMNH’03”; 2♀: “Yemen: Hamman’Ali (5404), from coffee-berries, 14.iii.2001, A. v. Harten, RMNH’02”.

**Figures 96–98.**
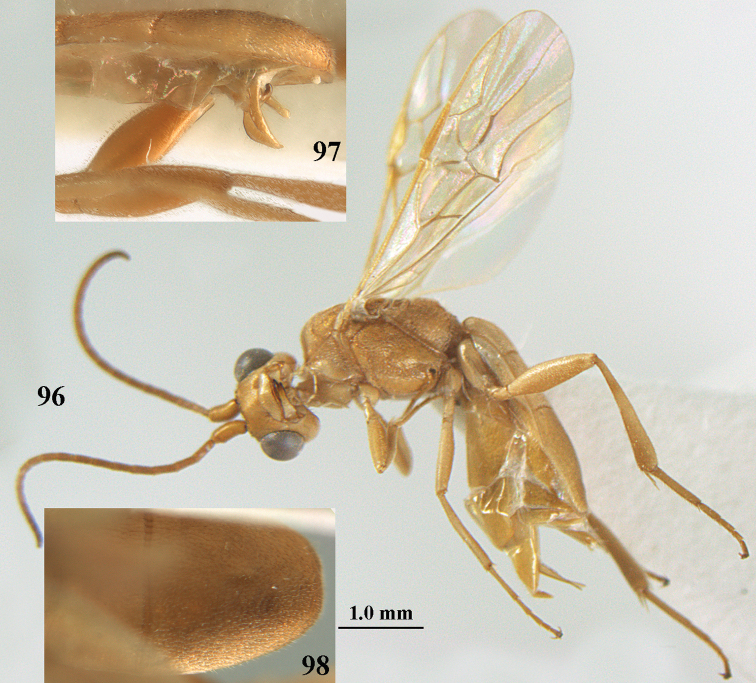
*Phanerotoma flavivena* Edmardash & Gadallah, paratype, ♀ (but **97, 98** of ♂ paratype), Yemen **96** habitus, lateral **97** metasoma and hind femur lateral **98** third metasomal tergite dorsal.

##### Diagnosis.

Third tergite 1.8–2.1 × as long as second tergite, straight laterally, rather shiny and flattened, its posterior lamella more or less concave medio-apically (Fig. 101); third tergite of male similar but more convex in lateral view (Fig. 97); antenna of ♀ with 23 segments and apical segments non-moniliform (Fig. 103); inner tooth of mandible 0.3 × apical tooth (Fig. 104); ventrally clypeus without teeth and with narrow truncate lamella; vein r of fore wing 0.4–0.7 × as long as vein 3-SR; vein m-cu of fore wing interstitial or antefurcal; eye in dorsal view 1.7–2.5 × as long as temple and 1.6–1.9 × in lateral view; temple smooth and shiny face medially smooth or nearly so; temple distinctly convex; scutellar sulcus narrow; parastigma, vein 1-M of fore wing, pterostigma entirely yellow; ovipositor sheath narrow, needle-shaped (Fig. 102); hypopygium acute, without spine apically and with short apical triangle bent upwards. If vein 1-R1 of fore wing approx. ½ as long as pterostigma, cf. *P. cyrenaica* Masi, 1932, from N. Africa. The male is very similar to female (Figs 97, 98); hind femur rather widened (Fig. 97), third tergite more convex in lateral view than of female (Fig. 97), vein 1-R1 of fore wing brown and antennal segments slenderer.

**Figures 99–108.**
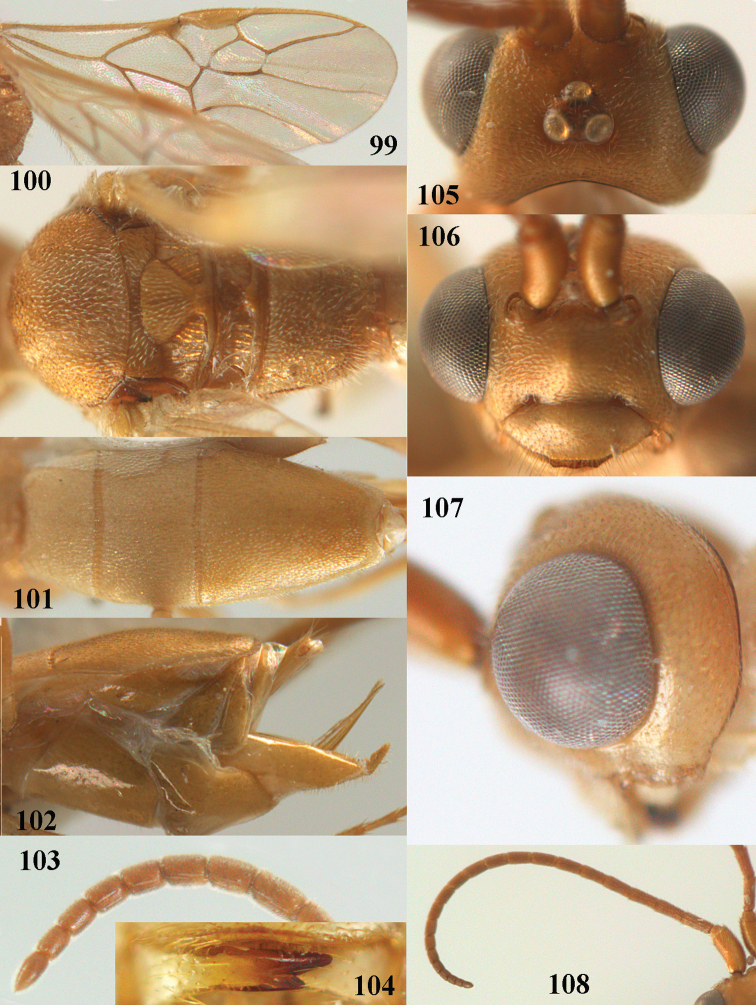
*Phanerotoma flavivena* Edmardash & Gadallah, ♀, paratype, Yemen **99** wings **100** mesosoma dorsal **101** first–third metasomal tergites dorsal **102** metasoma lateral **103** apical third of antenna lateral **104** mandible ventral **105** head dorsal **106** head anterior **107** head lateral **108** antenna lateral.

##### Variations.

Length of fore wing of ♀ 3.4–4.2 (of ♂ 3.2) mm; vein 2-SR of fore wing straight or weakly curved; lateral lamella of third tergite concave medio-apically, rarely only slightly sinuate; third tergite of ♂ 1.9–2.1 × longer than second tergite.

##### Biology.

Unknown, but two females have been reared from a lepidopterous host in coffee-berries.

##### Distribution.

Egypt, Yemen.

#### Phanerotoma glabritemporalis sp. nov.

Taxon classificationAnimaliaHymenopteraBraconidae

E72A86AA-7787-5DF0-9DAF-2E3762D31CF6

http://zoobank.org/55610506-40F9-4FCD-8146-69ED7E512FA3

Figs 109–111 , 112–123

##### Type material.

***Holotype***, ♀ (RMNH): “**United Arab Emirates**, al-Ajban (11858), light trap, 17.iv.–29.v.2006, 24°36'N, 55°01'E, A. v. Harten, RMNH’10”. ***Paratypes***: 2♀: Idem; 4♀, 1♂: Idem, 27.v.–26.vi.2006; 1♀: Idem, 17.x.–9.xi.2005; 8♀: “United Arab Emirates, NARC near Sweihan (1245), light trap, 28.iii.–2.iv.2005, 24°24'N, 55°26'E, A. v. Harten, RMNH’06”; 14♀, 1♂: Idem, 2–9.iv.2005; 12♀: Idem, 9–20.iv.2005; 1♀: Idem, 14–28.iii.2005; 6♀: Idem, 1.ii.–14.iii.2005; 1♀: Idem, 20–30.iv.2005; 1♀: “United Arab Emirates, Fujairah (1587), hand coll., 2–13.v.2005, 25°08'N, 56°21'E, A. v. Harten, RMNH’05”; 2♀: “**Yemen** (3901), Al Kowd, light trap, vii.1999, A. van Harten & S. Al Haruri, RMNH’00”; 1♀: Idem, ix.1999; 1♀: Idem, v.–vi.2000; 2♀: Idem, viii.1999; 21♀, 1♂: Idem, 1–5.ix.2001; 1♀: Idem, vi.2002; 2♀: Idem, x.2000; 1♀, 1♂: Idem, xi.2000; 2♀: Idem, xii.2000; 1♀: Idem, 21–25.viii.2001; 10♀: Idem, 16–20.viii.2001; 2♀: Idem, 27–31.vii.2001; 3♀: Idem, 1–5.ix.2001; 1♀: Idem, vi.2002; 9♀, 2♂: Idem, ix.2003; 37♀, 1♂: Idem, vii.–ix.2001; 1♀: Idem, viii.2000, A. v. Harten & A.R. Al Yarimi; 1♀: “Yemen (7269), Seyun, light trap, 4–6.ix.2002, A. van Harten, RMNH’03”; 1♀: Idem, 12–14.viii.2002; 3♀: Idem, vi.2002; 3♀: “Yemen (5404), Haman ‘Ali, from coffee berries (with *Ceratitis capitata*?), 14.iii.2001, A. van Harten, RMNH’02”; 1♀: “Yemen (6158), Al Lahima, Mal[aise] trap, 17.ix.–14.xi.2001, A. van Harten, RMNH’02”; 2♀, 1♂: “Yemen: Ta’izz (4056), light trap, viii.1999, A. van Harten & A. Ahwad, RMNH’00”; 1♀: Idem, viii.2000, A. v. Harten & A.R. Al Yarimi; 1♀: Idem, ix.2000; 1♀, “Yemen (7189), Al Kadan, light trap, v.2002, A. van Harten & A.R. Al Yarimi, RMNH’02”; 1♀: Idem, i.2003, A. v. Harten & T. Abdul-Haq; 3♀: Idem, x.2001. Excluded from type series: 9♀: “Yemen: Al Kowd (6151), 1–5.ix.2001, light trap, A. v. Harten & S. Al Haruri, RMNH’02”; 2♀: Idem, 16–20.viii.2001; 1♂: “United Arab Emirates, NARC near Sweihan (1473), light trap, 20–30.iv.2005, 24°24'N, 55°26'E, A. v. Harten, RMNH’05”.

**Figures 109–111.**
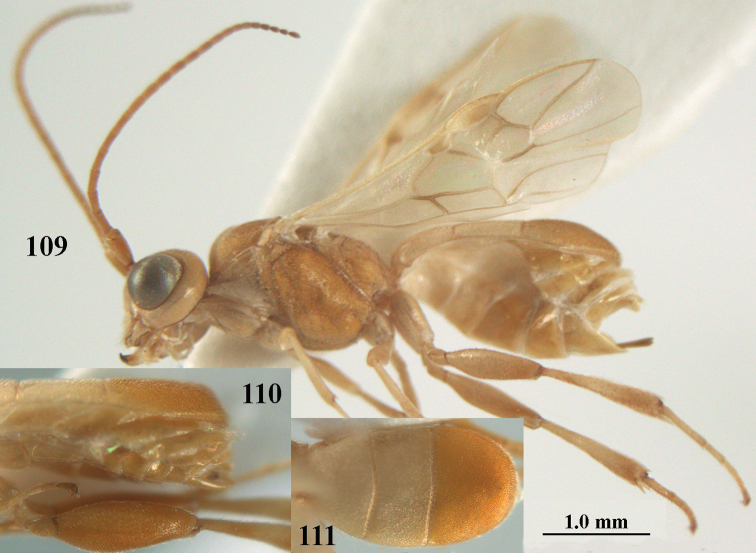
*Phanerotoma glabritemporalis* van Achterberg, sp. nov., ♀, holotype (but **110, 111** of ♂, paratype) **109** habitus lateral **110** metasoma and hind femur lateral **111** metasoma dorsal.

##### Diagnosis.

Ventral half of temple shiny, mostly smooth (at most punctulate) and convex (Fig. 121), vein 1-R1 of fore wing 3.0–3.6 × distance between apex of marginal cell and apex of wing, POL ca. 0.5 × diameter of posterior ocellus and third tergite ca. 1.5 × as long as second tergite; antenna of ♀ slender, subapical segment distinctly longer than wide and eighth segment from apex ca. 1.5 × as long as wide, without subapical protuberances; scutellar sulcus narrow and indistinctly crenulate anteriorly vein 1-M of fore wing much or slightly paler than vein 1-CU1; parastigma yellow, but sometimes somewhat infuscate posteriorly; convexity of T3 in lateral view variable; face mostly smooth (rarely finely sculptured) and very shiny; temple parallel-sided in lateral view, smooth (but sometimes finely sculptured) and shiny; clypeus flattened and approx. as wide as face; median carina of frons absent or obsolescent; differs from all other species by the shiny head combined with slender apical antennal segments of ♀ and a medium-sized third tergite with curved lateral borders (Fig. 114); males with strongly inflated hind femur (Fig. 110). The smooth mesosternum and inflated hind femur is shared by the Central Asian *P. minuta* Kokujev, 1903, but females of *P. glabritemporalis* have slenderer apical segments of antenna (robust in *P. minuta*), vein 1-R1 of fore wing 1.2–1.4 × as long as pterostigma (shorter than pterostigma), face mostly smooth (rugulose) and malar space 0.5 × as long as basal width of mandible (approx. equal). Excluded from the type series are specimens with the mesosternum less shiny as the ventral half of the temple and the latter is more or less coriaceous-rugulose; possibly it concerns a more sculptured variety of *P. glabritemporalis*.

**Figures 112–123.**
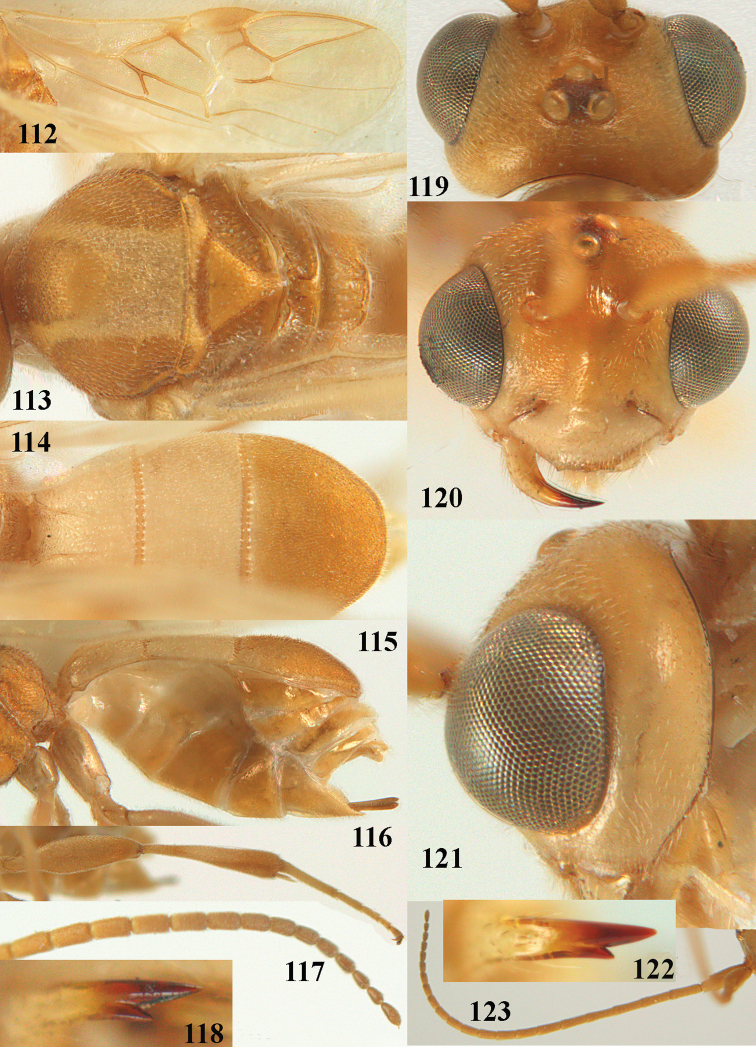
*Phanerotoma glabritemporalis* van Achterberg, sp. nov., ♀, holotype (but **122** of ♀ paratype) **112** fore wing **113** mesosoma dorsal **114** first–third metasomal tergites dorsal **115** metasoma lateral **116** hind leg lateral **117** apical half of antenna lateral **118** mandible ventral **119** head dorsal **120** head anterior **121** head lateral **122** mandible ventral **123** antenna lateral.

##### Description.

Female, holotype, length of body (excluding ovipositor) 5.1 mm; antenna 4.1 mm; fore wing 3.7 mm; visible part of ovipositor sheath 0.6 mm (only apex setose).

***Head*.** Width 1.4 × median length in anterior view and part of head above eye in lateral view 0.4 × height of eye (Fig. 121); antenna with 23 segments and 1.1 × longer than fore wing, segments slender and gradually shortened, narrowed apically and apical segments non-moniliform and narrowed basally (Fig. 117), third, fourth and penultimate segments 3.2, 3.0 and 1.8 × longer than wide in lateral view, respectively; area of stemmaticum rugulose-coriaceous; OOL: diameter of posterior ocellus: POL = 19: 10: 5; length of eye 2.4 × temple in dorsal view (Fig. 119); frons medially mostly smooth and very shiny, without median carina and laterally coriaceous-rugulose; vertex superficially rugulose, but posteriorly mostly smooth, rather shiny; temple parallel-sided in lateral view, mostly smooth and shiny, convex; face medially and clypeus mostly smooth and shiny; face without distinct median ridge and laterally rugulose, but smooth near eye; clypeus with three obsolescent teeth medio-ventrally (Fig. 120); eye large, strongly convex and in lateral view 1.9 × (measured medially) temple (Fig. 121), in anterior view 0.9 × minimum width of face; upper condyle of mandible near lower level of eyes (Fig. 120); malar space aciculate, shiny and 0.5 × as basal width of mandible; lower tooth of mandible 0.3 × as long as apical tooth (Fig. 118).

***Mesosoma*** (Figs 109, 113). Length 1.5 × its width in lateral view; side of pronotum mainly rugose, but dorsally mostly smooth; mesosternum smooth and very shiny; mesoscutum coriaceous and rather dull, but notaulic courses and medio-posteriorly rugose and rather shiny, densely setose; notauli hardly indicated; scutellar sulcus wide and with ten carinae (Fig. 113); scutellum triangular, largely punctate and rather shiny; metanotum with short median carina anteriorly and truncate posteriorly; propodeum coarsely reticulate-rugose, anteriorly with some longitudinal carinae, without distinct median and transverse carinae, latero-posteriorly not tuberculate. ***Wings*.** Fore wing 2.7 × longer than its maximum width; length of 1-R1 1.5 × as long as swollen pterostigma; 1-R1 3.1 × distance between apex of marginal cell and apex of wing; r issued far beyond middle of pterostigma and 0.5 × 3-SR; 2-SR curved and distally subparallel with posterior margin of pterostigma (Fig. 112); SR1 nearly straight; 2-SR+M rather long because of distinctly postfurcal m-cu; parastigma large; 1-CU1 0.4 × as long as vein 2-CU1; r:3-SR:SR1 = 9:20:130; 2-SR:3-SR:r-m = 26:20:11; r-m reclivous; 2-M weakly curved and horizontal (Fig. 112). Hind wing: M+CU:1-M:1r-m = 19:16:10. ***Legs*.** Hind femur 3.5 × as long as wide and widened subbasally; middle tibia with ivory blister; inner spur of middle tibia 0.6 × its basitarsus; hind coxa mostly smooth and shiny; hind tibia and basitarsus slender (Fig. 116).

***Metasoma*** (Figs 114, 115). Oval in dorsal view, 1.7 × as long as wide and 1.4 × as long as mesosoma; first and second tergites finely and densely longitudinally rugose; third tergite 1.5 × longer than second tergite and laterally curved, in lateral view rather convex, densely rugulose and medio-posteriorly truncate (Fig. 114), lateral lamella narrow, not protruding latero-apically and medio-apically truncate; ovipositor sheath medium-sized, its visible part 0.12 × as long as fore wing and 0.18 × metasomal carapace and setose part 0.03 × fore wing; hypopygium apically with short triangle, without apical spine and with rather short setae (Fig. 115).

***Colour*.** Brownish yellow; palpi, mandible (except dark brown teeth), clypeus, malar space, temple ventrally, pronotum, propleuron, legs (but hind femur and tibia brownish apically), first and second metasomal tergites pale yellow or ivory; apical third of antenna, ovipositor sheath and stemmaticum brown; pterostigma pale brownish yellow but basally pale yellowish (Fig. 112); wing membrane subhyaline but below pterostigma slightly infuscate; parastigma, veins 1-M, 2-CU1 and m-cu of fore wing yellow and veins r, 1-CU1, cu-a, 2-SR, 3-SR and 2-M rather dark brown.

##### Male.

Similar to female but hind femur strongly inflated (Fig. 110) and vein 1-R1 of fore wing 1.0–1.2 × as long as pterostigma.

##### Variations.

Length of fore wing of both sexes (2.1–)2.9–3.9 mm; apical antennal segments of ♀ usually non-moniliform, but especially in specimens from Yemen often submoniliform or rarely moniliform; third tergite 1.5–1.7 × longer than second tergite; medially frons very shiny and smooth but sometimes somewhat aciculate dorsally; vein 1-R1 of fore wing of ♀ 1.2–1.4 × as long as pterostigma; sometimes pterostigma darkened but anteriorly pale yellowish; stemmaticum brown or black; vein 1-M of fore wing varies from pale yellow (typical) to brown or dark brown; excluded specimens have face and temple finely sculptured.

##### Biology.

Unknown.

##### Distribution.

United Arabian Emirates, Yemen.

##### Etymology.

Named after the shiny and smooth temples (*glabrus* is Latin for smooth).

#### Phanerotoma graciloides

Taxon classificationAnimaliaHymenopteraBraconidae

van Achterberg, 1990

F2AA5D74-7811-57C9-BAAA-99389ABE1BE8

Figs 124–126 , 127–137

Phanerotoma (Bracotritoma) graciloides van Achterberg, 1990: 37–38.

##### Type material.

***Holotype***, ♀ (NHMUK): “**Saudi Arabia**, W. Büttiker”, “Rumah, 9.xi.1979”. ***Paratypes***, 2 ♀ (NHMUK, RMNH): topotypic, same date; 2♂, (NHMUK, RMNH): “Saudi Arabien, W. Büttiker”, “Bahara, 24.8.76”; 1♀, (NHMUK): Idem, but without date and locality.

##### Additional material.

From **United Arab Emirates** (Wadi Safad; Wadi Wurajah Farms; al-Ajban; NARC near Sweihan; Sharjah Desert Park; Sharjah; Sharjah x Khor Kaiba; Hatta; Fujairah) and **Yemen** (Al Kowd; Ta’izz; Mayfa’ah; Hamman’Ali; Al Kadan; Ar Rujum; Al Mukalla).

##### Diagnosis.

Antenna of ♀ near apical 0.4 widened; penultimate antennal segments of ♀ somewhat less robust than of *P. permixtellae* (Fig. 132); length of eye of ♀ in dorsal view 2.2–3.0 × temple (Fig. 133); ocelli small; inner tooth ca. 0.8 × apical tooth of mandible (Fig. 137); vein r of fore wing slightly reclivous and 1.0–1.5 × vein 3-SR (Fig. 127); vein 1-R1 of fore wing approx. as long as pterostigma (Fig. 127) or longer; pterostigma rather slender and largely dark brown except basally; parastigma dark brown or brown, darker than yellowish vein 1-M, rarely both yellowish; third metasomal tergite rather shiny, often superficially sculptured or smooth, flattened medially and distinctly acute posteriorly in lateral view (Fig. 130); propodeum more sparsely and rather irregularly reticulate, with rather coarse transverse carina or rugae more or less developed; hypopygium of ♀ with short up curved triangular apex (Fig. 130); hind femur of male strongly inflated (Fig. 125); length of fore wing 1.2–2.2 mm (usually 1.3–1.6 mm).

**Figures 124–126.**
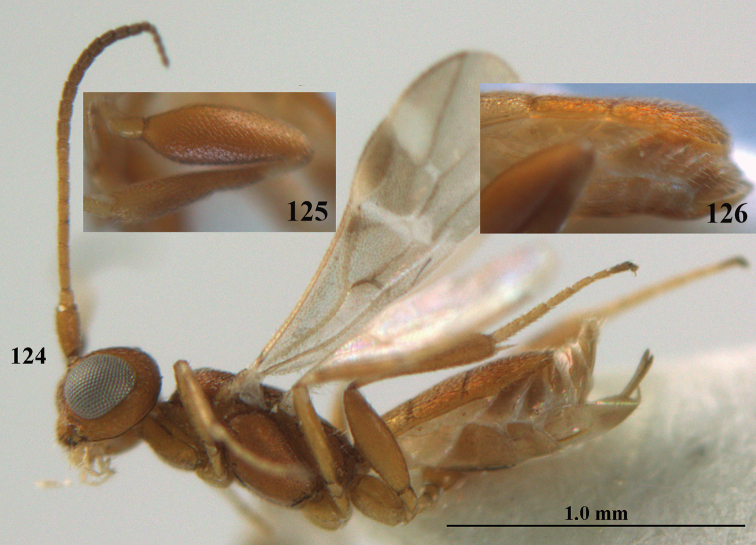
*Phanerotoma graciloides* van Achterberg, ♀ (but **125, 126** of ♂), Yemen **124** habitus lateral **125** hind femur lateral **126** metasoma, lateral.

##### Distribution.

Saudi Arabia, *United Arab Emirates, *Yemen.

##### Biology.

Unknown.

**Figures 127–137.**
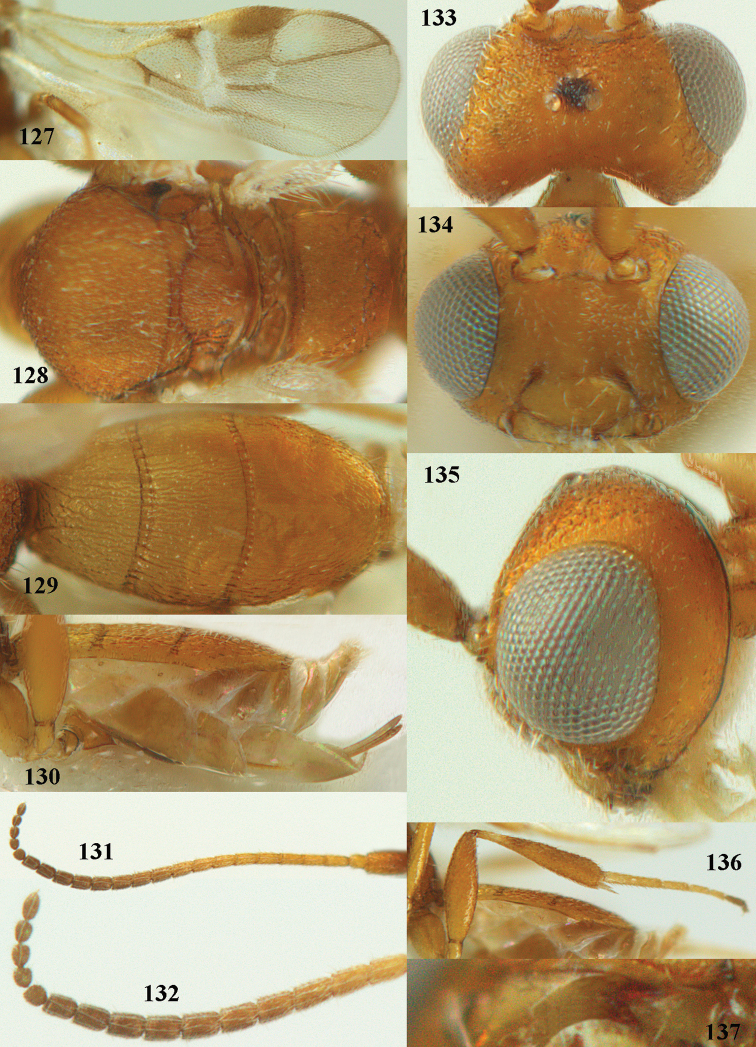
*Phanerotoma graciloides* van Achterberg, ♀, Yemen **127** fore wing **128** mesosoma dorsal **129** first–third metasomal tergites dorsal **130** metasoma lateral **131** antenna lateral **132** apical half of antenna lateral **133** head dorsal **134** head anterior **135** head, lateral **136** hind leg lateral **137** mandible ventral.

#### Phanerotoma granulata sp. nov.

Taxon classificationAnimaliaHymenopteraBraconidae

652F3F44-C9D7-5C37-9710-FD42F7CC9FD6

http://zoobank.org/6F4AC5DC-9AC6-403F-BFFF-3370ACC413BA

Figs 138–140 , 141–151

##### Type material.

***Holotype***, ♀ (RMNH), “**Yemen**: Al Kowd (3901), vii.1999, light trap, A. v. Harten & S. Al Haruri, RMNH’00”. ***Paratypes***: 10♀: same data as holotype; 8♀: Idem, viii.1999; 9♀: Idem, ix.1999; 1♀: Idem, xii.1999; 2♀: Idem, i.2000; 5♀: Idem, ii.2000; 1♀: Idem, iii.2000; 7♀: Idem, iv.2000; 47♀, 2♂: Idem, v.–vi.2000; 19♀: Idem, vii. 2000; 76♀, 5♂: Idem, viii.2000; 2♀, 1♂: Idem, xii.2000; 1♀: Idem, x.2000; 1♀: Idem, iii.2001; 22♀, 1♂: idem, iv.2001; 54♀, 3♂: idem, 8–12.vii.2001; 21♀: idem, 17–21.vii.2001; 4♀: Idem, viii.2001; 6♀: Idem, vii.–ix.2001; 2♀: Idem, 6–10.viii.2001; 33♀: Idem, 16–20.viii.2001; 76♀, 4♂: Idem, 21–28.viii.2001; 50♀, 2♂: Idem, 1–5.ix.2001; 61♀, 2♂: Idem, vi.2002; 60♀, 4♂: Idem, 27–31.vii.2002; 5♀, 2♂: Idem, i.–iii.2003; 5♀: Idem, v.2003; 92♀, 7♂: Idem, ix.2003; 1♀, “Yemen: Ta’izz (3066), light trap, 25–28.iv.1998, A. van Harten & Ahmad Ahwad, RMNH’99”; 1♀: Idem, vii.2002; 2♀: Idem, v.2000; 1♀: Idem, viii.2000; 2♀: Idem, viii.1999; 8♀, “Yemen: Al Kadan (7189), v.2002, light trap; A. v. Harten & A.R. Al Yarimi, RMNH’02”; 3♀: Idem, iv.2002; 356♀, 12♂: Idem, i.2003; 1♀: Idem, x.2001, A. v. Harten & T. Abdul-Haq; 3♀: Idem, ix.2001; 1♂: Idem, x.2001; 1♀: Idem, xi.2001; 2♀, 1♂: Idem, 16.ii.–31.iii.1998; 2♀: Yemen (7009), Lahj, viii.2002, Mal[aise] trap, A. v. Harten & A. Sallum, RMNH’02”; 2♀, “Yemen: Ar Rujum (5700), 9.iv.–5.vi.2001, Mal. trap, A. v. Harten, RMNH’02”; 1♀: “Yemen, Seyun, light trap, 12–14.viii.2002, A. van Harten, RMNH’03”; 39♀, 1♂: “Yemen: Hamman ‘Ali (5404), from coffee-berries, 14.iii.2001, A. v. Harten, RMNH’02”; 1♀: “Yemen (7589), Al Mukalla, light traps, i.–ii.2003, A. van Harten, RMNH’03”; 1♀, 1♂: “**United Arab Emirates**, al-Ajban (2663), light trap, 17.x.–9.xi.2005, 24°36'N, 55°01'E, A. v. Harten, RMNH’06”; 1♀: Idem, 27.v.–26.vi.2006; 1♀, “United Arab Emirates, NARC near Sweihan, 24°24'N, 55°26'E, 20–30.iv.2005 (1473), light trap, A. v. Harten, RMNH’05”.

**Figures 138–140.**
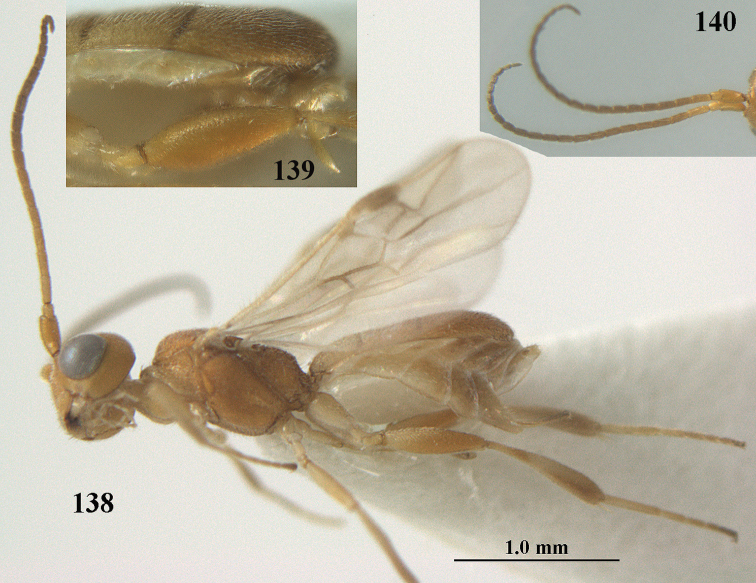
*Phanerotoma granulata* van Achterberg, sp. nov.,♀, holotype (but **139, 140** of ♂, paratype) **138** habitus lateral **139** metasoma and hind femur lateral **140** antennae lateral.

##### Diagnosis.

**Figures 141–151.**
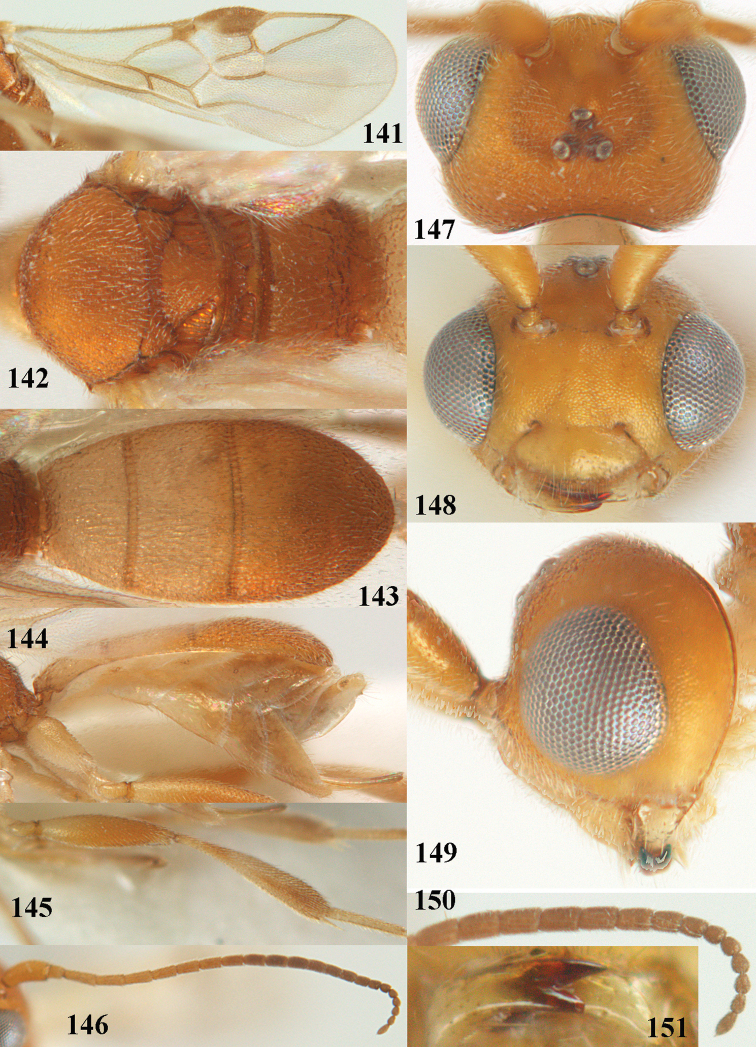
*Phanerotoma granulata* van Achterberg, sp. nov., ♀, holotype **141** fore wing **142** mesosoma dorsal **143** first–third metasomal tergites dorsal **144** metasoma lateral **145** hind leg lateral **146** antenna lateral **147** head dorsal **148** head anterior **149** head lateral **150** apical half of antenna lateral **151** mandible ventral.

Ocelli comparatively small; upper condyles of mandibles near lower level of eyes; antenna of ♀ with ca. six moniliform apical segments (Fig. 150); inner tooth of mandible 0.6–0.8 × as long as apical tooth (Fig. 151); parastigma large and yellow; vein r of fore wing 1.2–2.3 × vein 3-SR of fore wing (Fig. 141); third tergite more or less obtuse apically in lateral view or with transverse depression, finely sculptured and matt medially, comparatively short; area of mesosternum near mesosternal sulcus rather matt and distinctly granulate (Fig. 138); temple narrowed ventrally; hind femur of ♂ moderately inflated (Fig. 139). Very similar to the Central Asian and East Mediterranean *P. parva* Kokujev, 1903, but *P. granulata* has mesosternum distinctly granulate and rather matt (shiny and superficially granulate in *P. parva*), hind femur of ♂ moderately inflated (strongly inflated), vein 1-M of fore wing pale yellow (dark brown), vein 1-R1 of fore wing approx. as long as pterostigma (distinctly shorter), marginal cell of fore wing rather slender (wider), parastigma of fore wing wide (rather small) and apical antennal segments of ♀ moniliform (non-moniliform).

##### Description.

Female, holotype, length of body (excluding ovipositor) 2.8 mm; antenna 2.0 mm; fore wing 2.2 mm; visible part of ovipositor sheath 0.4 mm (only apex with few setae).

***Head*.** Width 1.5 × median length in anterior view and part of head above eye in lateral view 0.3 × height of eye (Fig. 149); antenna with 23 segments and as long as fore wing, near its apical third segments elongate and longer than wide, narrowed apically and six apical segments moniliform (Figs 146, 150) and apical segment with minute spine, third, fourth and penultimate segments 3.0, 2.5 and 1.5 × longer than wide in lateral view, respectively; area of stemmaticum granulate; OOL: diameter of posterior ocellus: POL = 20: 6: 7; length of eye 1.9 × temple in dorsal view (Fig. 147); frons granulate and matt laterally, superficially granulate medially and rather shiny, without median carina; vertex granulate, posterior rugulose-granulate and matt; temple granulate and matt; face granulate and with small median bump dorsally but no distinct median carina; clypeus smooth, shiny and distinctly narrower than face and with three minute teeth medio-ventrally (Fig. 148); eye large, strongly convex and in lateral view 1.3 × (measured medially) as wide as temple (Fig. 149), in anterior view its height 0.8 × minimum width of face; upper condyle of mandible near lower level of eyes (Fig. 148); malar space granulate, with satin sheen and 0.8 × as long as basal width of mandible; lower tooth of mandible 0.7 × as long as apical tooth (Fig. 151).

***Mesosoma*** (Figs 138, 142). Length 1.6 × its width in lateral view; side of pronotum largely coarsely rugose; propleuron posteriorly evenly convex; mesosternum densely granulate and rather matt; mesoscutum densely granulate, but medio-posteriorly rugulose-granulate; scutellum flat, distinctly granulate and rather matt; notauli not indicated; scutellar sulcus narrow and with nine carinae (Fig. 142); metanotum with short median carina anteriorly and no tooth posteriorly; propodeum coarsely reticulate-rugose, without distinct median and transverse carinae, and latero-posteriorly not tuberculate. ***Wings*.** Fore wing 2.8 × longer than its maximum width; length of 1-R1 as long as pterostigma; distance between wing apex and vein 1-R1 0.7 × length of vein 1-R1; r issued far beyond middle of pterostigma and 1.8 × 3-SR; 2-SR nearly straight and distally slightly converging to posterior margin of pterostigma (Fig. 141); SR1 nearly straight; 2-SR+M present, m-cu postfurcal; parastigma rather large; first discal cell of fore wing higher than first subdiscal cell; 1-CU1 0.5 × as long as vein 2-CU1; r:3-SR:SR1 = 7:4:39; 2-SR:3-SR:r-m = 21:4:8; r-m vertical; 2-M slightly curved (Fig. 141). Hind wing: M+CU:1-M:1r-m = 27:19:10. ***Legs*.** Hind femur matt, 4.1 × as long as wide and slightly widened submedially; middle tibia with weak ivory blister; inner spur of middle tibia 0.4 × its basitarsus; hind tibia slender (Fig. 145); hind coxa largely superficially coriaceous and with satin sheen.

***Metasoma*** (Figs 143, 144). Elliptical in dorsal view, 1.9 × as long as wide and 1.4 × as long as mesosoma; first and second tergites densely and finely longitudinally rugose; metasomal sutures medium-sized; third tergite distinctly convex medially, 1.5 × longer than second tergite and laterally curved, in lateral view rather convex, largely densely rugulose and with satin sheen (Fig. 144), lateral lamella narrow laterally, not protruding latero-apically and medio-apically truncate and medium-sized and area above it concave; ovipositor sheath narrow (Fig. 144), its visible part 0.2 × as long as fore wing and 0.3 × metasomal carapace and only its apex with few medium-sized setae; hypopygium apically with short robust triangle, without apical spine, and densely setose.

***Colour*.** Yellowish brown; apical half of antenna and stemmaticum more or less darkened; ovipositor sheath (but paler basally), veins 1-CU1, 2-CU1, r, 2-SR and 2-M of fore wing brown; clypeus, mandible (except dark brown teeth), palpi, propleuron, pronotal side ventrally, tegulum and humeral plate, legs (but hind femur rather brownish except basally and hind tibia subbasally and apically slightly darkened), first and second tergites and metasoma ventrally pale yellow; pterostigma (but basally partly pale yellowish) dark brown; parastigma, veins 1-M and m-cu of fore wing pale yellow; wing membrane basally and marginal cell subhyaline, remainder of apical half of fore wing slightly brownish.

##### Male.

Similar to female, but hind femur rather inflated (Fig. 139) and antennal segments slender and elongate (Fig. 140).

##### Variations.

Length of fore wing of ♀ 1.5–2.5 mm, of ♂ 1.9–2.2 mm; inner tooth of mandible robust and 0.6–0.8 × as long as apical tooth.

##### Distribution.

United Arab Emirates, Yemen.

##### Biology.

Unknown.

##### Etymology.

Named after the granulate mesosternum (*granum* in Latin for seed, small kernel).

#### Phanerotoma hellyeri sp. nov.

Taxon classificationAnimaliaHymenopteraBraconidae

8BF85597-960F-5955-9C2F-D2442E216CE1

http://zoobank.org/A84BE796-CCB6-4FDB-8BE7-A46C5DD97503

Figs 152–155 , 156–166

##### Type material.

***Holotype***, ♀ (RMNH), “**United Arab Emirates**, Sharjah Desert Park (2517), light trap, 20.x.–8.xi.2005, 25°17'N, 55°42'E, A. v. Harten, RMNH’06”. ***Paratypes***: 2♀, 3♂: Same data as holotype; 3♀: Idem, 29.iii.–6.iv.2005; 2♀, 1♂: Idem, 6–13.iv.2005; 1♀, 2♂: Idem, 13–23.iv.2005; 1♀: Idem, 25.ii.–25.iii.2006; 1♀: Idem, 10.xi.2004; 1♀: Idem, 30.iv.–7.v.2005; 2♀: Idem, 23–30.iv.2005; 1♀: Idem, 21–29.iii.2005; 1♀: Idem, 1.ii.–14.iii.2005; 2♀, 1♂: “United Arab Emirates, SSW of ad-Dhaid (6154), light tr[ap], 24–30.v.2006, 25°09'N, 55°48'E, A. v. Harten, RMNH’06”; 2♀: “United Arab Emirates, NARC near Sweihan (1410), light trap, 1.ii.–14.iii.2005, 24°24'N, 55°26'E, A. v. Harten, RMNH’05”; 6♀, 2♂: Idem, 28.iii.–2.iv.2005; 24♀, 2♂: Idem, 9–20.iv.2005; 22♀, 3♂: Idem, 20–30.iv.2005; 1♂: Idem, 14–28.iii.2005; 1♀: “United Arab Emirates, al-Ajban (6426), Malaise tr[ap], 25.v.–26.vi.2006, 24°36'N, 55°01'E, A. v. Harten, RMNH’07”; 3♀, 1♂: Idem, 22.x.–9.xi.2005; 3♀, 3♂: Idem, 7–28.xii.2006; 6♀: Idem, 17.iv.–29.v.2006; 3♀, 1♂: Idem, 27.v.–26.vi.2006; 1♂: Idem, 17.x.–9.xi.2005; 1♂: Idem, 12–19.vi.2006; 2♂: “United Arab Emirates, Fujairah (1314), light tr[ap], 13–19.iv.2005, 25°08'N, 56°21'E, A. v. Harten, RMNH’05”; 1♂: Idem, 2–13.v.2005; 6♂: Idem, 2.v.–5.vi.2005; 1♀, 1♂: Idem, 19.iv.–2.v.2005; ”; 1♀: Idem, 5–24.iii.2005; 2♀, 2♂: “United Arab Emirates, Sharjah (2279), light trap, 30.vi.–21.vii.2005, 25°17'N, 55°42'E, A. v. Harten, RMNH’06”; 1♂: “United Arab Emirates, Sharjah x Khor Kalba (6308–6311), light trap, [24°59'N, 56°09'E,] 31.v.–7.vi.2006, A. v. Harten, RMNH’06”; 1♂: Idem, 16–31.i.2006; 1♂: “United Arab Emirates, Hatta (6398), at light, 17–24.viii.2006, 24°49'N, 56°07'E, A. v. Harten, RMNH’06”; 2♀: Idem, 21.vi.–19.vii.2006; 1♀: “**Yemen** (no. 3111), Ta’izz, light trap, 26–28.v.1998, A. van Harten & Ahmad Ahwad, RMNH’98”; 1♀: Idem, ix.2000; 1♀: Idem, vii.2002; 1♀: “Yemen (6141), Al Kowd, light trap, 16–20.viii.2001, A. van Harten & S. Al Haruri, RMNH’02”; 1♀: Idem, 27–31.vii.2001; 1♀: “Yemen (7501), Al Kadan, light trap, i.2003, A. v. Harten & T. Abdul-Haq, RMNH’03”; 2♀: Idem, v.2002; 1♀: Idem, x.2001; 1♀: “Yemen (6158), Al Lahima, 17.ix.–14.xi.2001, Mal[aise] trap, A. v. Harten, RMNH’02”.

**Figures 152–155.**
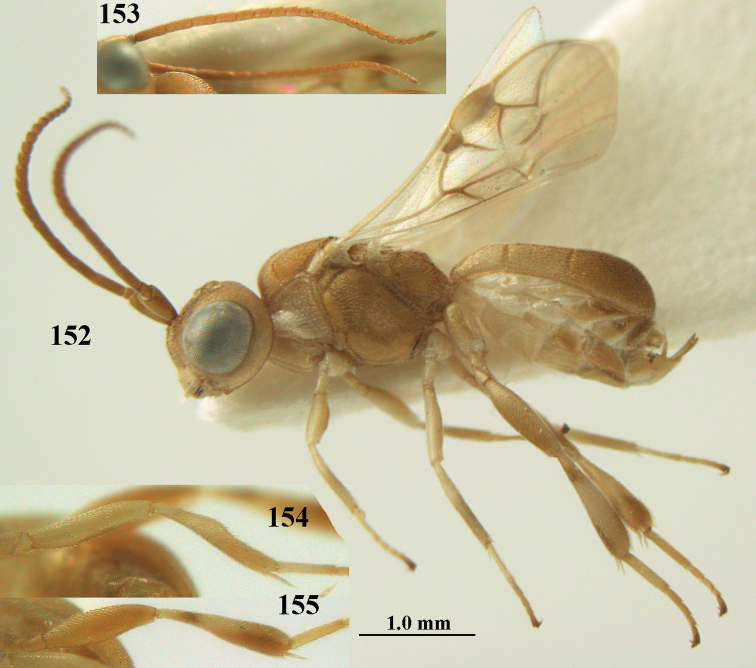
*Phanerotoma hellyeri* van Achterberg, sp. nov., ♀, holotype (but **153–155** of ♂, paratype) **152** habitus lateral **153** antenna **154** middle femur and tibia lateral **155** hind femur and tibia lateral.

##### Diagnosis.

Robust species with twelfth–16^th^ (counted from apex of antenna) antennal segments of ♀ widened (compared to more basal segments) and ventrally flattened, 13^th^ segment from apex of antenna of ♀ as long as wide (Figs 165, 166) and eighth–tenth apical segments moniliform, stocky, matt or slightly shiny (Fig. 166); stemmaticum yellow, more or less infuscate subapically; eye 2.0–2.1 × as wide as median width of temple in lateral view (Fig. 164); POL of ♀ slightly less than width of posterior ocellus; frons with median carina anteriorly; second submarginal cell somewhat longer than in *P. mesocellata*; parastigma usually largely yellow, rarely infuscate; vein 1-M (as usually parastigma) slightly darker than yellow M+CU1 of fore wing; blister of middle tibia medium-sized; hind femur and tibia rather stout (Fig. 160).

**Figures 156–166.**
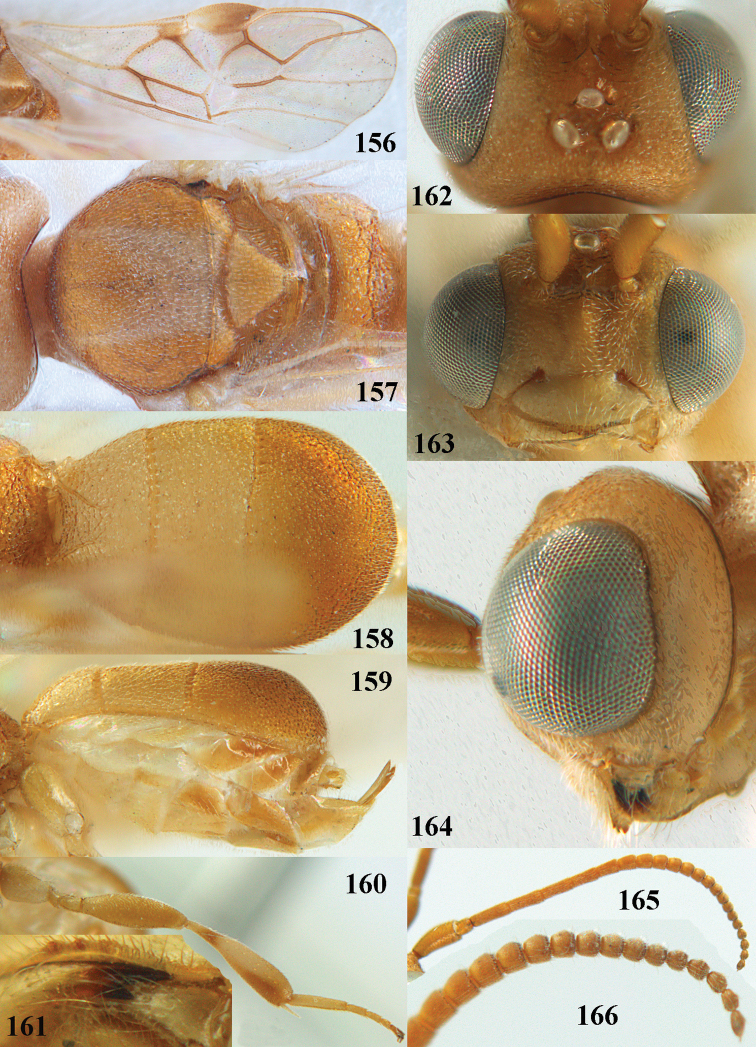
*Phanerotoma hellyeri* van Achterberg, sp. nov., ♀, holotype **156** fore wing **157** mesosoma dorsal **158** first–third metasomal tergites dorsal **159** metasoma lateral **160** hind leg lateral **161** mandible ventral **162** head dorsal **163** head anterior **164** head lateral **165** antenna lateral **166** apical half of antenna lateral.

##### Description.

Female, holotype, length of body (excluding ovipositor) 4.2 mm; antenna 3.0 mm; fore wing 3.1 mm; visible part of ovipositor sheath 0.3 mm (0.1 mm erect setose).

***Head*.** Width 1.6 × median length in anterior view and part of head above eye in lateral view 0.2 × height of eye (Fig. 164); antenna with 23 cylindrical segments and as long as fore wing, 10 apical antennal segments small, rather serrate and moniliform (Figs 165, 166), with short bristles apically and apical segment with rather long spine, third, fourth and penultimate segments 3.0, 2.6 and 1.3 × longer than wide in lateral view, respectively; area of stemmaticum rugulose; OOL: diameter of posterior ocellus: POL = 20: 10: 9; length of eye 3.8 × temple in dorsal view (Fig. 162); frons with median carina (V-shaped dorsally: Fig. 162), smooth antero-medially, posteriorly with curved rugae and finely rugose laterally; vertex rugose and with satin sheen, but posteriorly transversely rugulose; temple rugose but coriaceous near eye, convex, parallel-sided in lateral view and with satin sheen, directly narrowed behind eyes; face transversely rugose laterally, rugulose and with obsolescent median bump and rather shiny; clypeus smooth (except punctulation), shiny and as wide as minimum width of face, intertentorial distance 3.8 × minimum width between clypeus and eye (Fig. 163), long erect setose and with 3 distinct blunt teeth medio-ventrally (Fig. 161); eye large, strongly convex and in lateral view twice wider than temple (measured medially; Fig. 164), in anterior view its height 0.9 × minimum width of face (Fig. 163); upper condyle of mandible above lower level of eyes (Fig. 163); malar space rugulose, with satin sheen and 0.5 × as long as basal width of mandible; lower tooth of mandible half as long as apical tooth, robust (Fig. 161).

***Mesosoma*** (Figs 152, 157). Length1.4 × its width in lateral view; side of pronotum reticulate-punctate; posteriorly propleuron bulging near central groove; mesosternum smooth and shiny; mesoscutum densely reticulate-rugose, with satin sheen, notauli anteriorly impressed; scutellum flat, finely punctate-rugose; scutellar sulcus medium-sized, with twelve carinae (Fig. 157); metanotum with median carina and medio-posterior tooth, its posterior border finely serrate; propodeum coarsely reticulate-rugose, dorsal face short, without transverse or median carinae, and latero-posteriorly weakly tuberculate. ***Wings*.** Fore wing 2.7 × longer than its maximum width; 1-R1 1.2 × as long as pterostigma; distance between wing apex and marginal cell apex 0.25 × length of 1-R1; r issued far beyond middle of pterostigma and 0.2 × 3-SR; 2-SR distinctly curved and subparallel with posterior margin of pterostigma (Fig. 156); SR1 curved; m-cu interstitial; parastigma large; 1-CU1 0.3 × as long as vein 2-CU1, cu-a 1.3 × 1-CU1, strongly inclivous; r:3-SR:SR1 = 5:22:51; 2-SR:3-SR:r-m = 27:22:7; r-m reclivous; 2-M oblique and slightly curved (Fig. 156). Hind wing: M+CU:1-M:1r-m = 21:19:10. ***Legs*.** Hind femur matt, 3.2 × as long as wide and robust; hind tibia swollen (Fig. 161); middle tibia with distinct ivory blister; inner spur of middle tibia 0.5 × its basitarsus; hind coxa mostly smooth, but dorsally partly superficially granulate and rather shiny.

***Metasoma*** (Figs 158, 159). Oval in dorsal view, 1.5 × as long as wide and 1.1 × as long as mesosoma; first and second tergites densely and coarsely longitudinally rugose; second metasomal suture rather wide and slightly curved; third tergite 1.6 × longer than second tergite and laterally curved, convex medially, rounded posteriorly in dorsal view (Fig. 158), obtuse posteriorly in lateral view (Fig. 159), densely reticulate-rugose and with satin sheen (Fig. 158), lateral lamella narrow, wide latero-apically and medio-apically; ovipositor sheath narrow, apically somewhat widened and darkened (Fig. 159), its visible and setose part 0.1 × as long as fore wing and 0.2 × metasomal carapace, and with erect setae; hypopygium of ♀ with short widely triangular and up curved apical protuberance (Fig. 159) and with short setae.

***Colour*.** Pale brownish yellow (including stemmaticum); apex of antenna, hind tibia apically and subbasally and apex of ovipositor sheath rather brown; parastigma and vein 1-M yellow; clypeus, palpi, tegulae, remainder of legs, mesoscutum medio-posteriorly, first and second tergites and metasoma baso-ventrally pale yellowish or ivory; pterostigma (but basally and apically pale yellowish) and most veins brown; wing membrane slightly brownish below pterostigma.

##### Male.

Similar to female (including hind femur and tibia: Fig. 155), but antenna slenderer (Fig. 153).

##### Biology.

Unknown.

##### Variations.

Length of fore wing of ♀ 2.4–3.7 mm, of ♂ 2.2–3.2 mm; vein 2-SR usually evenly curved, but sometimes distinctly bent and parallel with posterior margin of pterostigma; vein 1-M of fore wing and parastigma pale yellowish, but sometimes more or less brown; pterostigma partly dark brown, largely or entirely pale yellowish; third tergite brown to rather dark brown; stemmaticum brownish yellow, only rarely darkened; apical half of antenna brownish yellow or dark brown.

##### Distribution.

United Arab Emirates, Yemen.

##### Etymology.

The new species is named after Peter Hellyer for his life-long research on the archaeology and ecology of the United Arab Emirates and his support of the series “Arthropod Fauna of the UAE”.

#### Phanerotoma latifemorata sp. nov.

Taxon classificationAnimaliaHymenopteraBraconidae

E038E771-1A01-5FF8-AA1A-B1AEC1210AEB

http://zoobank.org/F304D34B-FCA2-4295-928B-03001DD81D08

Figs 167 , 168–178

##### Type material.

***Holotype***, ♀ (RMNH), “**Yemen**, Ta’izz, no. 4932, light tr[ap], viii.2000, A. v. Harten & A.R. Al Yarimi, RMNH’01”. ***Paratypes***: 2♀: Idem, 3–24.i.1999; 1♀: Idem, 5.i.–2.ii.1998.

**Figure 167.**
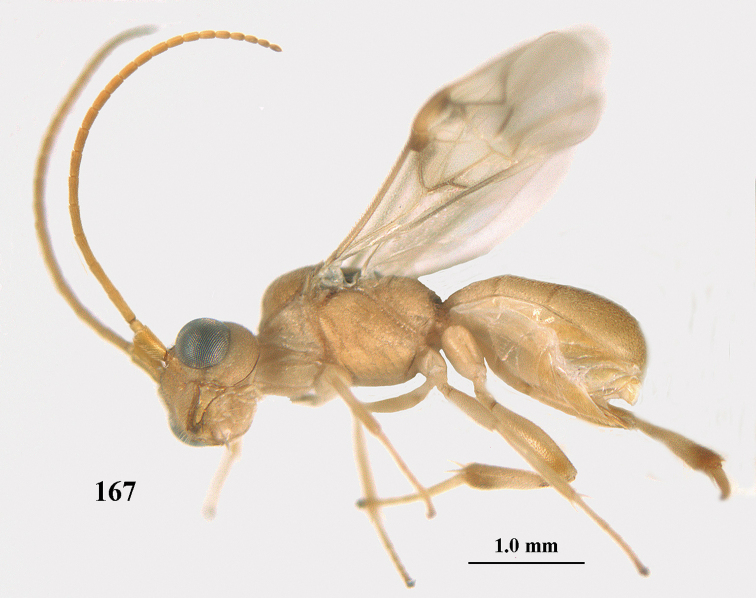
*Phanerotoma latifemorata* van Achterberg, sp. nov., ♀, holotype, habitus, lateral.

##### Diagnosis.

**Figures 168–178.**
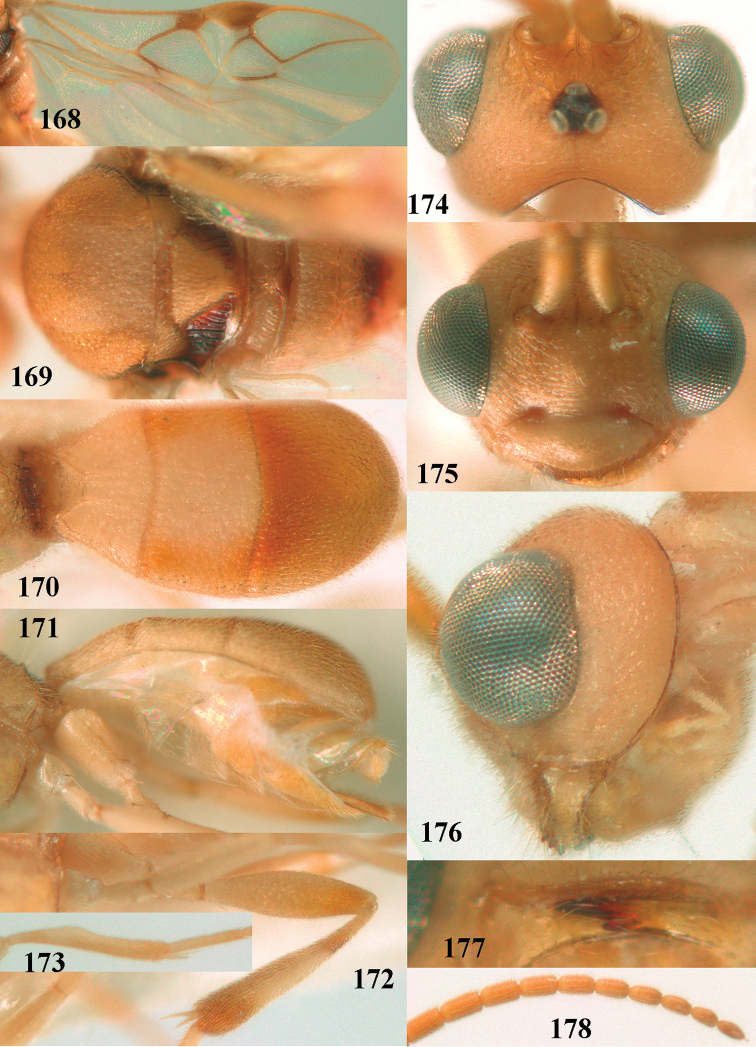
*Phanerotoma latifemorata* van Achterberg, sp. nov., ♀, holotype **168** wings **169** mesosoma dorsal **170** first–third metasomal tergites dorsal **171** metasoma lateral **172** hind leg lateral **173** middle tibia lateral **174** head dorsal **175** head anterior **176** head lateral **177** mandible ventral **178** apical third of antenna lateral.

Hypopygium of ♀ straight apically (Figs 167, 171), without up curved apical triangle or spine-like protuberance; apical quarter of ♀ antenna more or less serrate in lateral view because of small subapical protuberances on segments, sixth segment subapically narrowed (Fig. 178); intertentorial distance of clypeus ca. 3 × minimum distance between clypeus and eye, clypeus 0.9 × wider than face and rather shiny (Fig. 175); inner tooth of mandible medium-sized (Fig. 177); propleuron transversely rugose; first discal cell of fore wing very wide anteriorly (Fig. 168); vein r of fore wing 0.2 × vein 3-SR and angled with vein 3-SR (Fig. 168); vein 2-SR of fore wing curved; vein cu-a of fore wing 0.8–0.9 × vein 1-CU1 and moderately oblique (Fig. 168); middle tibia very slender (Fig. 167); hind femur and tibia of ♀ and tarsal claws robust (Figs 167, 172). Similar to *P. ocularis* because of size of ocelli and anteriorly wide first discal cell of fore wing, but with slender middle tibia, straight apex of hypopygium, rugose propleuron and elongate apical antennal segments in female.

##### Description.

Female, holotype, length of body (excluding ovipositor) 4.3 mm; antenna 4.3 mm; fore wing 3.6 mm; visible part of ovipositor sheath 0.3 mm (erect setae mainly at apex).

***Head*.** Width 1.6 × median length in anterior view and part of head above eye in lateral view 0.3 × height of eye (Fig. 176); antenna with 23 segments and 1.2 × longer than fore wing, segments slender and gradually shortened, segments of apical quarter with minute subapical protuberances and widened subapically (Figs 167, 178), without moniliform apical segments (Fig. 178), third, fourth and penultimate segments 3.4, 3.2 and 2.0 × longer than wide in lateral view, respectively; area of stemmaticum superficially coriaceous; OOL: diameter of posterior ocellus: POL = 13: 5: 3; length of eye 1.9 × temple in dorsal view (Fig. 174); frons medially with some coarse rugae, shiny and with distinct median carina, laterally rugose; vertex rugose and rather shiny; temple rugose and with satin sheen, convex; face rather shiny and transversely rugose, with fine median carina dorsally; width of clypeus 0.9 × minimum width of face (intertentorial distance 3.1 × minimum distance between clypeus and eye ventrally), convex, mostly smooth and shiny (Fig. 175); clypeus with three distinct obtuse teeth medio-ventrally (Fig. 175); eye large, strongly convex and in lateral view 1.3 × (measured medially) wider than temple (Fig. 176), in anterior view its height 0.8 × minimum width of face; upper condyle of mandible below lower level of eyes (Fig. 175); malar space rugose, rather shiny and 0.4 × as basal width of mandible; lower tooth of mandible rather small and 0.4 × as long as apical tooth (Fig. 177).

***Mesosoma*** (Figs 167, 169). Length1.4 × its width in lateral view; side of pronotum largely rugose, but dorsally granulate; propleuron convex and transversely rugose, rather shiny; mesosternum granulate and matt; mesoscutum finely reticulate-rugose and rather shiny; notauli slightly indicated anteriorly; scutellar sulcus wide medially, with 6 carinae (Fig. 169); scutellum densely rugulose-granulate (smooth apically), convex and rather shiny; metanotum with short median carina anteriorly and with minor protrusion medio-posteriorly; propodeum coarsely reticulate-rugose on rugulose background, without distinct median and transverse carinae, latero-posteriorly not tuberculate. ***Wings*.** Fore wing 2.7 × longer than its maximum width; length of 1-R1 1.5 × as long as pterostigma; r issued rather far beyond middle of pterostigma and 0.2 × 3-SR; distance between 1-R1 and wing apex 0.4 × 1-R1; 2-SR distinctly bent and distally parallel with posterior margin of pterostigma (Fig. 168); SR1 curved; m-cu sub-interstitial; parastigma very large and first discal cell wide anteriorly (Fig. 168); 1-CU1 0.35 × as long as vein 2-CU1, cu-a distinctly inclivous and 0.9 × as long as 1-CU1; r:3-SR:SR1 = 5:22:57; 2-SR:3-SR:r-m = 28:22:8; r-m reclivous; 2-M oblique, weakly curved (Fig. 168). Hind wing: M+CU:1-M:1r-m = 19:17:10. ***Legs*.** Hind femur widened medially and 3.4 × as long as wide (Fig. 172); middle tibia slender and with small blister; inner spur of middle tibia 0.6 × its basitarsus; hind coxa superficially granulate and with satin sheen; hind tibia wide medially (Fig. 172); tarsal claws medium-sized.

***Metasoma*** (Figs 170, 171). Oval in dorsal view, 1.7 × as long as wide and 1.2 × as long as mesosoma; first and second tergites coarsely longitudinally rugose; third tergite 1.4 × longer than second tergite and laterally curved, convex, densely reticulate-rugose and medio-posteriorly truncate (Fig. 170), lateral lamella narrow, but wider latero-apically and medio-apically truncate; ovipositor sheath hardly widened apically (Fig. 171), its visible part 0.08 × as long as fore wing and 0.12 × metasomal carapace, its setae erect and mainly near apex of sheath; hypopygium apically acute, densely setose, without short up curved triangle or apical spine (Fig. 171).

***Colour*.** Pale brownish yellow (including tegulum, but humeral plate partly dark brown); palpi, mandible (except dark brown teeth), clypeus, malar space, prothorax, legs (but telotarsi and hind tibia apically and subbasally brownish), first and second metasomal tergites (except laterally) and basal half of metasoma ventrally whitish or ivory; ovipositor sheath apically brown; stemmaticum and scutellum laterally dark brown; parastigma largely, pterostigma medially dark brown, but basally and narrowly apically pale yellowish (Fig. 168); wing membrane subhyaline but below dark part of pterostigma slightly infuscate; parastigma and vein m-cu largely pale yellow; apical half of metasoma, veins 1-M, 1-CU1, base of 2-CU1, r, 2-SR basally, 2-M and 3-SR of fore wing dark brown.

##### Male.

Unknown.

##### Variations.

Length of fore wing 3.5–3.8 mm; vein cu-a of fore wing 0.8–0.9 × as long as vein 1-CU1.

##### Biology.

Unknown.

##### Distribution.

Yemen.

##### Etymology.

Named after the robust hind femur (*latus* is Latin for wide).

#### Phanerotoma lepta sp. nov.

Taxon classificationAnimaliaHymenopteraBraconidae

3BBBB6AB-E1C1-581D-A8BB-DB3D18FF3A40

http://zoobank.org/CDF514A0-BCBC-4EC4-8404-FEEDE8750A82

Figs 179–181 , 182–193

##### Type material.

***Holotype***, ♀ (RMNH), “**United Arab Emirates**, Sharjah Desert Park (1202), light tr[ap], 29.iii.–6.iv.2005, 25°17'N, 55°42'E, A. v. Harten, RMNH’06”. ***Paratypes***: 3♀, 3♂: Same data as holotype; 1♂: Idem, 9–21.iii.2005; 1♀, 2♂: Idem, 21–29.iii.2005; 3♀, 2♂: Idem, 6–13.iv.2005; 3♀, 3♂: Idem, 23–30.iv.2005; 3♂: Idem, 30.iv.–31.v.2005; 2♀, 3♂: Idem, 30.iv.–7.v.2005; 1♂: Idem, 25.v.–15.vii.2008; 2♀, 4♂: Idem, 13–23.iv.2005; 1♀: Idem, 25.i.–22.ii.2005; 1♀: Idem, 25.ii.–25.iii.2006; 1♀, 1♂: Idem, 20.x.–8.xi.2005; 1♀: Idem, 24.iii.–1.iv.2007; 1♂: “United Arab Emirates, Sharjah (1700–1706), light trap, 27.iv..–5.vi.2005, 25°17'N, 55°42'E, A. v. Harten, RMNH’05”; 8♀: Idem, 1–31.i.2005; 2♂: Idem, 1–10.ii.2005; 5♀: Idem, 30.vi.–21.vii.2005; 2♀, 7♂: Idem, 11–17.x.2004; 7♀, 18♂: “United Arab Emirates, Fujairah (1224), light tr[ap], 5–24.iii.2005, 25°08'N, 56°21'E, A. v. Harten, RMNH’06”; 6♀, 9♂: Idem, 24.iii.–6.iv.2005; 6♀, 11♂: Idem, 13–19.iv.2005; 9♀, 7♂: Idem, 2–13.v.2005; 3♀, 7♂, Idem, 19.iv.–2.v.2005; 16♀, 8♂: Idem, 2.v.–5.vi.2005; 2♂: Idem, 13–29.xi.2005; 3♀: Idem, 29.xi.2005–2.i.2006; 4♀, 12♂: Idem, 28.ii.–1.iv.2006; 3♀, 13♂: Idem, 24.ii.–5.iii.2005; 1♀, 4♂: Idem, 16–24.ii.2005; 1♀, 2♂: “United Arab Emirates, Wadi Safad (11296), light trap, 2–26.i.2006, 25°13'N, 56°19'E, A. v. Harten, RMNH’10”; 4♀, 1♂: Idem, 14–21.v.2006; 1♀, 3♂: Idem, 1–8.vii.2006; 2♂: Idem, 31.i–21.ii.2006; 1♀: Idem, 6–13.v.2008; 1♂: “United Arab Emirates, Wadi Maidaq (3808), at light, 21.xii.2005–2.ii.2006, 25°18'N, 56°07'E, A. v. Harten, RMNH’06”; 1♀, 1♂: Idem, 27.iv.–4.v.2006; 2♀, 1♂: “United Arab Emirates, Bithnah (3699), at light, 11.xii.2005–18.i.2006, 25°17'N, 55°42'E, A. v. Harten, RMNH’06”; 2♂: Idem, 30.xii.2005–2.ii.2006; 1♀: “United Arab Emirates, NARC near Sweihan (4387), light trap, 26.ii.–2.iv.2006, 24°24'N, 55°26'E, A. v. Harten, RMNH’06”; 5♂: Idem, 2–9.iv.2005; 6♀: Idem, 9–20.iv.2005; 2♀: Idem, 20–30.iv.2005; 1♂: Idem, 7–22.iii.2006; 2♂: Idem, 16–31.i.2006; 1♂: “United Arab Emirates, al-Ajban (4683–4690), Malaise tr[ap], 26.v.–25.vi.2006, 24°36'N, 55°01'E, A. v. Harten, RMNH’06”; 1♂: Idem, 1.iv.–2.v.2006; 1♀, 1♂: Idem, 17.x.–9.xi.2005; 1♀, 3♂: “United Arab Emirates, Hatta (6398), at light, 17–24.viii.2006, 24°49'N, 56°07'E, A. v. Harten, RMNH’06”; 1♀, 4♂: Idem, 19–28.iii.2006; 1♀: “United Arab Emirates, Sharjah x Khor Kalba (6599), light trap, 24°59'N, 56°09'E, 24–30.v.2006, A. v. Harten, RMNH’06”; 2♀: Idem, 31.v.–7.vi.2006; 1♀: “United Arab Emirates, SSW of ad-Dhaid (6154), light tr[ap], 24–30.v.2006, 25°09'N, 55°48'E, A. v. Harten, RMNH’06”; 45♀, 2♂: “**Yemen** (7501), Al Kadan, light trap, i.2003, A. v. Harten & T. Abdul-Haq, RMNH’03”; 1♀, 1♂: Idem, ix.2001; 5♀, 1♂: Idem, x.2001; 10♀, 1♂: Idem, v.2002; 1♀: Idem, iv.2002; 3♀: Idem, i.2003; 2♀, 2♂: “Yemen (8113), Al Mukalla, light trap, vii.–viii.2003, A. van Harten, RMNH’04”; 1♂: Idem, ix.–x.2003; 1♂: “Yemen, Seyun, light trap, 4–6.ix.2002, A. van Harten, RMNH’03”; 1♀, 1♂: Idem, 12–14.viii.2002; 2♀: Idem, 20–22.viii.2002; 1♂: Idem, xi.2002; 1♀: Idem, x.2001; 8♀: “Yemen (7533), Al Kowd, light trap, i.–iii.2003, A. van Harten & S. Al Haruri, RMNH’03”; 3♀, 1♂: Idem, iv.2001; 19♀: Idem, ix.2003; 4♀: Idem, vi.2002; 2♀: Idem, i.2001; 1♀: Idem, 8–12.vii.2001; 1♀: Idem, 17–21.vii.2001; 1♀, 1♂: Idem, 21–25.viii.2001; 2♀, 1♂: Idem, 27–31.vii.2001; 1♀: Idem, 6–10.viii.2001; 14♀, 1♂: Idem, 16–20.viii.2001; 3♀: Idem, 1–5.ix.2001; 2♀: Idem, ii.2000; 4♀, 1♂: Idem, iii.2000; 2♀: Idem, vii.2000; 6♀: Idem, viii.2000; 1♀: Idem, xii.2000; 8♀, 1♂: Idem, v.–vi.2000; 8♀: Idem, viii.1999; 1♀: Idem, vii.1999; 7♀: Idem, vii.–ix.1999; 2♀: Idem, ix.1999; 2♀: “Yemen: Ta’izz (6963), light trap, v.2002, A. van Harten & A.R. Al Yarimi, RMNH’98”; 3♀: Idem, vi.2002; 3♀: Idem, v.2000; 1♀: Idem, ix.2000; 1♀: Idem, x.1999; 1♀: Idem, xi.1999; 1♀: Idem, 26–28.vii.1999; 7♀: “Yemen (5486), Al Lahima, 1.i.–9.iv.2001, Mal[aise] trap, A. v. Harten, RMNH’02”; 2♀, 1♂: Idem, 17.ix.–14.xii.2001; 1♀: Idem, 9.iv.–5.vi.20012; 2♀: Yemen (7009), Lahj, viii.2002, Mal[aise] trap, A. v. Harten & A. Sallum, RMNH’02”; 1♀: Idem, i.2001; 1♀, 1♂: “Yemen: Ar Rujum (5556), 15.i.–9.iv.2001, Mal. trap, A. v. Harten, RMNH’02”; 1♀: Idem, 9.vi.–5.vi.2001; 3♀: Yemen (6667), 12 km NW Manakhah, Mal[aise] trap, 27.iii.–5.v.2002, A. v. Harten, RMNH’03”; 1♀, 1♂: “Yemen: Hamman’Ali (5404), from coffee-berries, 14.iii.2001, A. v. Harten, RMNH’02”.

**Figures 179–181.**
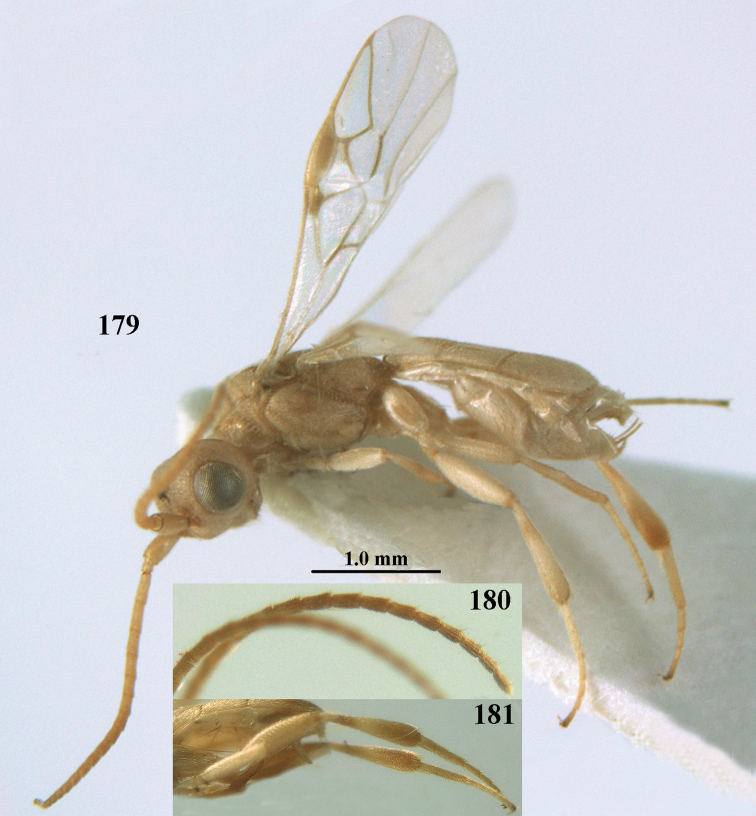
*Phanerotoma lepta* van Achterberg, sp. nov., ♀, holotype (but **180, 181** of ♂, paratype) **179** habitus lateral **180** apical half of antenna lateral **181** hind leg lateral.

##### Diagnosis.

**Figures 182–193.**
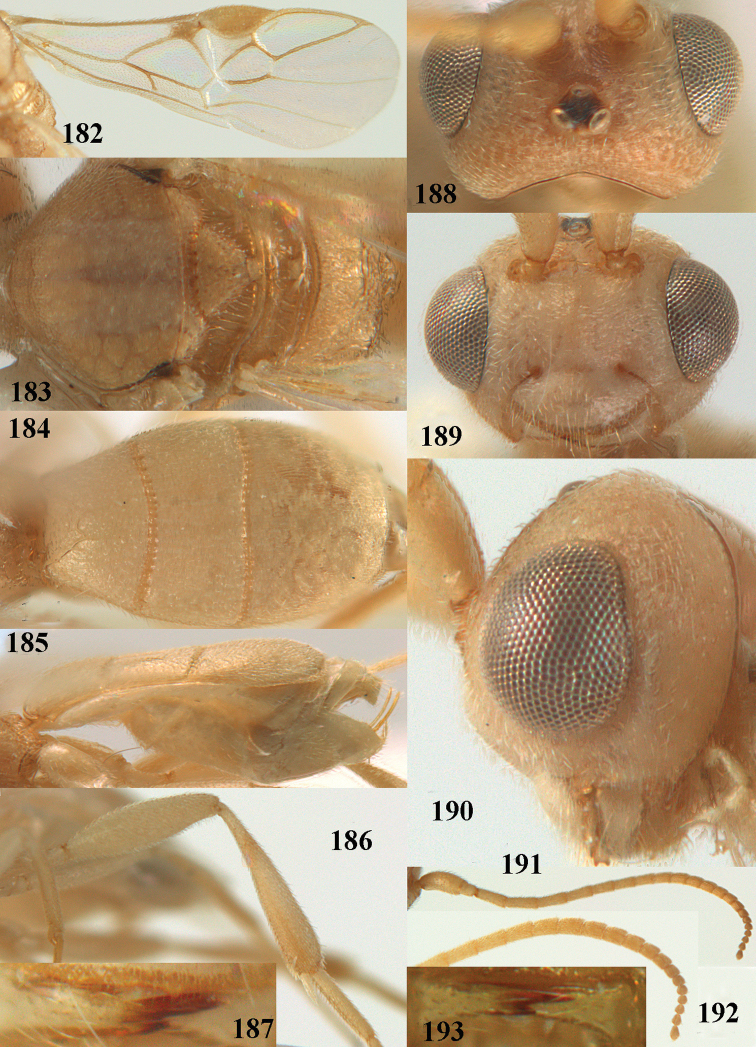
*Phanerotoma lepta* van Achterberg, sp. nov., ♀, holotype, (but **193** of ♀ paratype) **182** fore wing **183** mesosoma dorsal **184** first–third metasomal tergites dorsal **185** metasoma lateral **186** hind leg lateral **187, 193** mandible ventral **188** head dorsal **189** head anterior **190** head lateral **191** antenna lateral **192** apical half of antenna lateral.

Distance between apex of marginal cell and apex of fore wing 0.3–0.5 × vein 1-R1 and vertex finely rugulose; five–six apical antennal segments of ♀ suddenly small and moniliform compared to more basal segments (Fig. 192); ocelli often rather small (POL 0.8–1.0 × width of posterior ocellus; Fig. 188); third tergite of metasoma flattened in lateral view (Fig. 185); second submarginal cell of fore wing rather small (Fig. 182); anterior half of vein 1-M yellow; vein r 0.3–0.4 × vein 3-SR; head moderately emarginate medio-posteriorly (Fig. 188); hind femur comparatively stout; inner tooth of mandible minute, 0.2 × apical tooth; temple matt; hypopygium of ♀ ivory or brownish yellow; second submarginal cell smaller; face and clypeus frequently ivory. *Phanerotoma lepta* is similar to small *P. leucobasis*, but the ocelli are smaller, the apical antennal segments becoming suddenly smaller, head is less emarginate medio-posteriorly, third metasomal tergite is rather flat and angle of vein 2-SR with 3-SR is smaller.

##### Description.

Female, holotype, length of body (excluding ovipositor) 3.7 mm; antenna 2.75 mm; fore wing 2.8 mm; visible part of ovipositor sheath 0.3 mm (sparsely erect setose).

***Head*.** Width 1.5 × median length in anterior view and part of head above eye in lateral view 0.3 × height of eye (Fig. 190); antenna with 23 segments and slightly shorter than fore wing, five–six apical antennal segments suddenly (compared to more basal segments) small and moniliform (Fig. 192), with short bristle apically and apical segment without distinct spine, third, fourth and penultimate segments 2.4, 2.6 and 1.0 × longer than wide in lateral view, respectively; area of stemmaticum granulate; OOL: diameter of posterior ocellus: POL = 17: 5: 4; length of eye twice temple in dorsal view (Fig. 188); frons curved rugulose medially, rugose laterally and without distinct median carina; vertex aciculate and with satin sheen; temple finely and densely rugulose and rather dull; face finely rugose and with small median bump and rather shiny; clypeus superficially granulate, rather shiny and 0.8 × minimum width of face, intertentorial distance 2.4 × minimum width between clypeus and eye (Fig. 189), long erect setose and with three minute teeth medio-ventrally; eye medium-sized, strongly convex and in lateral view 1.3 × wider than temple (measured medially; Fig. 190), in anterior view its height 0.7 × minimum width of face; upper condyle of mandible near lower level of eyes (Fig. 189); malar space aciculate, with satin sheen and 0.6 × as long as basal width of mandible; lower tooth of mandible 0.2 × as long as apical tooth (Fig. 193).

***Mesosoma*** (Figs 179, 183). Length 1.5 × its width in lateral view; side of pronotum rugose; propleuron posteriorly evenly convex; mesosternum largely granulate and rather shiny; mesoscutum densely and finely reticulate-rugulose; scutellum distinctly granulate and rather matt; notauli hardly indicated; scutellar sulcus wide medially and narrow laterally, with nine carinae (Fig. 183); metanotum with median carina anteriorly and finely serrate posteriorly; propodeum rugose, medially with transverse carina, but no median carina, and latero-posteriorly weakly tuberculate. ***Wings*.** Fore wing 2.7 × longer than its maximum width; length of 1-R1 1.1 × as long as pterostigma; distance between wing apex and marginal cell apex 0.45 × length of vein 1-R1; r issued far beyond middle of pterostigma and 0.3 × 3-SR; 2-SR hardly curved and distally slightly converging to posterior margin of pterostigma (Fig. 182); SR1 straight; 2-SR+M indistinct, because of narrowly antefurcal m-cu; parastigma large; 1-CU1 0.5 × as long as vein 2-CU1, cu-a 0.8 × 1-CU1; r:3-SR:SR1 = 3:11:40; 2-SR:3-SR:r-m = 20:11:7; r-m reclivous; 2-M hardly curved (Fig. 182). Hind wing: M+CU:1-M:1r-m = 27:30:10. ***Legs*.** Hind femur matt, 3.6 × as long as wide and widened submedially; middle tibia with small ivory blister; inner spur of middle tibia 0.4 × its basitarsus; hind tibia moderately wide medially (Fig. 186); hind coxa largely coriaceous and rather matt.

***Metasoma*** (Figs 184, 185). Elliptical in dorsal view, 1.7 × as long as wide and 1.4 × as long as mesosoma; first and second tergites, as basal half of third tergite, densely and coarsely longitudinally rugose; second metasomal suture medium-sized; third tergite 1.6 × longer than second tergite and laterally curved, in lateral view rather flat, apical half rugulose-coriaceous and with satin sheen (Fig. 185), lateral lamella narrow, wider and not protruding latero-apically and medio-apically truncate and rather wide; ovipositor sheath parallel-sided, apically narrow (Fig. 185), its visible part 0.09 × as long as fore wing and 0.15 × metasomal carapace and sparsely setose part 0.06 × fore wing and with erect setae; hypopygium with medium-sized up curved apical triangle, apically without spine and densely setose (Fig. 185).

***Colour*.** Pale brownish yellow (including ovipositor sheath); apex of antenna and apex of hind tibia somewhat darkened; stemmaticum dark brown; face, clypeus, prothorax, tegulae, remainder of legs and metasoma ventrally ivory; parastigma and pterostigma (but basally pale yellowish) pale brownish; wing membrane slightly brownish below pterostigma; veins 1-M and m-cu of fore wing pale yellowish.

##### Male.

Similar to female with hind femur similar or slightly inflated (Fig. 181), antennal segments slender and elongate, apical segments bristly apically (Fig. 180).

##### Variations.

Length of fore wing of ♀ 1.7–2.8 mm, of ♂ 1.6–2.4 mm; vein 1-M of fore wing and parastigma yellowish, brown or largely dark brown.

##### Distribution.

United Arab Emirates, Yemen.

##### Biology.

Unknown.

##### Etymology.

Named derived from *leptos* (Greek for fine, small, thin or delicate) because of the small and delicate apical antennal segments of the female.

#### Phanerotoma leucobasis

Taxon classificationAnimaliaHymenopteraBraconidae

Kriechbaumer, 1894

9F06F584-F071-53C0-9599-2A3C7AA826CA

Figs 194–198 , 199–208

Phanerotoma leucobasis Kriechbaumer, 1894: 62; Shenefelt 1973: 919; van Achterberg and Polaszek 1996: 55–56.
Phanerotoma ornatulopsis De Saeger, 1948: 164, 186–188; Shenefelt 1973: 921; van Achterberg and Polaszek 1996: 55 [examined].
Phanerotoma ornatulopsis race tshegera De Saeger, 1948: 164, 188–190; Shenefelt 1973: 921. Invalid name.
Phanerotoma caboverdensis Hedqvist, 1965: 9; Zettel 1992: 293 (as synonym of P. flavitestacea Fischer). Syn. nov.

##### Type material.

The holotype of *P. leucobasis* from **Nigeria** is lost; but a ♀ from the neighbour country **Benin** (RMNH) agrees with the original description and belongs to the rather pigmented form with distinct subbasal dark brown patch of hind tibia as in *P. caboverdensis*. The holotypes of *P. ornatulopsis* and *P. caboverdensis* have been examined.

##### Additional material.

From **Saudi Arabia** (Jizan, ex leaftying Pyralid on *Tamarix*; Hail, ex Pyralid on *Euphorbia retusa*; Al Kharj, from grapes infested by *Cadra figulilella* Gr.; Hakimah; Wadi Uqdah; 16 km W Badr Hunayn; Wadi Daykah, 600 m), **Yemen** (Ta’izz; Mayfa’ah; Al Lahima; Al Kowd; Al Kadan; Jafa (ex *Prophantis* sp.); near Madinat ash Shirq (ex *Prophantis smaragdina* (Butler, 1875) in coffee beans); Seyun; Sana’a; Hamman’Ali) and **United Arab Emirates** (Fujairah; NARC near Sweihan; al-Ajban; Sharjah Desert Park; Wadi Bih dam; Sharjah x Khor Kalba; Hatta; Wadi Safad; Wadi Majdaq).

**Figures 194–198.**
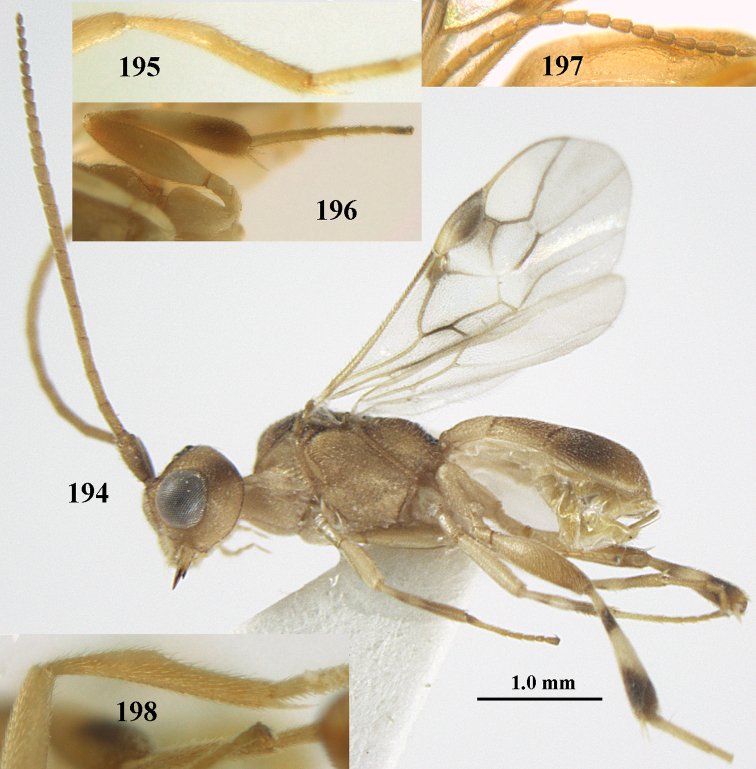
*Phanerotoma leucobasis* Kriechbaumer. ♀ (but **195** ♂), Yemen **194** habitus lateral **195** middle tibia lateral **196** hind leg lateral **197** apical half of antenna lateral **198** middle tibia lateral.

##### Diagnosis.

Area above eye of ♀ in lateral view 0.38–0.45 × height of eye; ocelli medium-sized (Fig. 204); OOL 2.8–3.0 × diameter of posterior ocellus; POL of ♀ 0.6–0.9 × diameter of posterior ocellus; posterior ocelli distinctly larger than anterior ocellus (Fig. 204); length of eye in dorsal view of ♀ 1.6–1.9 × temple (Fig. 204); anterior half of vein 1-M of fore wing dark brown, slightly paler than vein 1-CU1; subapical antennal segments of ♀ non-moniliform, somewhat longer than wide; antenna of ♀ moderately and gradually narrowed apically; head distinctly emarginate posteriorly in dorsal view; scapus, temple dorsally and frons medially sometimes brownish; length of ivory part of hind tibia usually medium-sized, in dorsal view ca. 0.45 × as long as tibia and tibia wide medially; dorsal border of third tergite in lateral view rather flat to rather convex; hind femur slightly less widened compared to *P. ocularis* (Fig. 203); hind tibia tricoloured in lateral view, but in Cabo Verdean specimens more or less bicoloured; dorsal border of third tergite in lateral view distinctly convex (Fig. 202); third metasomal tergite 1.6–1.7 × second tergite; malar space medium-sized; third tergite of ♀ slightly concave medio-posteriorly and tergite in dorsal view semi-circular, 0.7 × as long as its basal width (Fig. 201).

**Figures 199–208.**
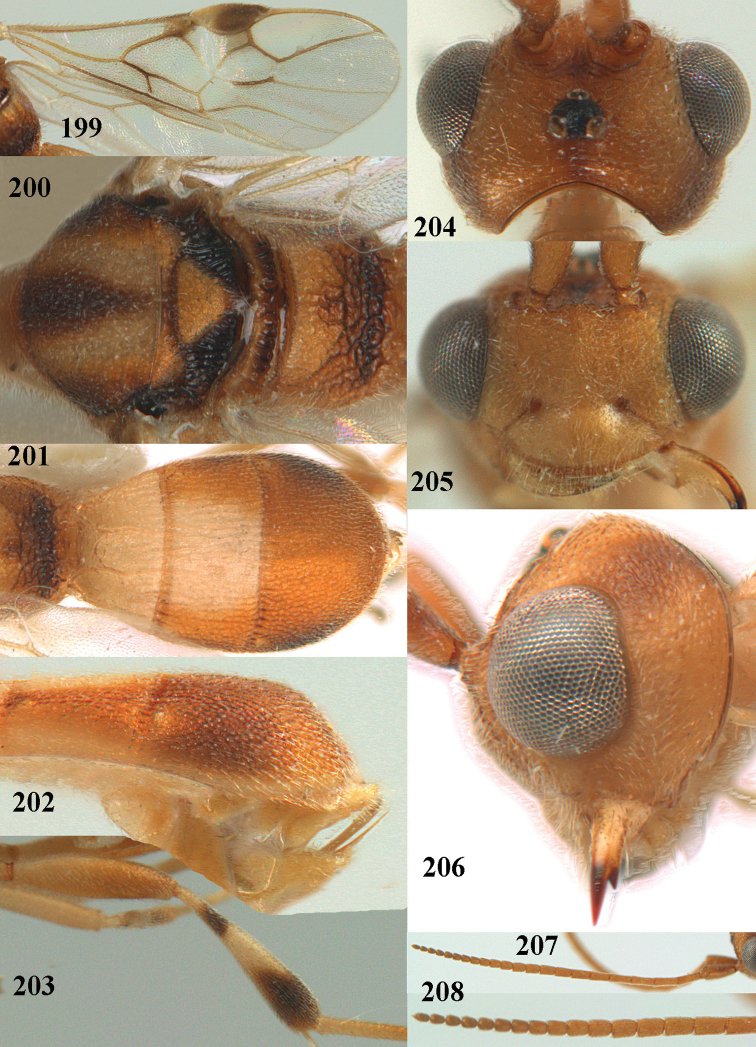
*Phanerotoma leucobasis* Kriechbaumer, ♀, Yemen **199** wings **200** mesosoma dorsal **201** first–third metasomal tergites dorsal **202** metasoma lateral **203** hind leg lateral **204** head dorsal **205** head anterior **206** head lateral **207** antenna lateral **208** apical half of antenna lateral.

Specimens from Cabo Verde (including holotype of *P. caboverdensis* Hedqvist, 1965) and Somalia have hind tibia usually with long ivory part (0.4 × length of tibia) in dorsal view; scapus yellowish and just surpassing level of posterior ocellus, head in dorsal view 1.9–2.4 × its median length, marginal cell ca. 2.8 × as long as high, parastigma partly yellowish, length of body of ♀ 3.7–4.8 mm and vein 1-R1 of fore wing 4.5–5.0 × distance from it to wing apex, but specimens from in Somalia (♀) have vein 1-R1 of fore wing ca. 3.5 × distance from it to wing apex. The holotype of *P. leucobasis* from Nigeria is lost, according to the original description it belonged to the rather pigmented form with distinct subbasal dark brown patch of hind tibia as seen in the holotype of *P. caboverdensis* and in *P. ornatulopsis* (including “race” *tshegera*) De Saeger, 1948, from Congo and therefore, are considered synonyms. Specimens from Congo have the metasoma 1.8–1.9 × longer than wide, combined length of first and second tergites 1.2–1.3 × as long as third tergite, middle tarsus sometimes slender and ivory part of hind tibia rather short (0.3 × length tibia).

##### Distribution.

*Saudi Arabia, * United Arab Emirates, *Yemen, Afrotropical region.

##### Biology.

Parasitoid of *Prophantis smaragdina* (Butler) (Crambidae) in coffee beans (new record), *Paramyelois transitella* (Walker), *Ectomyelois ceratoniae* (Zeller), (in laboratory) *Ephestia kuehniella* (Zeller) and *Cadra calidella* (Guinée) (Pyralidae) and *Platyedra gossypiella* (Saunders) (Gelechiidae). The flange at the third tergite apically may be narrow to rather wide and straight or somewhat emarginate, as shown by the reared series. The shape of the third tergite (especially of males) is rather variable, from rather convex and truncate to distinctly flattened in lateral view.

##### Notes.

*Phanerotoma leucobasis* Kriechbaumer was synonymized with *P. ocularis* by van Achterberg and Polaszek (1996), but after examination of more West African and Arabian specimens it was possible to separate this often more pigmented taxon as a separate species because of the smaller eyes and ocelli in combination with a slenderer female antenna.

#### Phanerotoma longivena sp. nov.

Taxon classificationAnimaliaHymenopteraBraconidae

C88695AF-39E3-5977-A232-AA75B54019D7

http://zoobank.org/A69D3EB4-BF9B-4ED8-8710-941D91409615

Figs 209 , 210–220

##### Type material.

***Holotype***, ♀ (RMNH), “**Yemen** (no. 3285), Ta’izz, light trap, 22–24.viii.1998, A. van Harten & Ahmad Ahwad, RMNH’99”. ***Paratype***: 1♀, “Yemen (7501), Al Kadan, light trap, i.2003, A. v. Harten & T. Abdul-Haq, RMNH’03”.

**Figure 209.**
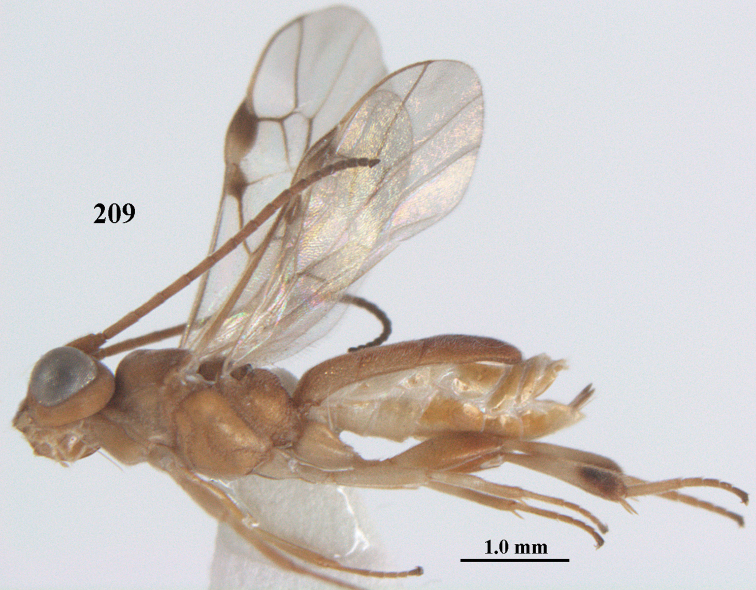
*Phanerotoma longivena* van Achterberg, sp. nov., ♀, holotype, habitus lateral.

##### Diagnosis.

**Figures 210–220.**
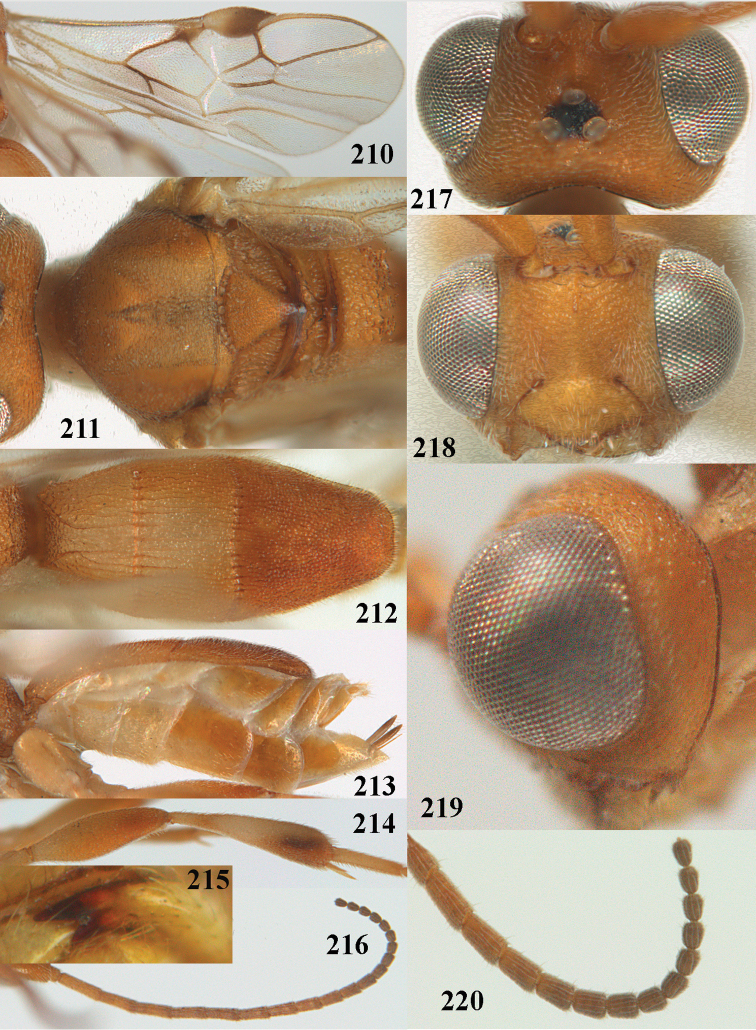
*Phanerotoma longivena* van Achterberg, sp. nov., ♀, holotype **210** wings **211** mesosoma dorsal **212** first–third metasomal tergites dorsal **213** metasoma lateral **214** hind leg, lateral **215** mandible ventral **216** antenna lateral **217** head dorsal **218** head anterior **219** head lateral **220** apical half of antenna (apical segment missing) lateral.

Among the species with the third metasomal tergite of ♀ 1.8–2.0 × as long as second tergite and tergite straight laterally easily to separate because of the long vein r (ca. 1.2 × as long vein 3-SR (Figs 209, 210); in other species 0.2–0.7 ×), the apical half of the pterostigma largely dark brown (entirely yellow or partly slightly darkened in other species), the inner tooth of mandible 0.8 × as long as apical tooth (Fig. 215; 0.1–0.3 ×), the eye in dorsal view ca. 3.4 × as long as temple (Fig. 217; 1.7–2.5 ×) and in lateral view ca. 2.3 × as long as temple (Fig. 219; 1.6–1.9 ×).

##### Description.

Female, holotype, length of body (excluding ovipositor) 4.7 mm; antenna 3.5 mm (but apical segment missing); fore wing 3.4 mm; visible part of ovipositor sheath 0.25 mm (setose part 0.15 mm).

***Head*.** Width 1.5 × median length in anterior view and part of head above eye in lateral view 0.15 × height of eye (Fig. 219); antenna with 22 segments (but apical segment missing) and slightly longer than fore wing, segments gradually shortened, narrowed apically and apical segments moniliform (Fig. 220), third, fourth and penultimate segments 3.6, 3.0 and 1.4 × longer than wide in lateral view, respectively; area of stemmaticum coriaceous; OOL: diameter of posterior ocellus: POL = 10: 4: 5; length of eye 3.4 × temple in dorsal view (Fig. 217); frons rugulose-coriaceous medially, rugose laterally and without median carina; vertex and temple finely and densely rugose, and rather dull; face densely and finely rugose and without distinct median ridge or median carina; clypeus mostly smooth, rather shiny and three minute teeth medio-ventrally (Fig. 218); eye large in lateral view and 2.3 × (measured medially) temple (Fig. 219), in anterior view slightly longer than minimum width of face (Fig. 218); upper condyle of mandible above lower level of eyes (Fig. 218); malar space coriaceous and 0.4 × as basal width of mandible; lower tooth of mandible 0.8 × as long as apical tooth (Fig. 215).

***Mesosoma*** (Figs 209, 211). Length 1.5 × its width in lateral view; side of pronotum medially transversely rugose, dorsally superficially rugulose; mesoscutum finely rugulose with granulate background, densely setose; notauli slightly indicated; scutellar sulcus rather narrow and with eight short crenulae (Fig. 211); scutellum triangular, finely granulate, matt; metanotum without median carina anteriorly and obtusely protruding posteriorly; propodeum coarsely reticulate, without median and transverse carinae, latero-posteriorly slightly tuberculate. ***Wings*.** Fore wing 2.7 × longer than its maximum width; length of 1-R1 1.5 × as long as pterostigma; r issued far beyond middle of pterostigma and 1.2 × 3-SR; 2-SR straight and distally converging with posterior margin of pterostigma (Fig. 210); SR1 straight; 2-SR+M absent because of interstitial m-cu; parastigma large; 1-CU1 0.4 × as long as vein 2-CU1; r:3-SR:SR1 = 12:10:59; 2-SR:3-SR:r-m = 26:10:8; r-m vertical; 2-M slightly curved (Fig. 210). Hind wing: M+CU:1-M:1r-m = 27:24:10. ***Legs*.** Hind femur 4.8 × as long as wide and widened subbasally; middle tibia with ivory blister; inner spur of middle tibia 0.4 × its basitarsus; hind coxa mostly smooth and shiny; hind tibia moderately widened medially (Fig. 214).

***Metasoma*** (Figs 212, 213). Elongate elliptical in dorsal view, 2.2 × as long as wide and 1.7 × as long as mesosoma; first and second tergites coarsely longitudinally rugose and rugae with interconnections; third tergite 1.8 × longer than second tergite, in lateral view rather flat, densely reticulate-rugulose and medio-posteriorly truncate (Fig. 212), lateral lamella not protruding latero-apically, medium-sized and medio-apically indistinct; setose part of ovipositor sheath moderately wide (Fig. 213), 0.04 × as long as fore wing and visible part of ovipositor sheath 0.07 × as long as fore wing and 0.13 × metasomal carapace; hypopygium apically with acute triangle (Fig. 213), without apical spine and with rather short setae.

***Colour*.** Brownish yellow; palpi, mandible (except dark brown teeth), tegula (paler than brownish humeral plate), legs (but hind tibia distinctly darkened apically and hind femur largely brownish) and metasoma ventrally pale yellow or ivory; apical half of flagellum brown; stemmaticum and scutellum medio-posteriorly dark brown; pterostigma dark brown with large pale yellowish basal spot and white near apex (Fig. 210); wing membrane slightly infuscate; parastigma (but yellowish anteriorly) and vein 1-M dark brown.

##### Male.

Unknown.

##### Variations.

Paratype: Very similar to holotype, length of fore wing of ♀ 3.1 mm; antenna of ♀ with 23 segments; metanotum with median carina anteriorly; propodeum weak irregular transverse carina; third tergite 1.8 × longer than second tergite and medio-apically with medium-sized lamella.

##### Biology.

Unknown.

##### Distribution.

Yemen.

##### Etymology.

Named after the comparatively long vein r of the fore wing.

#### Phanerotoma masiana

Taxon classificationAnimaliaHymenopteraBraconidae

Fahringer, 1934

1B100A86-6613-5FD6-A53D-55A99DE9D909

Figs 221–223 , 224–234

Phanerotoma ?parva Kokujev; Masi 1932: 434–435.
Phanerotoma parva var. masiana Fahringer, 1934: 573; Shenefelt 1973: 922.
Phanerotoma (Bracotritoma) masiana ; van Achterberg, 1990: 44 (as valid species).

##### Type material.

***Lectotype***, ♂, (Museum Genova): “[**Libya**,] Miss. Zool. a Cufra, Gialo, iv.1931”, “♀” [incorrect = ♂], “Typus”, “Ph. parva var. masiana Fahr.”; ***paralectotype***, 1 ♂, topotypic, but v.1931 and labelled “*Phanerotoma parva*”

##### Additional material.

Large series collected by light traps in **United Arab Emirates** (NARC near Sweihan; Wadi Safad; Hatta; Sharjah x Khor Kalba; Wadi Wurajah Farms; Sharjah Desert Park; Al-Ajban; Fujairah; Wadi Shawkah; near Mahafiz; SSW of ad-Dhaid; Wadi Maidaq) and **Yemen** (Al Kadan; Hamman; Al Kowd; Ta’izz; Seyun; Mayfa’ah); additional series examined from **Saudi Arabia** (Zalim; Wadi Tinan, 850 m; 16 km W. Badr Hu-nayn; Wadi Luotaie; Hakimah, 85 m) and **Egypt** (Wadi Isla, Sinai; Khamissa).

**Figures 221–223.**
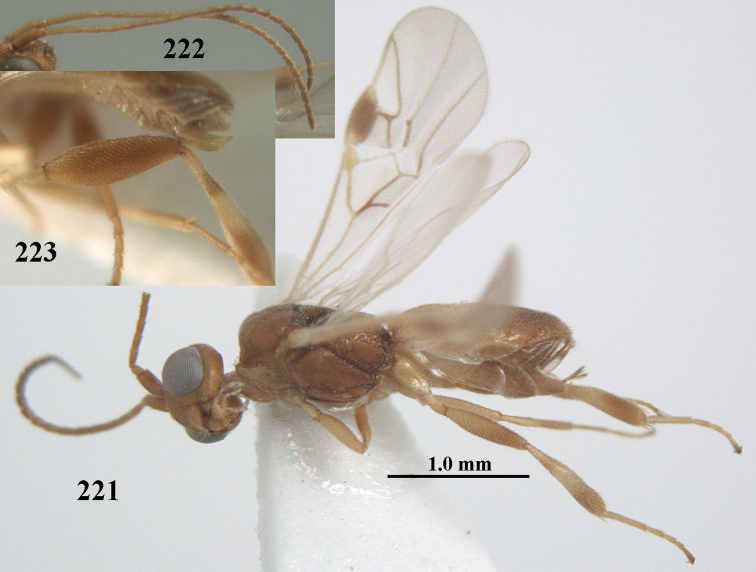
*Phanerotoma masiana* Fahringer, ♀ (but **222, 223** of ♂), UAE**221** habitus lateral **222** antenna lateral **223** hind femur and tibia lateral.

##### Diagnosis.

**Figures 224–234.**
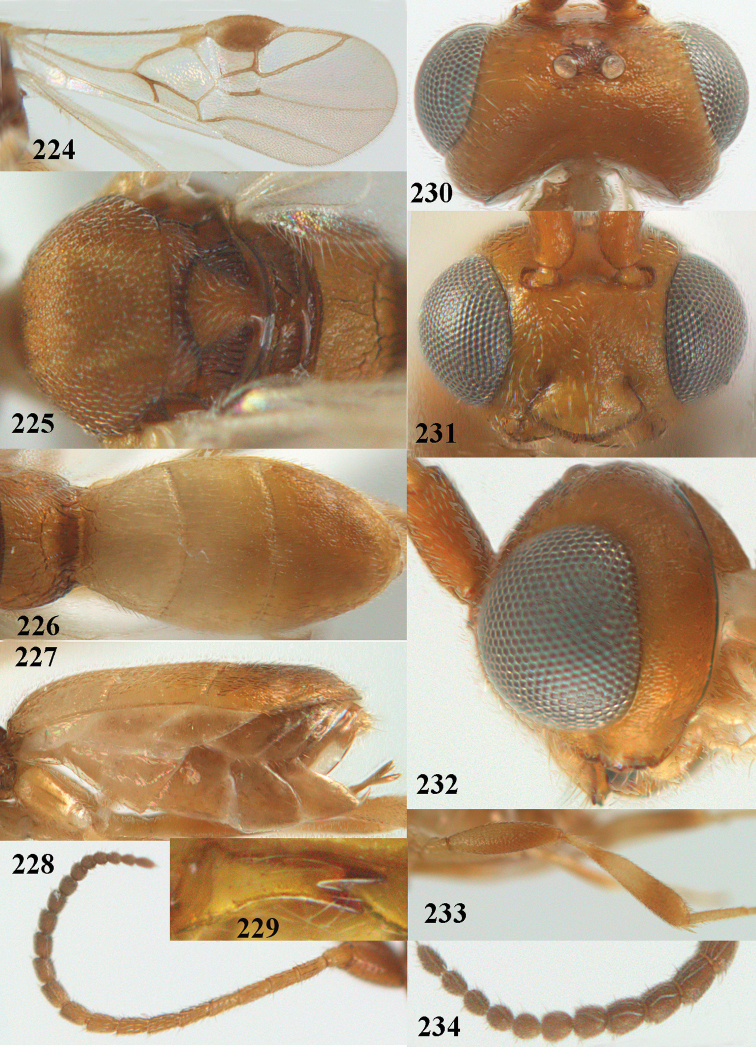
*Phanerotoma masiana* Fahringer, ♀, UAE**224** wings **225** mesosoma dorsal **226** first–third metasomal tergites dorsal **227** metasoma lateral **228** antenna lateral **229** mandible ventral **230** head dorsal **231** head anterior **232** head lateral **233** hind femur and tibia lateral **234** apical half of antenna lateral.

Ocelli medium-sized; upper condyles of mandibles near lower level of eyes (Fig. 231); antenna of ♀ with six–seven moniliform apical segments (Fig. 234); inner tooth of mandible ca. 0.7 × as long as apical tooth (Fig. 229); POL of ♀ ca. 1.1 × diameter of posterior ocellus (Fig. 230); area of mesosternum near mesosternal sulcus shiny and superficially granulate or smooth; parastigma large and yellow (Fig. 224); vein r of fore wing 1.2–2.3 × vein 3-SR of fore wing (Fig. 224); vein 1-R1 0.9–1.6 × distance from 1-R1 to wing apex; maximum width of pterostigma ca. 4.5 × vein 3-SR of fore wing (Fig. 224); medial length of third metasomal tergite 1.2–1.5 × length of second tergite, third tergite acute apically in lateral view and without transverse depression, partly smooth and shiny medio-dorsally (Figs 226, 227); length of fore wing (1.5–)1.9–2.9 mm, of body 2.5–3.6 mm.

##### Distribution.

Egypt, Iran, Libya, Saudi Arabia, *United Arab Emirates, *Yemen.

##### Biology.

Unknown. Collected mainly in January–April in UAE and mainly in May–December in Yemen.

##### Notes.

If antenna of ♀ slender submedially, upper condyles of mandible above lower level of eyes, hind femur wider, length of fore wing ca. 1.8 mm and pterostigma slenderer, cf. *P. ebneri* Fahringer, 1924, from Sudan.

#### Phanerotoma mesocellata sp. nov.

Taxon classificationAnimaliaHymenopteraBraconidae

49F43ADF-66C5-5366-AEFD-8F4815B1E4D5

http://zoobank.org/7BC291B3-A91B-4EEC-AEB2-15B1767E1C31

Figs 235–238 , 239–249

##### Type material.

***Holotype***, ♀ (RMNH), “**United Arab Emirates**, Sharjah Desert Park (15613), light trap, 1–30.iv.2007, 25°17'N, 55°42'E, A. v. Harten, RMNH’10”. ***Paratypes***: 1♀: Idem, 22.iii.–5.iv.2009; 1♀: Idem, 29.iii.–6.iv.2005; 5♀, 1♂: Idem, 23–30.iv.2005; 13♀, 2♂: Idem, 30.iv.–7.v.2005; 11♀: Idem, 6–13.iv.2005; 1♀: Idem, 21–29.iii.2005; 1♀: “United Arab Emirates, Sharjah (2279), light trap, 30.vi.–21.vii.2005, 25°21'N, 55°24'E, A. v. Harten, RMNH’05”; 1♀: Idem, 2.v.–5.vi.2005; 3♀: “United Arab Emirates, Fujairah (2438), light trap, 2.v.–5.vi.2005, 25°08'N, 56°21'E, A. v. Harten, RMNH’06”; 1♀: Idem, 5–24.iii.2005; 6♀: “United Arab Emirates, NARC near Sweihan (1245), light trap, 28.iii.–2.iv.2005, 24°24'N, 55°26'E, A. v. Harten, RMNH’06”; 4♀: Idem, 2–9.iv.2005; 22♀, 3♂: Idem, 9–20.iv.2005; 1♀: Idem, 14–28.iii.2005; 8♀, 3♂: “United Arab Emirates, Wadi Bih dam (11366), light trap, 24.iv.–23.v.2007, 25°48'N, 56°04'E, A. v. Harten, RMNH’10”; 4♀, 1♂: Idem, 13–30.iv.2008; 1♀: Idem, 19.ii.–29.iii.2007; 1♀: “United Arab Emirates, Hatta (11572), light trap, 21.vi.–19.vii.2006, 24°49'N, 56°07'E, A. v. Harten, RMNH’09”; 1♂: “United Arab Emirates, Sharjah x Khor Kalba (11542), light trap, 24°59'N, 56°09'E, 28.iii.–5.iv.2006, A. v. Harten, RMNH’10”; 4♀: “**Yemen** (5697), Al Kowd, light trap, iv.2001, A. van Harten & S. Al Haruri, RMNH’02”; 5♀: Idem, 16–20.viii.2001; 6♀: Idem, 8–12.vii.2001; 22♀, 1♂: Idem, 27–31.vii.2001; 5♀: Idem, 6–10.viii.2001; 7♀: Idem, v.–vi.2000; 14♀, 3♂: Idem, vii.1999; 2♀: Idem, ii.2000; 1♀: Idem, vii.2000; 7♀: Idem, viii.2000; 2♀: Idem, xii.2000; 1♀: Idem, viii.1999; 3♀: Idem, 21–25.viii.2001; 2♀, 1♂: Idem, vii.–ix.2001; 2♀: Idem, vi.2002; 4♀: Idem, ix.2003; 3♀: Idem, i.–iii.2003; 15♀, “Yemen (6090), Al Kadan, light trap, x.2001, A. van Harten & T. Abdul-Haq, RMNH’03”; 2♀: Idem, iv.2002; 14♀, 1♂: Idem, v.2002; 2♀, 1♂: Idem, i.2003; 7♀: Idem, xi.2001; 9♀: “Yemen (5404), Hamman’Ali, from coffee berries (with *Ceratitis capitata*?), 14.iii.2001, A. van Harten, RMNH’02”; 4♀: “Yemen (6381), Ta’izz, light trap, ix.–x.2001, A. van Harten & A.R. Al Yarimi, RMNH”; 1♀: Idem, ix.1999; 3♀: Idem, x.1999; 6♀: Idem, xi.1999; 4 ♀: Idem, xii.1999; 8♀: Idem, i.2000; 12♀: Idem, v.2000; 17♀: Idem, ix.2000; 2♀, 1♂: Idem, 5.i.–2.ii.1998; 15♀: Idem, 26–28.vii.1999; 16♀, 1♂: Idem, 3–24.i.1999; 17♀, 1♂: Idem, viii.2000; 1♀: Idem, 27–31.vii.2001; 7♀: Idem, x.2001; 3♀: Idem, iii.–iv.2001; 5♀: Idem, vi.2002; 6♀, 1♂: Idem, vii.2002; 6♀: “Yemen (3645), Sana’a, light trap, iii.–iv.1999, A. van Harten, RMNH’00”; 1♀: Idem, v.1999; 1♀: “Yemen (6667), 12 km NW Manakhah, Mal[aise] trap, 27.iii.–5.v.2002, A. v. Harten, RMNH’03”; 1♀: “Yemen, Seyun, light trap, 4–6.ix.2002, A. van Harten, RMNH’03”; 3♀: “Yemen (6158), Al Lahima, 17.ix.–14.xi.2001, Mal[aise] trap, A. v. Harten, RMNH’02”.

**Figures 235–238.**
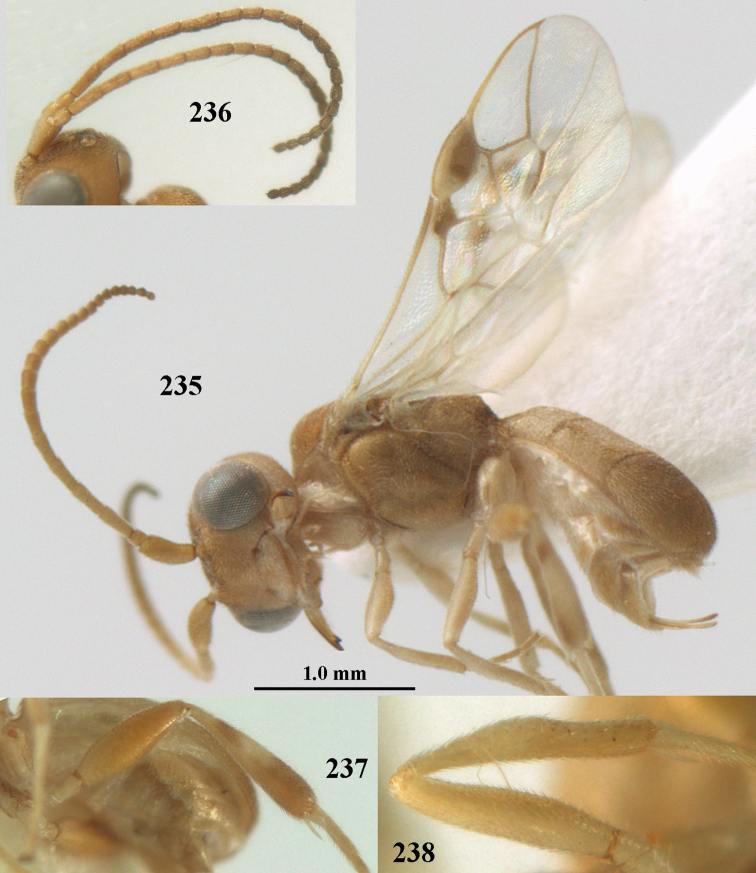
*Phanerotoma mesocellata* van Achterberg, sp. nov., ♀ holotype (but **236, 237** of ♂, paratype) **235** habitus lateral **236** antenna **237** hind femur and tibia lateral **238** middle femur and tibia lateral.

##### Diagnosis.

**Figures 239–249.**
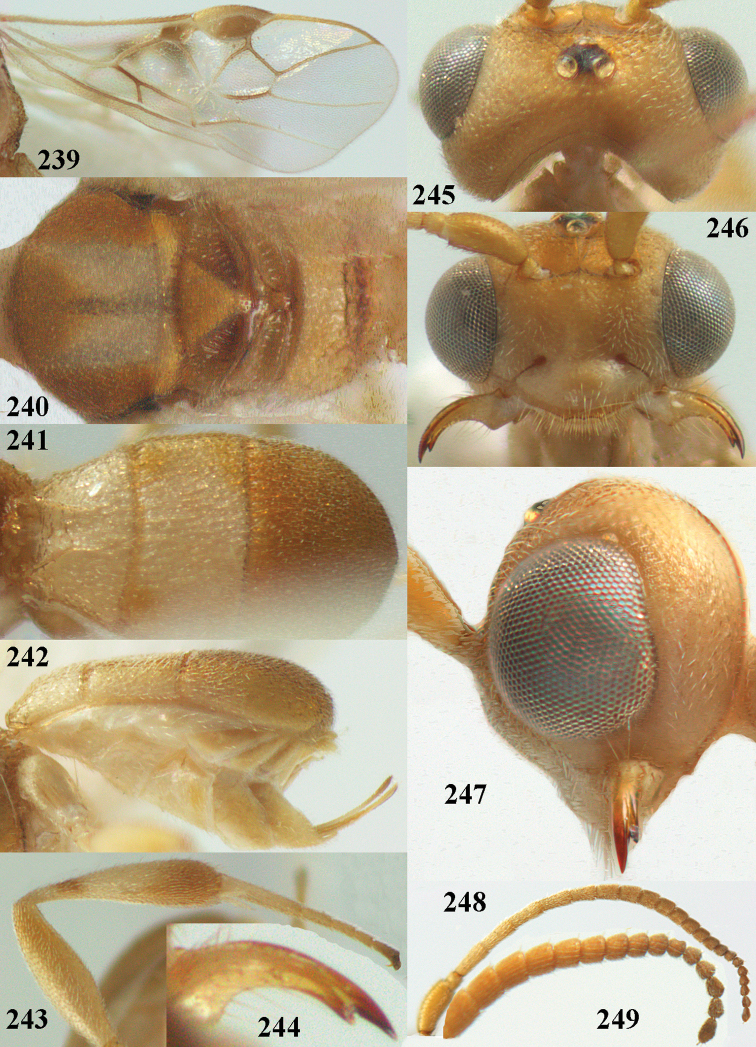
*Phanerotoma mesocellata* van Achterberg, sp. nov., ♀, holotype **239** fore wing **240** mesosoma dorsal **241** first–third metasomal tergites dorsal **242** metasoma lateral **243** hind leg lateral **244** mandible ventral **245** head dorsal **246** head anterior **247** head lateral **248** antenna lateral **249** apical half of antenna lateral.

Eighth–tenth antennal segments from apex of ♀ moderately moniliform, stocky, matt or slightly shiny, 14^th^ segment from apex somewhat longer than wide (Figs 248, 249); stemmaticum black or dark brown, but sometimes brownish yellow; mesosternum more or less shiny; second submarginal cell of fore wing comparatively short (Fig. 239); POL of ♀ 0.4–0.6 × width of posterior ocellus; eye 1.2–1.8 × as wide as median width of temple in lateral view (Fig. 247); vein 1-M (as usually parastigma) slightly darker than yellow M+CU1 of fore wing (Fig. 235); ovipositor sheath narrow apically (Figs 235, 242). Closely related to *P. ocularis* and differs mainly by the shape of the apical antennal segments of the female, the more curved vein 2-SR and the size of the ocelli.

##### Description.

Female, holotype, length of body (excluding ovipositor) 3.6 mm; antenna 2.7 mm; fore wing 2.6 mm; visible part of ovipositor sheath 0.4 mm (erect setae mostly concentrated at apex).

***Head*.** Width 1.7 × median length in anterior view and part of head above eye in lateral view 0.25 × height of eye (Fig. 247); antenna with 23 cylindrical segments, slightly widened submedially and slightly longer than fore wing, seven apical antennal segments small and moniliform (Fig. 249), with short bristles and apical segment with spine, third, fourth and penultimate segments 2.6, 2.4 and 1.4 × longer than wide in lateral view, respectively; area of stemmaticum coriaceous; OOL: diameter of posterior ocellus: POL = 15: 5: 3; length of eye 2.2 × temple in dorsal view (Fig. 245); frons with weak median carina, mainly coriaceous, rather shiny and laterally rugulose; vertex rugulose-coriaceous and rather matt, posteriorly also with some transverse rugulae and distinctly emarginate (Fig. 245); temple mainly coriaceous and rather matt, nearly parallel-sided in lateral view (Fig. 247), gradually narrowed behind eyes; face transversely rugose laterally, rugulose and with obsolescent median bump and with satin sheen; clypeus smooth, moderately shiny and 0.9 × as wide as minimum width of face, intertentorial distance 3.6 × minimum width between clypeus and eye, long erect setose and with three distinct blunt teeth medio-ventrally (Fig. 246); eye large, strongly convex and in lateral view 1.8 × wider than temple (measured medially; Fig. 247), in anterior view its height 0.8 × minimum width of face (Fig. 246); upper condyle of mandible above lower level of eyes (Fig. 246); malar space mostly smooth, rather shiny and 0.4 × as long as basal width of mandible; lower tooth of mandible 0.2 × as long as apical tooth, small (Fig. 244).

***Mesosoma*** (Figs 235, 240). Length 1.5 × its width in lateral view; side of pronotum coriaceous dorsally and remainder rugose; posteriorly propleuron bulging near central groove; mesosternum finely granulate and with satin sheen; mesoscutum densely reticulate-rugose on granulate background, with satin sheen, notauli absent; scutellum nearly flat, finely granulate-rugulose; scutellar sulcus medium-sized, with eight carinae (Fig. 240); metanotum with median carina and minute medio-posterior tooth, its posterior border finely serrate; propodeum coarsely reticulate-rugose, on median-sized dorsal face less coarsely rugose, with transverse carina, no median carina, and latero-posteriorly weakly tuberculate. ***Wings*.** Fore wing 2.7 × longer than its maximum width; 1-R1 1.3 × as long as pterostigma; distance between wing apex and marginal cell apex 0.25 × length of 1-R1; r issued far beyond middle of pterostigma and 0.2 × 3-SR; 2-SR weakly curved and slightly converging to posterior margin of pterostigma (Fig. 239); SR1 curved; m-cu interstitial; parastigma large; 1-CU1 0.5 × as long as vein 2-CU1, cu-a 0.9 × 1-CU1, strongly inclivous; r:3-SR:SR1 = 4:19:49; 2-SR:3-SR:r-m = 28:19:9; r-m reclivous; 2-M slightly curved (Fig. 239). Hind wing: M+CU:1-M:1r-m = 22:17:10. ***Legs*.** Hind femur rather matt, 3.3 × as long as wide and robust; hind tibia swollen (Fig. 243); middle tibia with medium-sized blister; inner spur of middle tibia 0.5 × its basitarsus; hind coxa largely superficially granulate, but dorsally partly smooth and rather shiny.

***Metasoma*** (Figs 241, 242). Oval in dorsal view, 1.6 × as long as wide and 1.2 × as long as mesosoma; first and second tergites irregularly and coarsely longitudinally rugose; second metasomal suture rather wide and straight; third tergite 1.5 × longer than second tergite and laterally curved, convex medially, rounded posteriorly in dorsal view (Fig. 241), obtuse posteriorly in lateral view (Fig. 242), densely reticulate-rugose and with satin sheen (Fig. 241), lateral lamella narrow basally, but widened near apical half of third tergite and latero-apically, medio-apically somewhat barrower and concave; ovipositor sheath narrow, apically slightly widened and darkened (Fig. 242), its visible part 0.16 × as long as fore wing and 0.28 × metasomal carapace, and with erect setae mainly near its apex; hypopygium of ♀ with short widely triangular and up curved apical protuberance (Fig. 242) and with mostly medium-sized setae.

***Colour*.** Pale brownish yellow; stemmaticum blackish; apex of antenna, hind tibia apically and subbasally and apex of ovipositor sheath rather brown; parastigma partly brownish, remainder and vein 1-M yellow; clypeus, palpi, tegulae, remainder of legs, mesoscutum medio-posteriorly, first and second tergites and metasoma ventrally pale yellowish or ivory; pterostigma (but basally and apically pale yellowish) and most veins brown; wing membrane slightly brownish below pterostigma and near vein 1-CU1.

##### Male.

Similar to female (including hind femur; Fig. 237), but antenna slenderer (Fig. 236).

##### Variations.

Length of fore wing of ♀ 2.1–2.7 mm (mainly UAE; many specimens from Yemen are larger (2.9–3.6 mm) because of using larger hosts), of ♂ 2.1–2.2 mm (UAE; Yemen 2.3–3.2 mm); vein 1-M of fore wing and parastigma pale yellowish, but sometimes more or less brown; second tergite brownish yellow or brown laterally; stemmaticum usually dark brown, but sometimes brownish yellow.

##### Biology.

Unknown.

##### Distribution.

United Arab Emirates, Yemen.

##### Etymology.

Named after its intermediate sized ocelli (*meso* is Greek for middle).

#### Phanerotoma microdonta sp. nov.

Taxon classificationAnimaliaHymenopteraBraconidae

6010A2AF-F70E-5007-BF2D-D05B816925A4

http://zoobank.org/4DCFC3F6-5929-4357-BAEC-6874559DA11E

Figs 250–252 , 253–263

##### Type material.

***Holotype***, ♀ (RMNH), “**United Arab Emirates**, Sharjah Desert Park (2049–2074), light tr[ap], 30.iv.–31.v.2005, 25°17'N, 55°42'E, A. v. Harten, RMNH’05”. ***Paratypes***: 1♀: Same data as holotype; 8♀: Idem, 21–29.iii.2005; 7♀, 2♂: Idem, 29.iii.–6.iv.2005; 5♀, 8♂: Idem, 6–13.iv.2005; 8♀, 17♂: Idem, 13–23.iv.2005; 5♀: Idem, 30.iv.–7.v.2005; 13♀, 9♂: Idem, 23–30.iv.2005; 4♀, 5♂: Idem, 13–23.iv.2005; 3♀, 1♂: Idem, 22.ii.–9.iii.2005; 1♀, 1♂: Idem, 25.i.–22.ii.2005; 1♀: Idem, 17.ii.–3.iii.2007; 1♀: Idem, 4.viii.–4.ix.2008; 1♀: “United Arab Emirates, al-Ajban (11858), light trap, 17.iv.–29.v.2006, 24°36'N, 55°01'E, A. v. Harten, RMNH’10”; 1♂: Idem, 22.x.–9.xi.2005, Malaise trap; 1♀: Idem, 1.iv.–2.v.2006, Malaise trap; 1♂: Idem, 25.iii.–2.iv.2006, Malaise trap; 1♀, 1♂: Idem, 7–28.xii.2006, Malaise & light trap; 1♂: “United Arab Emirates, N of Ajman (6338–6342), water traps, 9–28.xii.2006, A. v. Harten, RMNH’07”; 1♀: Idem, 1.ii.–16.iii.2009; 2♀: “United Arab Emirates, NARC near Sweihan (1410), light trap, 1.ii.–14.iii.2005, 24°24'N, 55°26'E, A. v. Harten, RMNH’05”; 1♂: Idem, 9–20.iv.2005; 1♀: Idem, 28.iii.–2.iv.2005; 1♂: “United Arab Emirates, Sharjah (1840–1889), light trap, 6–30.vi.2005, 25°21'N, 55°24'E, A. v. Harten, RMNH’05”; 2♂: “United Arab Emirates, Hatta (11572), light trap, 21.vi.–19.vii.2006, 24°49'N, 56°07'E, A. v. Harten, RMNH’09”; 3♀: “**Yemen** (5404), Haman ‘Ali, from coffee berries (with *Ceratitis capitata*?), 14.iii.2001, A. van Harten, RMNH’02”; 5♀, “Yemen (7501), Al Kadan, light trap, i.2003, A. van Harten & A.R. Al Yarimi, RMNH’03”; 3♀, 3♂: “Yemen (8136), Al Kowd, light trap, ix.2003, A. van Harten & S. Al Haruri, RMNH’03”; 2♀: Idem, 27–31.vii.2001; 1♂: Idem, 8–12.vii.2001; 1♀: Idem, vi.2002; 1♀: Idem, i.–iii.2003; 1♂: Idem, v.–vi.2000; 1♀: Idem, viii.2000; 3♀: Idem, ix.1999; 1♀: Idem, xi.2000; 1♀: Idem, vii.–ix.2001; 2♀: Idem, 17–21.vii.2001.

##### Diagnosis.

Marginal cell of fore wing small, distance between wing apex and vein 1-R1 0.7–1.2 × as long as pterostigma (Fig. 253); pterostigma much wider than length of vein 3-SR; temple mainly granulate; parastigma rather large and yellow; vein 1-M of fore wing pale yellowish; second tooth of mandible ca. 0.3 × length of apical tooth (Fig. 258); hind tibia with distinct subbasal dark brown patch, rarely only faintly indicated. Easily confused with *P. masiana* Fahringer, from Arabian Peninsula, Egypt, Iran and Libya, but inner tooth of mandible small, ca. 0.3 × as long as apical tooth (medium-sized to large and 0.6–0.8 × in *P. masiana*); hind tibia usually with distinct subbasal dark patch (usually absent) and third metasomal tergite evenly sculptured and rather matt (rarely intermediate; partly smooth and shiny medially). Similar to *P. stenochora*, but differs by the densely sculptured third metasomal tergite (smooth in *P. stenochora*), distance between wing apex and vein 1-R1 0.6–1.1 × vein 1-R1 (ca. twice), temple granulate (finely rugose), near apical third of antenna of ♀ without shortened segments (present), and first discal cell of fore wing much higher than first subdiscal cell (approx. equally high).

**Figures 250–252.**
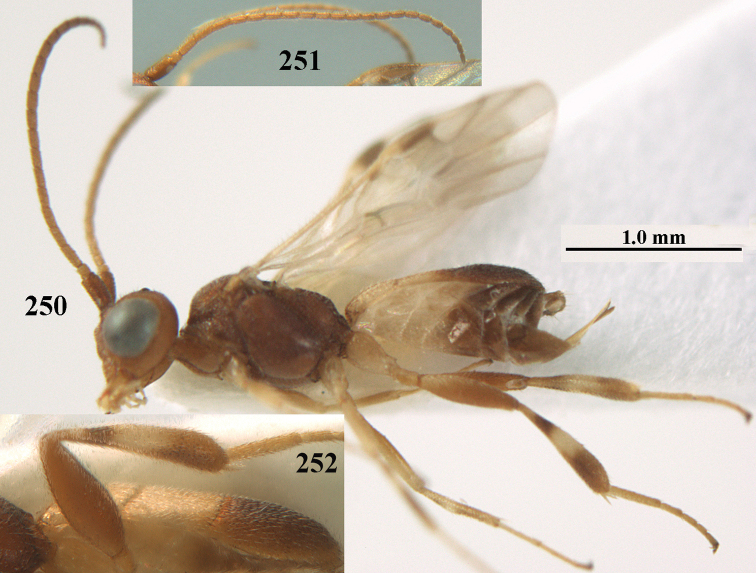
*Phanerotoma microdonta* van Achterberg, sp. nov., ♀, holotype (but **251, 252** of ♂, paratype) **250** habitus lateral **251** antenna **252** hind femur and tibia lateral.

##### Description.

Female, holotype, length of body (excluding ovipositor) 2.6 mm; antenna 2.0 mm; fore wing 2.0 mm; visible part of ovipositor sheath 0.4 mm (only apex setose).

***Head*.** Width 1.4 × median length in anterior view and part of head above eye in lateral view 0.2 × height of eye (Fig. 261); antenna with 23 segments and as long as fore wing, near apical third segments elongate and longer than wide, narrowed apically and five apical segments moniliform (Figs 262, 263) and apical segment with minute spine, third, fourth and penultimate segments 2.4, 2.0 and 1.0 × longer than wide in lateral view, respectively; area of stemmaticum granulate; OOL: diameter of posterior ocellus: POL = 10: 4: 5; length of eye 3.8 × temple in dorsal view (Fig. 259); frons granulate-rugulose laterally, largely granulate medially and without median carina; vertex granulate-rugulose and rather shiny; temple granulate and with satin sheen; face granulate and with small median bump dorsally but no distinct median carina; clypeus superficially granulate, rather shiny and distinctly narrower than face and with two minute teeth medio-ventrally (Fig. 260); eye large, strongly convex and in lateral view 2.5 × (measured medially) temple (Fig. 261), in anterior view slightly higher than minimum width of face; upper condyle of mandible below lower level of eyes (Fig. 260); malar space granulate, with satin sheen and 0.9 × as long as basal width of mandible; lower tooth of mandible 0.3 × as long as apical tooth (Fig. 258).

**Figures 253–263.**
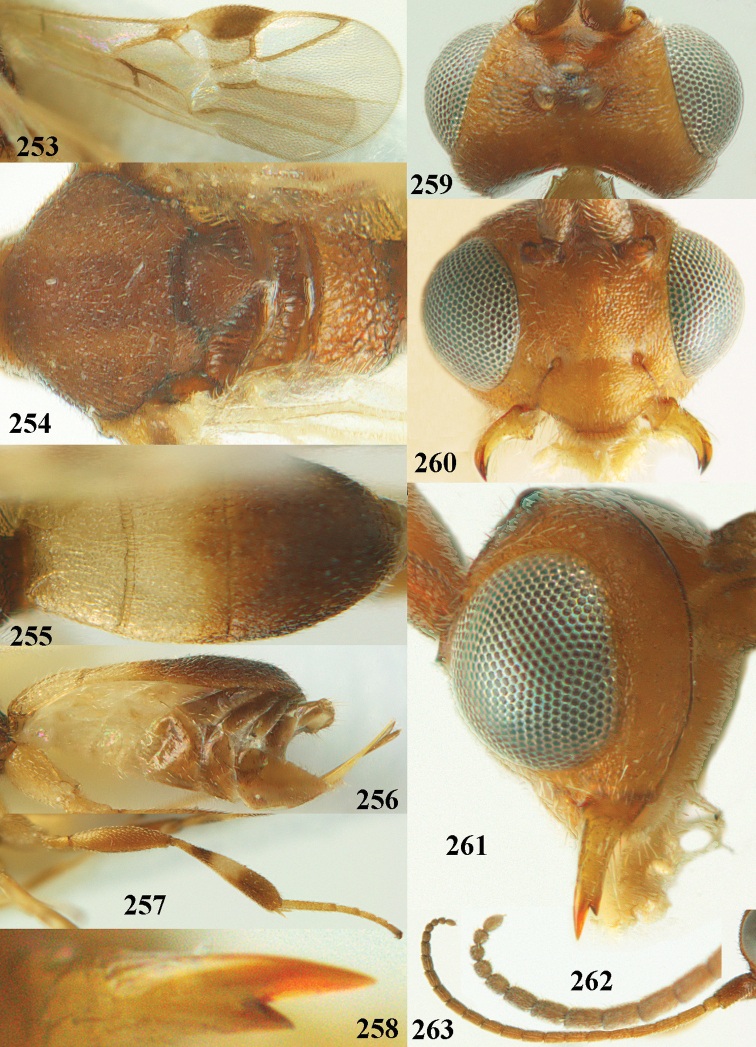
*Phanerotoma microdonta* van Achterberg, sp. nov., ♀, holotype **253** fore wing **254** mesosoma dorsal **255** first–third metasomal tergites dorsal **256** metasoma lateral **257** hind leg lateral **258** mandible ventral **259** head dorsal **260** head anterior **261** head lateral **262** apical half of antenna lateral **263** antenna lateral.

***Mesosoma*** (Figs 250, 254). Length 1.5 × its width in lateral view; side of pronotum rugose, but antero-medially and postero-ventrally partly smooth; propleuron posteriorly evenly convex; mesosternum largely superficially granulate and shiny; mesoscutum densely rugulose combined with granules in between; scutellum distinctly granulate and rather matt; notauli not indicated; scutellar sulcus narrow and with ten carinae (Fig. 254); metanotum without median carina anteriorly and no tooth posteriorly; propodeum coarsely reticulate-rugose, without distinct median and transverse carinae, and latero-posteriorly not tuberculate. ***Wings*.** Fore wing 2.8 × longer than its maximum width; length of 1-R1 0.8 × as long as pterostigma; distance between wing apex and vein 1-R1 equal to length of vein 1-R1; r issued far beyond middle of pterostigma and 2.5 × 3-SR; 2-SR nearly straight and distally slightly converging to posterior margin of pterostigma (Fig. 253); SR1 straight; 2-SR+M absent, m-cu interstitial; parastigma medium-sized to rather large; first discal cell of fore wing higher than first subdiscal cell; 1-CU1 0.4 × as long as vein 2-CU1; r:3-SR:SR1 = 5:2:31; 2-SR:3-SR:r-m = 15:2:6; r-m vertical; 2-M slightly curved (Fig. 253). Hind wing: M+CU:1-M:1r-m = 23:16:9. ***Legs*.** Hind femur matt, 4.0 × as long as wide and widened submedially; middle tibia with ivory blister; inner spur of middle tibia 0.4 × its basitarsus; hind coxa largely coriaceous and rather matt; hind tibia rather wide medially (Figs 250, 257).

***Metasoma*** (Figs 255, 256). Elliptical in dorsal view, twice as long as wide and 1.3 × as long as mesosoma; first and second tergites densely and finely rugulose; second metasomal suture narrow and first suture much wider; third tergite 1.6 × longer than second tergite and laterally curved, in lateral view rather flat (Fig. 256), largely coriaceous-granulate and with satin sheen (Fig. 255), lateral lamella narrow, not protruding latero-apically and medio-apically truncate and medium-sized; ovipositor sheath narrow (Fig. 256), its visible part 0.2 × as long as fore wing and 0.3 × metasomal carapace and only its apex with few rather long setae; hypopygium apically with robust triangle (Fig. 256), without apical spine and sparsely setose.

***Colour*.** Head (except clypeus) scapus, apex of antenna and ovipositor sheath brown; clypeus, propleuron, tegulum and remainder of antenna brownish yellow; humeral plate, third tergite, apex of second tergite, posterior half of metasoma ventrally, pterostigma (but basally pale yellowish), most veins, apical half of tibiae, middle and hind tibiae subbasally more or less dark brown; palpi, mandible (except dark brown teeth) and legs pale yellowish, but tibiae submedially, first and second tergites and basal half of metasoma ventrally ivory; wing membrane basally and marginal cell hyaline, remainder of apical half of fore wing slightly brownish; parastigma, veins 1-M and m-cu of fore wing pale yellow.

##### Male.

Similar to female with hind femur similar or inflated (Fig. 252) and antennal segments slenderer (Fig. 251).

##### Variations.

Length of fore wing of ♀ (1.3–)1.6–2.5 mm, of ♂ 1.4–2.0 mm; clypeus brown or brownish yellow; first–third tergites distinctly sculptured (typical) to (rarely) nearly smooth and shiny; third metasomal tergite 1.5–1.6 × longer than second tergite, dark brown or brown; apical 0.3–0.5 of antenna dark brown; scapus and head sometimes more or less brownish yellow; vein 3-SR 1.1–2.7 × as long as vein r; hind femur largely and apex of hind tibia sometimes dark brown.

##### Biology.

Unknown.

##### Distribution.

United Arabian Emirates, Yemen.

##### Etymology.

Named after short inner tooth of the mandible (*mikros* is Greek for small and *odontos* is Latin for tooth).

#### Phanerotoma micrommata sp. nov.

Taxon classificationAnimaliaHymenopteraBraconidae

48DC5918-4894-58D2-8C6F-D38D6D9AC111

http://zoobank.org/A2217CEA-A44C-4CD8-970E-2C57C6159C11

Figs 264 , 265–275

##### Type material.

***Holotype***, ♀ (RMNH): “**United Arab Emirates**, NARC near Sweihan (1193), light trap, 14–28.iii.2005, 24°24'N, 55°26'E, A. v. Harten, RMNH’05”. ***Paratypes***: 3♀: Idem, 28.iii.–2.iv.2005; 1♀: “United Arab Emirates, Fujairah (1314), light tr[ap], 13–19.iv.2005, 25°08'N, 56°21'E, A. v. Harten, RMNH’05”.

##### Diagnosis.

Ventral half of temple very shiny, mostly smooth to largely finely aciculate (Fig. 273); clypeus very shiny and 0.9 × width of face, intertentorial distance 4 × minimum distance from clypeus to eye (Fig. 272); POL 0.6–0.8 × diameter of posterior ocellus; median carina of frons present; subapical antennal segments somewhat serrate (because of minute subapical protuberances) and non-moniliform (Figs 274, 275); eye in lateral view ca. 2.5 × as wide as temple medially (Fig. 273); vein 1-R1 of fore wing approx. twice distance between apex of marginal cell and apex of wing (Fig. 265); frons with fine median carina; temple rather flat (Figs 271, 273); stemmaticum yellowish brown; length of fore wing approx. 2.5 mm. Similar to *P. glabritemporalis* because of the smooth face and temples, but differs by having temples directly narrowed behind eyes (convex in *P. glabritemporalis*), clypeus with two minute ventral teeth (3), second metasomal suture curved (straight), stemmaticum yellowish brown (largely dark brown), scutellar sulcus narrow (wide) and slender hind femur (widened).

**Figure 264.**
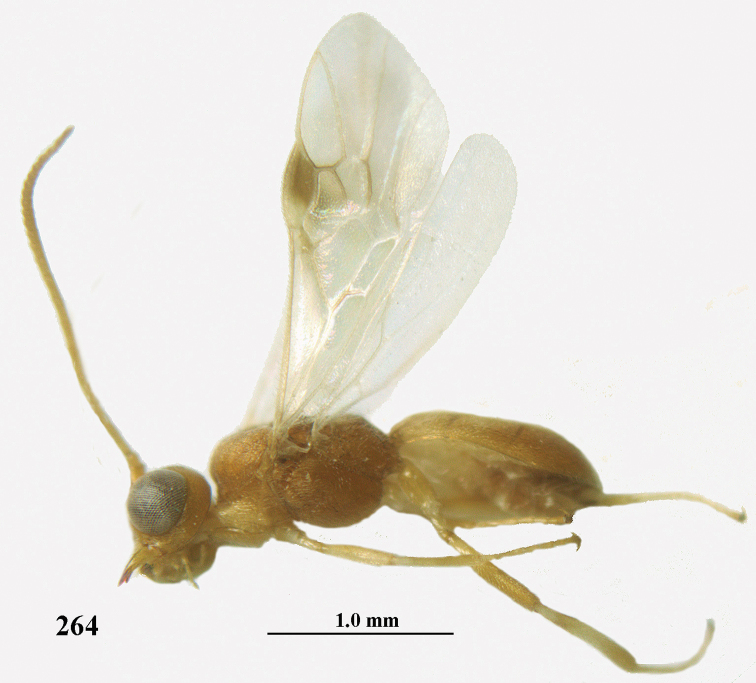
*Phanerotoma micrommata* van Achterberg, sp. nov., ♀, holotype, habitus lateral.

##### Description.

Female, holotype, length of body (excluding ovipositor) 2.8 mm; antenna 2.5 mm; fore wing 2.5 mm; visible part of ovipositor sheath 0.1 mm (entirely setose).

***Head*.** Width 1.5 × median length in anterior view and part of head above eye in lateral view 0.3 × height of eye (Fig. 273); antenna with 23 segments, with small apical spine and as long as fore wing, segments slender and gradually shortened, narrowed apically and segments of apical half with minute subapical protuberance, “pseudo-serrate”, and non-moniliform (Fig. 275), third, fourth and penultimate segments 3.4, 2.8 and 1.5 × longer than wide in lateral view, respectively; area of stemmaticum smooth; OOL: diameter of posterior ocellus: POL = 14: 5: 4; length of eye 2.6 × temple in dorsal view (Fig. 271); frons medially mostly smooth (except for fine median carina), very shiny and laterally finely curved rugulose; vertex superficially rugulose near eyes and remainder mostly smooth and shiny; temple parallel-sided in lateral view, mostly smooth (except some aciculae ventrally) and shiny, rather flat (Fig. 273), in dorsal view directly narrowed behind eyes (Fig. 271); face mostly smooth, very shiny and with short median ridge dorsally; clypeus 0.9 × minimum width of face, smooth and very shiny, with erect setae and medio-ventrally with two obsolescent teeth (Fig. 272); intertentorial distance 4 × minimum distance between clypeus and eye; eye large, strongly convex and in lateral view 2.5 × (measured medially) wider than temple (Fig. 273), in anterior view height nearly equal to minimum width of face; upper condyle of mandible above lower level of eyes (Fig. 272); malar space rugose, shiny and 0.3 × as basal width of mandible; lower tooth of mandible minute, 0.3 × as long as apical tooth (Fig. 270).

**Figures 265–275.**
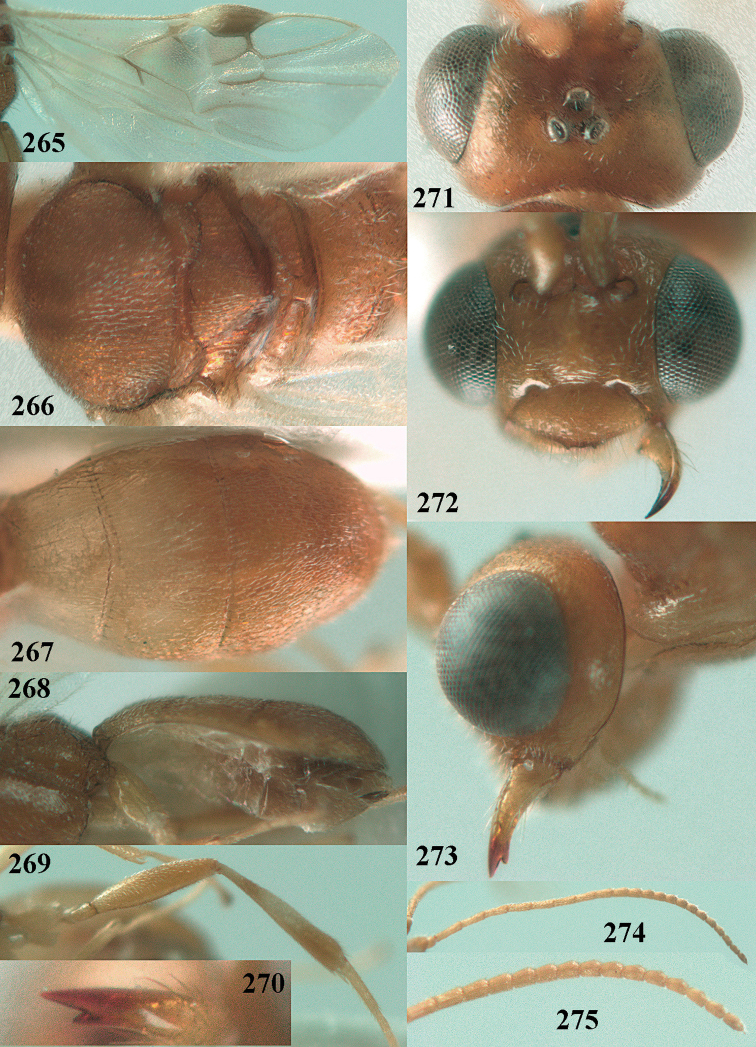
*Phanerotoma micrommata* van Achterberg, sp. nov., ♀, holotype **265** wings **266** mesosoma dorsal **267** first–third metasomal tergites dorsal **268** metasoma lateral **269** hind leg lateral **270** mandible ventral **271** head dorsal **272** head anterior **273** head lateral **274** antenna lateral **275** apical half of antenna lateral.

***Mesosoma*** (Figs 264, 266). Length 1.6 × its width in lateral view; side of pronotum mainly rugose and shiny; mesosternum superficially coriaceous and shiny; mesoscutum finely reticulate-rugulose and rather shiny; notauli absent; scutellar sulcus narrow and with eight short carinae (Fig. 266); scutellum finely longitudinally rugulose and rather shiny; metanotum with short median carina anteriorly, weakly serrate and truncate posteriorly; propodeum nearly smooth anteriorly and remainder finely rugose, without distinct median and transverse carinae, latero-posteriorly not tuberculate. ***Wings*.** Fore wing 2.8 × longer than its maximum width; 1-R1 as long as swollen pterostigma; 1-R1 2.3 × distance between apex of marginal cell and apex of wing; r issued far beyond middle of pterostigma and 0.8 × 3-SR; 2-SR straight and distally converging to posterior margin of pterostigma (Fig. 265); SR1 straight; 2-SR+M rather long because of distinctly postfurcal m-cu; parastigma large; 1-CU1 0.4 × as long as vein 2-CU1 and as long as cu-a; r:3-SR:SR1 = 9:20:130; 2-SR:3-SR:r-m = 26:20:11; r-m vertical; 2-M weakly curved (Fig. 265). Hind wing: M+CU:1-M:1r-m = 26:22:9. ***Legs*.** Hind femur 4.4 × as long as wide and slender (Fig. 269); middle tibia with ivory blister; inner spur of middle tibia 0.4 × its basitarsus; hind coxa mostly smooth and shiny; hind tibia and basitarsus slender (Fig. 269).

***Metasoma*** (Figs 267, 268). Oval in dorsal view, 1.7 × as long as wide and 1.2 × as long as mesosoma; first suture curved; first and second tergites finely and densely longitudinally striate-rugose, rather shiny; third tergite 1.5 × longer than second tergite and laterally curved, in lateral view rather convex (Fig. 268), but apically flat, finely striate-rugulose, rather shiny and medio-posteriorly truncate (Fig. 267), lateral lamella narrow, not protruding latero-apically and medio-apically narrow and truncate; ovipositor sheath medium-sized (Fig. 268), its visible and entirely setose part 0.05 × as long as fore wing and 0.11 × metasomal carapace; hypopygium rather obtuse apically, without short apical triangle or apical spine.

***Colour*.** Brownish yellow (including stemmaticum); palpi, mandible (except reddish brown apex), clypeus, malar space, tegulae, pronotum, propleuron, legs (but hind femur and tibia brownish apically), first and second metasomal tergites and metasoma ventrally pale yellowish or ivory; ovipositor sheath brown; pterostigma rather dark brown, but basally and narrowly apically pale yellowish (Fig. 265); wing membrane subhyaline but below pterostigma slightly infuscate; parastigma, veins 1-M, 2-CU1 and m-cu of fore wing pale yellowish and veins r, 1-CU1, cu-a, 2-SR, 3-SR and 2-M brown.

##### Male.

Unknown.

##### Biology.

Unknown.

##### Distribution.

United Arabian Emirates.

##### Etymology.

Named after its smaller ocelli (*mikrommatos* is Greek for small-eyed).

#### Phanerotoma ocularis

Taxon classificationAnimaliaHymenopteraBraconidae

Kohl, 1906

43B368FE-FDE3-5C6B-9702-C4B8957C3599

Figs 276–279 , 280–290 , 318 , 320

Phanerotoma ocularis Kohl, 1906: 124–125; Shenefelt 1973: 920–921.
Phanerotoma hispanica var. desertorum Hedwig, 1957: 112; van Achterberg 1990: 50 (synonymised with P. ocularis Kohl).

##### Type material.

***Lectotype*** of *P. ocularis*, ♀ (NMW): “[**Yemen**,] Sokotra, 2.99, leg. O. Simony”, “*Ph. ocularis* Kohl, Type, J, det. Kohl”. ***Lectotype*** of *P. desertorum*, ♀ (Staatliches Museum Stuttgart): “Iran, Belutschistan, Iranshar, 800 m, 110.III.1954, Richter u. Schäuffele”. “*Phanerotoma hispanica* Kok. v. *desertorum* Hedwig, Holotyp.”, “Holotypus”, “Typus”.

##### Additional material.

From **United Arab Emirates** (Fujairah; NARC near Sweihan; al-Ajban; Sharjah Desert Park; Wadi Bih dam) and **Yemen** (Al Kadan; Al Kowd; Ta’izz; Hamman’Ali; Mayfa’ah).

**Figures 276–279.**
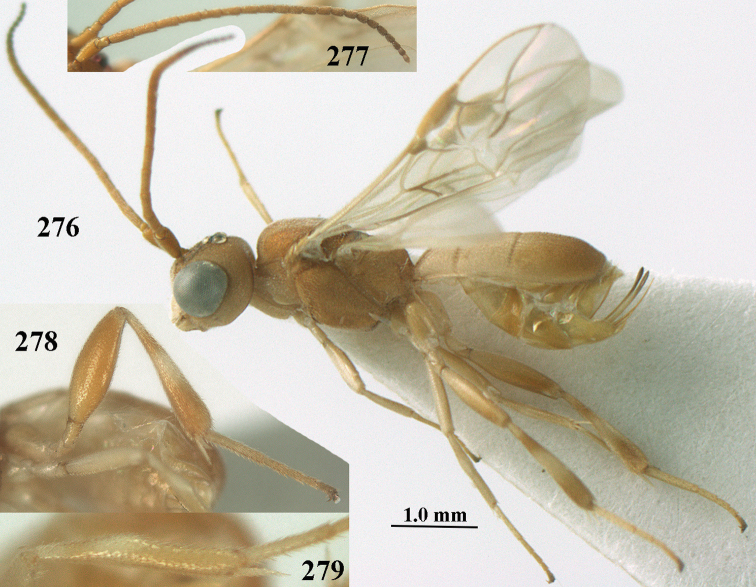
*Phanerotoma ocularis* Kohl, ♀, UAE (but **277–279** of ♂, Yemen) **276** habitus lateral **277** antenna lateral **278** hind femur and tibia lateral **279** middle tibia lateral.

##### Diagnosis.

**Figures 280–290.**
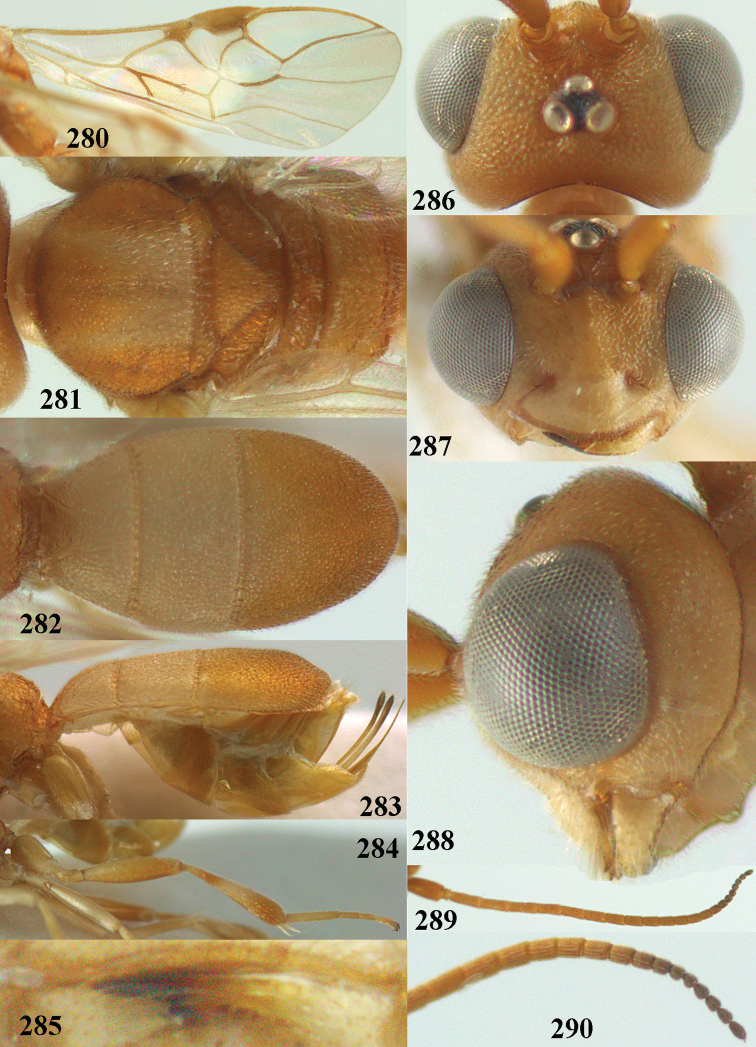
*Phanerotoma ocularis* Kohl, ♀, UAE**280** fore wing **281** mesosoma dorsal **282** first–third metasomal tergites dorsal **283** metasoma lateral **284** hind leg lateral **285** mandible ventral **286** head dorsal **287** head anterior **288** head lateral **289** antenna lateral **290** apical half of antenna lateral.

Area above eye of ♀ in lateral view ca. 0.3 × height of eye (Fig. 288); ocelli large (Fig. 286) and OOL 1.6–2.6 × diameter of posterior ocellus; POL 0.4–0.6 × posterior ocellus; scutellum densely rugulose; posterior ocelli somewhat larger than anterior ocellus (Fig. 286); length of eye in dorsal view 1.5–2.3 × length of temple (Fig. 286); penultimate antennal segments of ♀ usually somewhat longer than wide and gradually shortened (Figs 289, 290); vertex variable, irregularly or regularly and finely rugulose or rugulae largely absent; scapus variable, may just surpassing level of posterior ocelli; second tergite laterally brownish, often distinctly darker than ivory middle of tergite; medio-ventral tooth of clypeus absent or obsolescent (Fig. 287); vein 1-R1 4–5 × distance from vein 1-R1 to wing apex (Fig. 280; 5 × in lectotype of *P. ocularis*); blister of middle tibia distinct; third tergite laterally distinctly convex and apically subtruncate (Fig. 282), only lamella somewhat emarginate; blister of middle tibia ivory; anteriorly vein 2-SR partly subparallel to posterior margin of pterostigma; median length of third tergite ca. 1.5 × posterior width of lamella of tergite; propodeum medio-posteriorly yellowish brown; side of scutellum partly or completely yellowish brown (= *P. ornatulopsis* de Saeger, 1948, sensu Hedqvist, 1965 p.p.).

##### Distribution.

Congo (head in dorsal view ca. twice wider than its median length), Cabo Verde (2.2–2.4 ×), Somalia, Senegal, Kenya, Tanzania, *United Arab Emirates, Yemen (mainland and Sokotra).

##### Notes.

Zettel (1992) re-instated *P. flavitestacea* Fischer, 1959, as valid species after it was synonymised by van Achterberg (1990) with *P. ocularis* Kohl. Manuela Vizek and Dominique Zimmermann (Naturhistorisch Museum Wien) kindly supplied additional information on the types of both taxa. Zettel (1992) did not give any argument for this action but after seeing many specimens from the Arabian Peninsula I agree that *P. ocularis* is very similar, but can be separated from *P. flavitestacea*. Both have the enlarged eyes and ocelli combined with the square tenth segment from the apex of the female antenna. The Southwest Palaearctic *P. flavitestacea* Fischer (described from Croatia and introduced in U.S.A. (California, Florida) has the lamella of third tergite narrow, third tergite wide apically and rather flattened, scutellum superficially coriaceous, POL 0.6–0.7 × width of posterior ocellus; scutellum often partly darkened, eye in lateral view 1.4–1.5 × maximum width of temple. The Oriental *P. orientalis* Szépligeti, 1902, is very similar to *P. flavitestacea*, but differs by having the lamella of third tergite wide, third tergite narrowed apically and distinctly convex and scutellum punctulate-coriaceous.

*Phanerotoma ornatulopsis* De Saeger, 1942 (type from Congo examined) is very close to *P. ocularis* Kohl, but *P. ornatulopsis* differs by the distinctly transversely rugose vertex behind the stemmaticum, the smaller ocelli, the coarsely rugose frons and the gradually flattened third tergite. Van Achterberg and Polaszek (1996) considered the differences between *P. leucobasis* (as *P. ocularis*) and *P. ornatulopsis* listed by van Achterberg (1990) too variable to be useful in separation of both species and, therefore, *P. ornatulopsis* was synonymized, but now I refrain from including it as a synonym till more is clear about the value of the listed differences.

#### Phanerotoma permixtellae

Taxon classificationAnimaliaHymenopteraBraconidae

Fischer, 1968

7D748DBF-374D-50BD-8708-5CC85B8E32BF

Figs 291–293 , 294–304

Phanerotoma permixtellae Fischer, (April) 1968a: 107–109; Shenefelt 1973: 922.
Phanerotoma olearia Fischer, (Dec.) 1968b: 331–333; Shenefelt 1973: 921 (synonymized with P. permixtellae Fischer by van Achterberg (1990)).
Phanerotoma (Bracotritoma) permixtellae ; van Achterberg, 1990: 53–54.

##### Type material.

***Paratype*** of *P. permixtellae*, ♀, (NMW): “*Phanerotoma permixtellae* n. sp., ♀, det. Fischer/ Paratype” (according to the original description the type locality is **Syria**, Lattaquié); ***holotype*** of *P. olearia*, (MG): “*Phanerotoma olearia* n. sp., det. Fischer / Holotype”, “C.I.L.B., Ex.: S/ olivier, Syrie”; 1♀, paratype of *P. olearia*, (NMW): “Syrien, an Ölbaum, lg Katlab”, “*Phanerotoma olearia* n. sp., ♀, det. Fischer/Paratype”.

##### Additional material.

From **Yemen** (Al Kowd; Ta’izz; Seyun; Al Kadan; Al Mukalla; Al Lahima).

**Figures 291–293.**
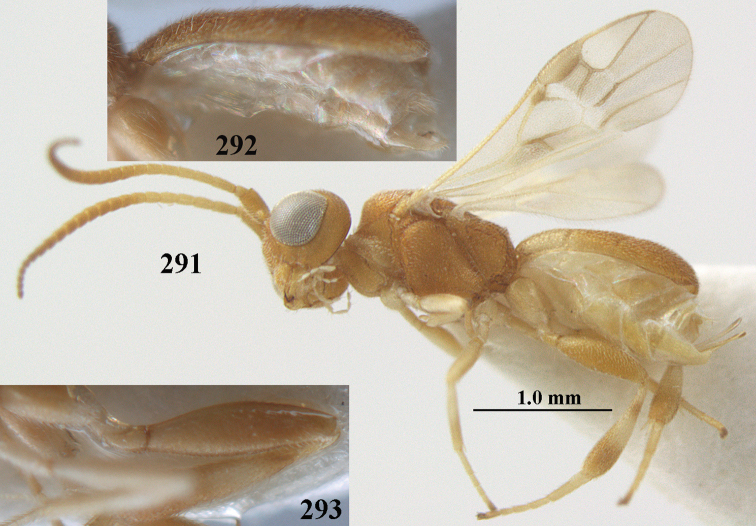
*Phanerotoma permixtellae* Fischer. ♀ (but **292, 293** of ♂), Yemen **291** habitus lateral **292** metasoma lateral **293** hind femur and tibia lateral.

##### Diagnosis.

**Figures 294–304.**
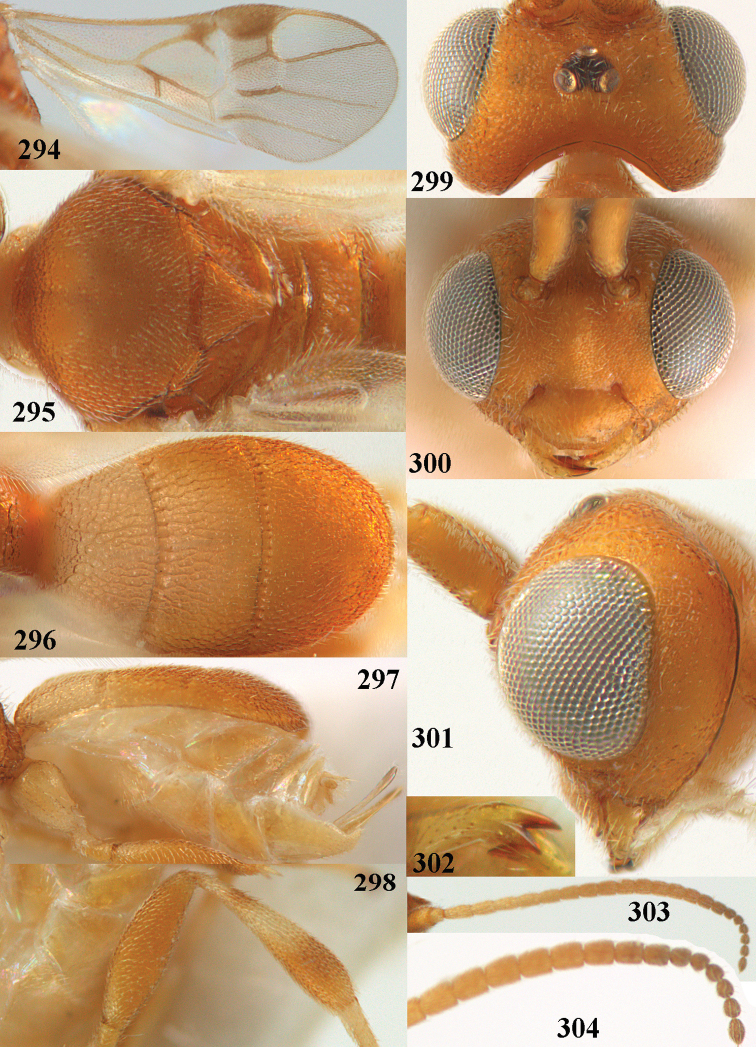
*Phanerotoma permixtellae* Fischer, ♀, Yemen **294** fore wing **295** mesosoma dorsal **296** first–third metasomal tergites dorsal **297** metasoma lateral **298** hind femur and tibia lateral **299** head dorsal **300** head anterior **301** head lateral **302** mandible ventral **303** antenna lateral **304** apical half of antenna lateral.

Antenna of ♀ submedially widened (Figs 291, 303) and antennal segments of ♀ stout and cylindrical, but six apical segments narrowed basally and moniliform (Fig. 304); inner tooth of mandible 0.6–0.9 × apical tooth (Fig. 302); clypeus rather narrow (Fig. 300); vein 1-M yellow; parastigma and most of pterostigma brown; vein m-cu of fore wing distinctly postfurcal; vein r of fore wing vertical (Fig. 294); hind femur and tibia rather swollen (Fig. 298); third metasomal tergite rather matt and densely sculptured, slightly convex medially (Fig. 296) and obtuse posteriorly in lateral view (Fig. 297); ovipositor sheath somewhat widened apically (Fig. 297); hypopygium of ♀ with short triangular protuberance (Fig. 297); hind femur of both sexes similar (Figs 293, 298).

##### Distribution.

Greece, Iran, Israel, Syria, *Yemen.

##### Biology.

Reared from *Cacochroa permixtella* (H.-S.) (Oecophoridae) on olive.

#### Phanerotoma robusta

Taxon classificationAnimaliaHymenopteraBraconidae

Zettel, 1988

9ABCAD02-A482-5DE0-94C1-3A8D3314C16E

Figs 305 , 306–316 , 317 , 319

Phanerotoma robusta Zettel, 1988: 199–201.
Phanerotoma (Phanerotoma) robusta ; van Achterberg, 1990: 57; Edmardash and Gadallah 2019: 369–372.

##### Additional material.

1♀: “**United Arab Emirates**, al-Ajban (6424–6425), light tr[ap], 26.ii.–27.iii.2006, 24°36'N, 55°01'E, A. v. Harten, RMNH’07”.

##### Comparative diagnosis.

Similar to *P. ocularis* Kohl, but unique because of the long bristles on the fore tarsus (Fig. 319), comparatively small head (Fig. 317) and its large size; inner tooth of mandible 0.2 × as long as apical tooth (Fig. 311); length of malar space 0.7 × basal width of mandible; legs elongate; width of head 0.8–0.9 × maximum width of mesoscutum (Fig. 317); hypopygium with small apical up slanted triangle (Fig. 309); length of fore wing 5.4–5.5 mm and of body 7.0–7.3 mm; wing membrane hyaline.

**Figure 305.**
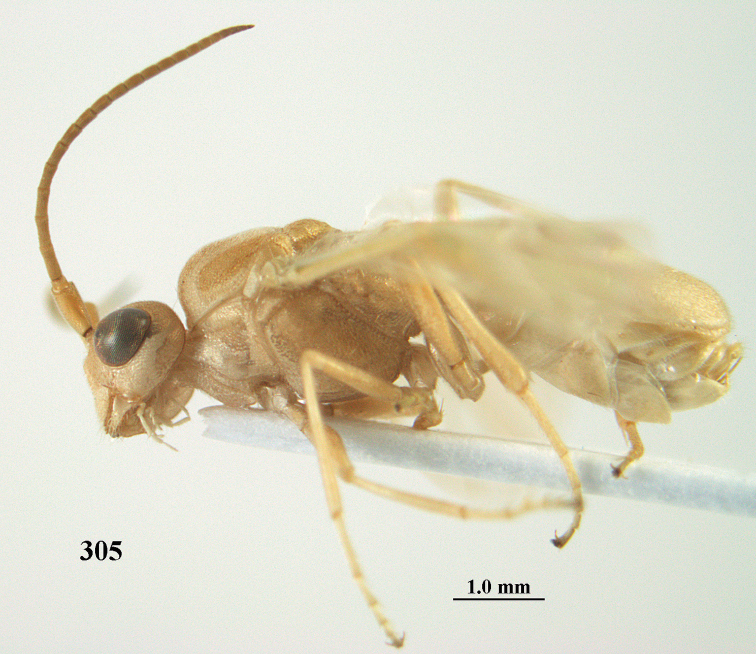
*Phanerotoma robusta* Zettel, ♀, UAE, habitus, lateral.

##### Distribution.

Kuwait (holotype; NMW), Egypt (Sinai), *United Arab Emirates. Zettel (1988) reported the holotype to be from Dasmat (Saudi Arabia), however Dasma(t) is situated in Kuwait.

##### Biology.

Unknown.

**Figures 306–316.**
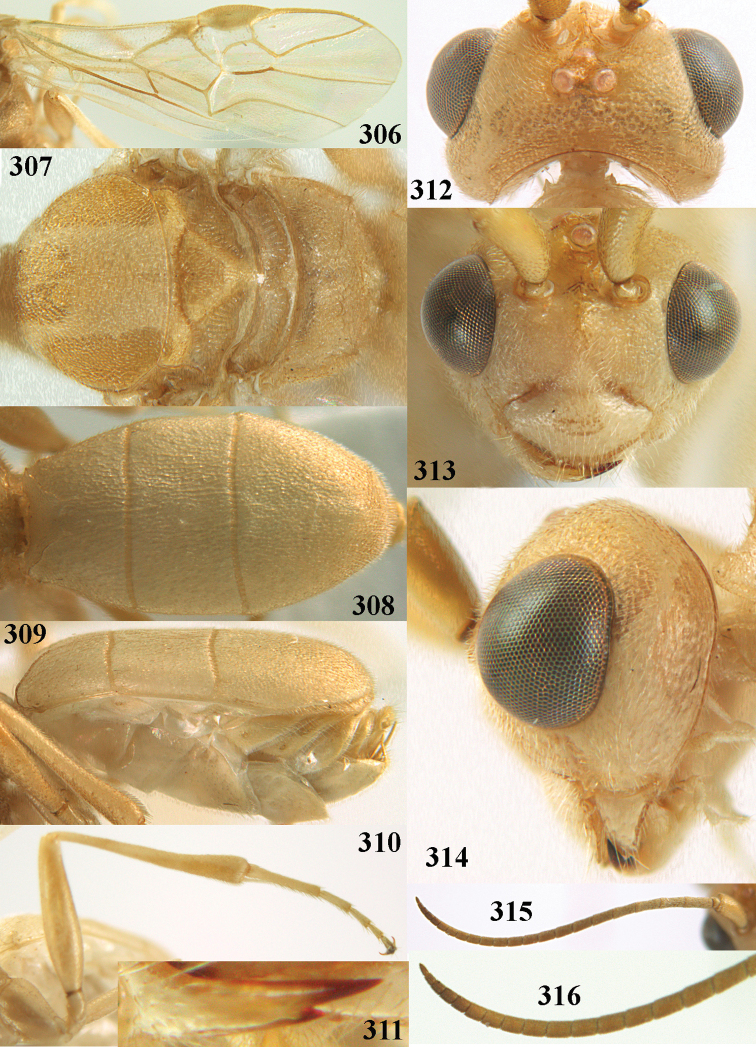
*Phanerotoma robusta* Zettel, ♀, UAE**306** fore wing **307** mesosoma dorsal **308** first–third metasomal tergites dorsal **309** metasoma lateral **310** hind femur and tibia lateral **311** mandible ventral **312** head dorsal **313** head anterior **314** head lateral **315** antenna lateral **316** apical half of antenna lateral.

**Figures 317–320.**
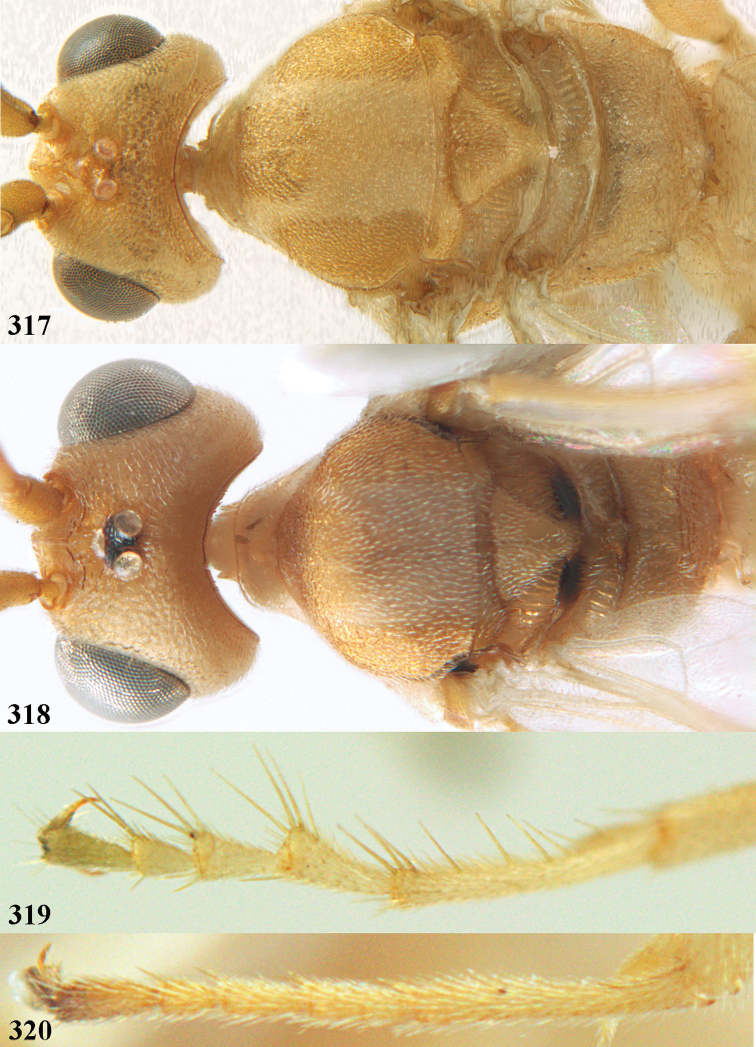
**317, 319***Phanerotoma robusta* Zettel, ♀, UAE**318, 320***Phanerotoma ocularis* Kohl, ♀, Yemen **317, 318** head and mesosoma dorsal **319, 320** fore tarsus lateral.

#### Phanerotoma sculptilis sp. nov.

Taxon classificationAnimaliaHymenopteraBraconidae

3058D020-7C5C-52F9-B22A-1125D31BAD78

http://zoobank.org/501AB171-D62B-44B0-BC9F-B62083F94F7C

Figs 321–324 , 325–335

##### Type material.

***Holotype***, ♀ (RMNH), “**Yemen**: Ta’izz (2910), light trap, 5.i.–2.ii.1998, A. van Harten, RMNH’98”. ***Paratypes***: 1♀: Same data as holotype; 1♀: Idem, vi.2002, A. van Harten & A.R. Al Yarimi; 1♀, 1♂: “Yemen: Al Kowd (7157), vi.2002, light trap, A. v. Harten & S. Al Haruri, RMNH’03”; 2♀: “Yemen (6158), Al Lahima, 17.ix.–14.xi.2001, Mal[aise] trap, A. v. Harten, RMNH’02”; 2♀: “Yemen (6876), Seyun, vi.2002, light trap, A. v. Harten, RMNH’03”; 1♀: “**United Arab Emirates**, al-Ajban (6418), Malaise & light tr[ap], 7–28.xii.2006, 24°36'N, 55°01'E, A. v. Harten, RMNH’07”.

**Figures 321–324.**
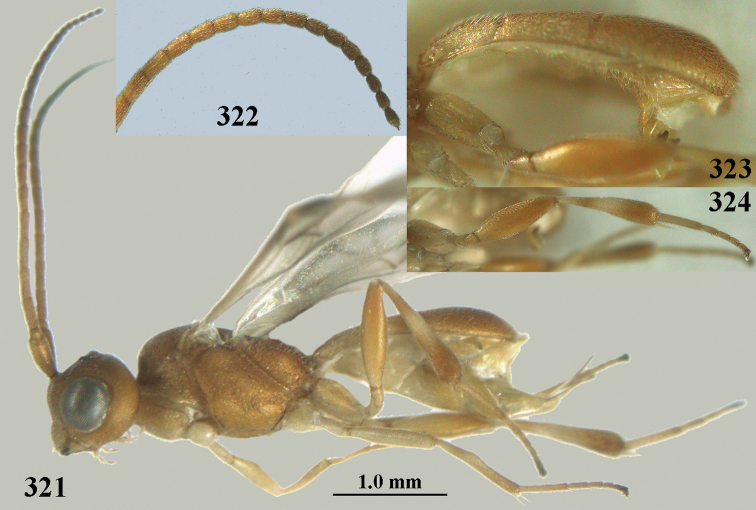
*Phanerotoma sculptilis* van Achterberg, sp. nov., ♀, holotype (but **322–324** of ♂, paratype) **321** habitus lateral **322** apical half of antenna lateral **323** metasoma lateral **324** hind leg lateral.

##### Diagnosis.

**Figures 325–335.**
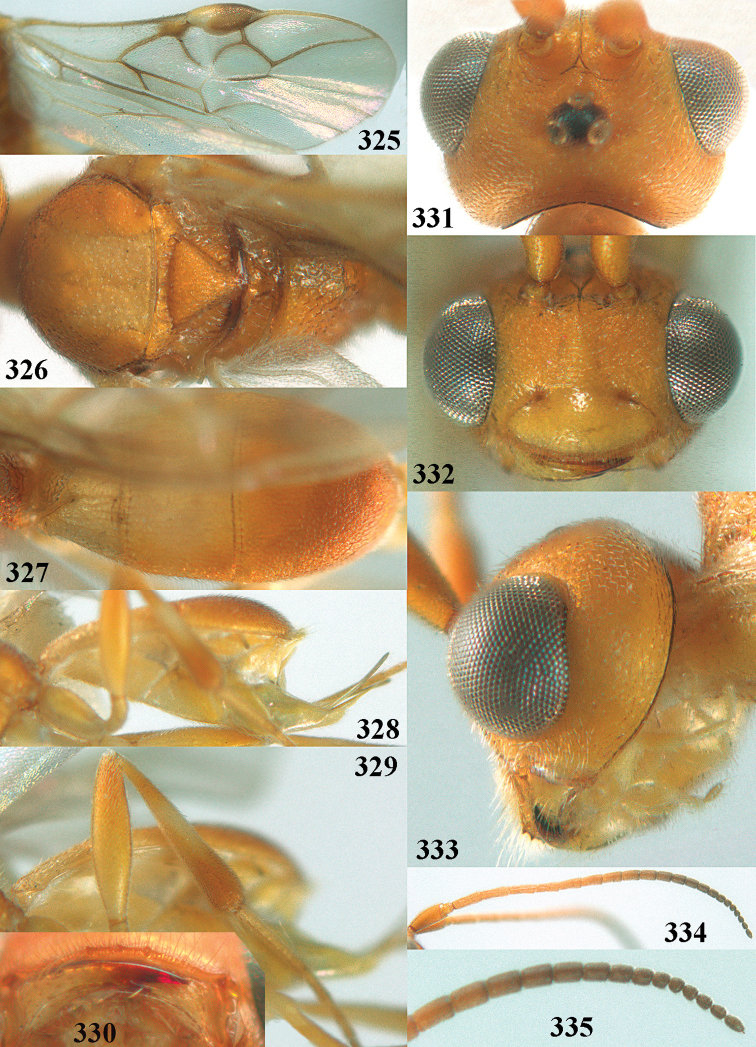
*Phanerotoma sculptilis* van Achterberg, sp. nov., ♀, holotype **325** wings **326** mesosoma dorsal **327** first–third metasomal tergites dorsal **328** metasoma lateral **329** hind leg lateral **330** mandible ventral **331** head dorsal **332** head anterior **333** head lateral **334** antenna lateral **335** apical half of antenna lateral.

Third tergite 1.5–1.7 × as long as second tergite, curved laterally, densely sculptured, rather dull and convex (Fig. 327), its posterior lamella wide and truncate medio-apically; segments of apical half of antenna cylindrical in lateral view, without small subapical protuberances and subapical segments shorter, moniliform or submoniliform (Fig. 335); fourth antennal segment ca. 3 × as long as wide; temple widened dorsally (Fig. 333), matt to slightly shiny, granulate near eye and longitudinally rugulose posteriorly; face nearly entirely densely sculptured and shiny; frons rugose and anteriorly with median carina; clypeus ca. as wide as face, intertentorial distance ca. 4.0 × minimum distance between clypeus and eye ventrally, and very shiny (Fig. 332); vertex with satin sheen; vein r of fore wing non-linear with vein 3-SR (Fig. 325); OOL aciculate; vertex rather shiny (Fig. 331); vein 1-CU1 of fore wing medium-sized and vein cu-a moderately oblique (vein cu-a approx. as long as vein 1-CU1; Fig. 325); hind femur of ♀ and tarsal claws less slender than in *P. ejuncida* (Fig. 329). Easily confused with finely sculptured specimens of *P. glabritemporalis*, but the temple is widened dorsally in lateral view (parallel-sided in *P. glabritemporalis*), matt or nearly so (rather shiny), frons superficially granulate and with median carina (Fig. 331; aciculate and without median carina).

##### Description.

Female, holotype, length of body (excluding ovipositor and hypopygium) 4.1 mm; antenna 3.8 mm; of fore wing 3.4 mm; visible part of ovipositor sheath 0.55 mm (only apically setose).

***Head*.** Width 1.5 × median length in anterior view and part of head above eye in lateral view 0.4 × height of eye (Fig. 333); antenna with 23 segments and 1.1 × longer than fore wing, segments slender and gradually shortened, segments of apical half without minute subapical protuberances and cylindrical, with apical spine, six apical segments moniliform and narrowed basally (Figs 334, 335), third, fourth and penultimate segments 3.2, 3.0 and 1.3 × longer than wide in lateral view, respectively; area of stemmaticum granulate; OOL: diameter of posterior ocellus: POL = 14: 5: 4; length of eye 1.9 × temple in dorsal view (Fig. 331); frons largely granulate, shiny and with median carina, but V-shaped dorsally (Figs 331, 332); vertex transversely rugose and with satin sheen; temple granulate near eye and longitudinally rugose posteriorly, convex and with satin sheen; clypeus approx. as wide as face (intertentorial distance ca. 3.5 × minimum distance between clypeus and eye ventrally), convex, mostly smooth and shiny (Fig. 332); face rather shiny and distinctly rugose, with median carina dorsally; clypeus with 3 obsolescent teeth medio-ventrally (Fig. 330); eye large, strongly convex and in lateral view 1.9 × (measured medially) temple and widened dorsally (Fig. 333), in anterior view its height 0.8 × minimum width of face; upper condyle of mandible near lower level of eyes (Fig. 332); malar space aciculate, shiny and 0.3 × as basal width of mandible; lower tooth of mandible moderately slender and 0.4 × as long as apical tooth (Fig. 330).

***Mesosoma*** (Figs 321, 326). Length 1.5 × its width in lateral view; side of pronotum distinctly rugose and shiny; mesosternum spaced punctulate and shiny; seven finely reticulate-rugose and rather shiny; notauli absent; scutellar sulcus wide, with seven carinae (Fig. 326); scutellum widely triangular, densely finely granulate-rugose (nearly up to posterior margin), convex and rather shiny; metanotum with complete median carina; propodeum coarsely reticulate-rugose, without distinct median and transverse carinae and latero-posteriorly not tuberculate. ***Wings*.** Fore wing 3.1 × longer than its maximum width; length of 1-R1 1.6 × as long as pterostigma; r issued rather far beyond middle of pterostigma and 0.3 × 3-SR; distance between 1-R1 and wing apex 0.2 × 1-R1; 1-SR+M and 2-SR curved and distally subparallel with posterior margin of pterostigma (Fig. 325); SR1 curved; 2-SR+M present because of narrowly postfurcal m-cu; parastigma large; 1-CU1 0.3 × as long as vein 2-CU1, cu-a strongly inclivous and as long as 1-CU1; r:3-SR:SR1 = 5:15:46; 2-SR:3-SR:r-m = 20:15:6; r-m reclivous; 2-M slightly curved (Fig. 325). Hind wing: M+CU:1-M:1r-m = 21:18:10; cu-a narrow. ***Legs*.** Hind femur widened medially and 3.9 × as long as wide (Fig. 329); middle tibia with ivory blister; inner spur of middle tibia 0.6 × its basitarsus; hind coxa largely granulate dorsally and with satin sheen; hind basitarsus slender (Fig. 329); tarsal claws moderately slender.

***Metasoma*** (Figs 327, 328). Oval in dorsal view, twice as long as wide and 1.3 × as long as mesosoma; first and second tergites finely and densely longitudinally rugose; third tergite 1.5 × longer than second tergite and laterally curved (Fig. 327), in lateral view rather convex (Fig. 328), densely reticulate-rugulose and medio-posteriorly truncate, lateral lamella narrow, not protruding latero-apically and medio-apically truncate and wide; ovipositor sheath narrow and parallel-sided, its visible part 0.16 × as long as fore wing and 0.31 × metasomal carapace and only apically some long and erect setae; hypopygium apically robust and short up curved triangle and no spine.

***Colour*.** Yellowish brown; palpi, mandible (except dark brown teeth), clypeus, malar space, tegulae (but humeral plate partly brown), prothorax, mesoscutum medially, legs (but apical half of middle tibia, hind femur apically and ventrally, and hind tibia apically and basally brownish), first and second metasomal tergites and metasoma ventrally largely pale yellow or ivory; apical third of antenna and ovipositor sheath largely brown; stemmaticum and pterostigma dark brown, but basally and narrowly apically pale yellowish (Fig. 325); wing membrane subhyaline but below dark part of pterostigma slightly infuscate; parastigma (but anteriorly pale yellowish) and vein 1-M largely brown; veins 1- & 2-CU1, r and 3-SR of fore wing rather dark brown.

##### Male.

Similar to female, but apical antennal segments longer (Fig. 322), hind femur moderately widened (3.3 × as long as wide; Fig. 324); vein 1-M yellowish; pterostigma largely pale yellowish or partly darkened; third tergite brown and 1.5 × as long as second tergite and often truncate in lateral view (Fig. 323).

##### Variations.

Length of fore wing 2.6–3.6 mm; third tergite 1.5–1.7 × longer than second tergite; parastigma and vein 1-M of fore wing varies from pale yellow to brownish; vein cu-a of fore wing 0.8–1.3 × as long as vein 1-CU1; length of 1-R1 1.4–1.6 × as long as pterostigma; r 0.2–0.3 × 3-SR.

##### Biology.

Unknown.

##### Distribution.

Yemen, United Arab Emirates.

##### Etymology.

Named after the sculptured temples; *sculptilis* is Latin for carved.

#### Phanerotoma signifera sp. nov.

Taxon classificationAnimaliaHymenopteraBraconidae

4A3B4F98-A006-59BC-8002-13030E7DAAC0

http://zoobank.org/65F97859-F85A-48FD-A3CC-AE2953F89378

Figs 336 , 337–346

##### Type material.

***Holotype***, ♀ (RMNH), “**Yemen**: Al Kowd (4055), ix.1999, light trap, A. v. Harten & S. Al Haruri, RMNH’00”.

##### Diagnosis.

Distance between apex of marginal cell and apex of fore wing 0.3–0.4 × vein 1-R1 (Fig. 337) and vertex finely rugulose; antenna of ♀ with six apical moniliform segments and widened subapically (Fig. 346); ocelli small (POL ca. 1.4 × width of posterior ocellus; Fig. 343); head 1.4 × wider than high medially in anterior view (Fig. 344); head distinctly emarginate medio-posteriorly in dorsal view (Fig. 343); inner tooth of mandible small, 0.3 × apical tooth; second submarginal cell of fore wing small (Fig. 337); anterior half of vein 1-M yellow; vein r of fore wing ca. 0.7 × vein 3-SR (Fig. 337); pterostigma conspicuously dark and large compared to weakly pigmented venation (Fig. 337); third tergite of metasoma flattened in lateral view (Fig. 340). Close to *P. lepta* but differs by the different shape of the second submarginal cell of the fore wing, of the pterostigma and of the head in anterior view and dorsal view, smaller ocelli, longer vein r of the fore wing and conspicuous pterostigma.

**Figure 336.**
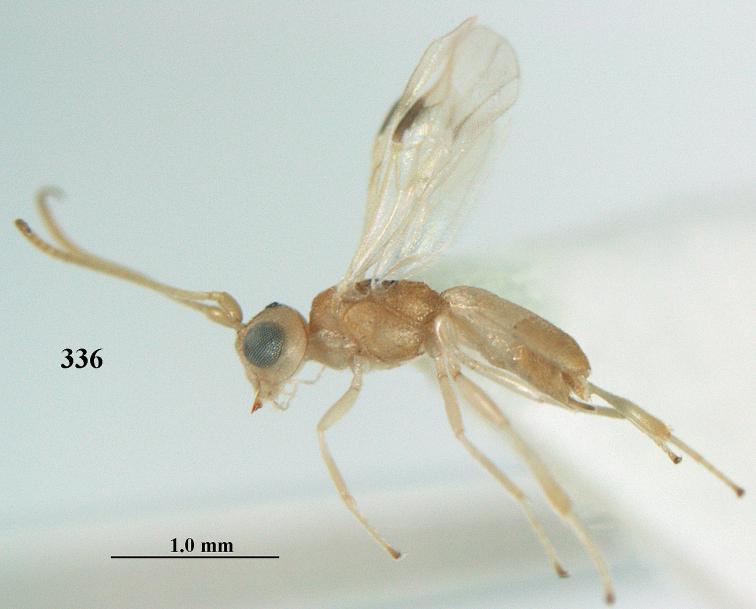
*Phanerotoma signifera* van Achterberg, sp. nov., ♀, holotype, habitus lateral.

##### Description.

Female, holotype, length of body (excluding ovipositor) 2.1 mm; antenna 1.9 mm; fore wing 1.8 mm; visible part of ovipositor sheath 0.15 mm (only apically erect setose).

***Head*.** Width 1.4 × median length in anterior view and part of head above eye in lateral view 0.3 × height of eye (Fig. 345) and emarginate medio-posteriorly (Fig. 343); antenna widened subapically, with 23 segments and slightly shorter than fore wing, six apical antennal segments suddenly (compared to more basal segments) small and moniliform (Fig. 342) and apical segment with tiny spine, third, fourth and penultimate segments 2.5, 2.7 and 1.2 × longer than wide in lateral view, respectively; area of stemmaticum punctulate but mostly smooth posteriorly; OOL: diameter of posterior ocellus: POL = 72: 18: 23; length of eye 2.1 × temple in dorsal view (Fig. 343); frons smooth near antennal sockets, aciculate medially, rugulose laterally and without median carina; vertex superficially coriaceous and rather shiny, but posteriorly rugulose; temple superficially coriaceous and with satin sheen; face superficially finely rugulose and with small median bump and with satin sheen; clypeus smooth and shiny and 0.8 × minimum width of face, intertentorial distance 2.5 × minimum width between clypeus and eye, long erect setose and with three obsolescent teeth medio-ventrally (Fig. 344); eye medium-sized, strongly convex and in lateral view 1.4 × wider than temple (measured medially; Fig. 345), in anterior view its height 0.7 × minimum width of face; upper condyle of mandible below lower level of eyes (Fig. 344); malar space rugulose-coriaceous, with satin sheen and 0.7 × as long as basal width of mandible; lower tooth of mandible 0.3 × as long as apical tooth (Fig. 345).

**Figures 337–346.**
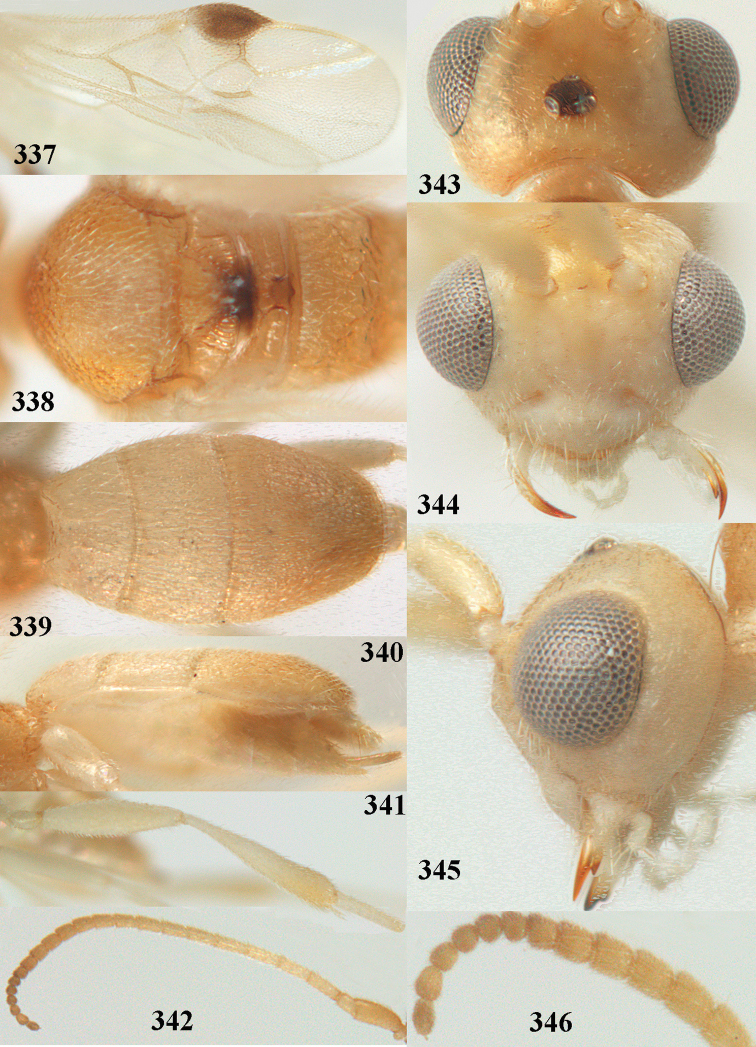
*Phanerotoma signifera* van Achterberg, sp. nov., ♀, holotype **337** wings **338** mesosoma dorsal **339** first–third metasomal tergites dorsal **340** metasoma lateral **341** hind leg lateral **342** antenna lateral **343** head dorsal **344** head anterior **345** head lateral **346** apical third of antenna lateral.

***Mesosoma*** (Figs 336, 338). Length 1.4 × its width in lateral view; side of pronotum crenulate medially and posteriorly, punctate dorsally and mostly smooth ventrally; propleuron posteriorly distinctly convex; mesosternum granulate and rather matt; mesoscutum densely and finely reticulate-rugulose; scutellum rugulose-punctate (but smooth posteriorly) and rather matt; notauli not indicated; scutellar sulcus wide, with three carinae (Fig. 338); metanotum without median carina anteriorly and with minute tooth medio-posteriorly; propodeum rugulose anteriorly and remainder rugose, without transverse or median carina, and latero-posteriorly slightly tuberculate. ***Wings*.** Fore wing 2.7 × longer than its maximum width; pterostigma wide; length of 1-R1 1.1 × as long as pterostigma; distance between wing apex and marginal cell apex 0.35 × length of vein 1-R1; r issued far beyond middle of pterostigma and 0.7 × 3-SR; 2-SR straight and distally slightly converging to posterior margin of pterostigma (Fig. 337); SR1 straight; 2-SR+M indistinct, m-cu sub-interstitial; parastigma large; 1-CU1 0.45 × as long as vein 2-CU1, cu-a as long as 1-CU1; r:3-SR:SR1 = 9:9:51; 2-SR:3-SR:r-m = 21:9:8; r-m reclivous; 2-M slightly curved (Fig. 337). Hind wing: M+CU:1-M:1r-m = 18:16:5. ***Legs*.** Hind femur with satin sheen, 4.1 × as long as wide and slightly widened submedially (Fig. 341); blister of middle tibia obsolescent; inner spur of middle tibia 0.4 × its basitarsus; hind coxa mostly smooth and shiny.

***Metasoma*** (Figs 339, 340). Elliptical in dorsal view, 1.8 × longer than wide and 1.3 × as long as mesosoma; first and second tergites, densely and finely longitudinally rugose; second metasomal suture medium-sized; third tergite 1.5 × longer than second tergite and laterally nearly straight and apically widely truncate (Fig. 339), in lateral view rather flat, finely rugulose-coriaceous and with satin sheen (Fig. 340), lateral lamella medium-sized, wider and not protruding latero-apically and medio-apically truncate and rather wide; ovipositor sheath parallel-sided, apically narrow, its visible part 0.08 × as long as fore wing and 0.15 × metasomal carapace and only apically with long erect setae; hypopygium with medium-sized straight apical triangle (Fig. 340), apically without spine and densely setose.

***Colour*.** Pale brownish yellow (including ovipositor sheath); basal half of antenna, face, clypeus, tegulae, legs, palpi, pronotum, propleuron, first and second tergites white or nearly so; apical half of antenna yellowish brown; apex of ovipositor sheath brown; stemmaticum, pterostigma (but basally and ventrally pale) and apex of scutellum dark brown; parastigma and veins ivory or colourless; apical half of third tergite and apical half of metasoma ventrally rather darkened; wing membrane slightly brownish below pterostigma.

##### Male.

Unknown.

##### Biology.

Unknown.

##### Distribution.

Yemen.

##### Etymology.

The name *signifera* refers to the conspicuously dark pterostigma (*signum* is Latin for mark and -*fera* is a Latin suffix meaning to bear, carry or have).

#### Phanerotoma spuriserrata sp. nov.

Taxon classificationAnimaliaHymenopteraBraconidae

6B178ABF-6522-5E13-8CD3-A8FB24140F3F

http://zoobank.org/BAC9B069-0809-48AA-A832-8EF1E622EA15

Figs 347 , 348–358

##### Type material.

***Holotype***, ♀ (RMNH), ♀, “**Yemen** (no. 2910), Ta’izz, light trap, 5.i.–2.ii.1998, A. van Harten, RMNH’98”. ***Paratypes***: 9♀, 1♂: Same data as holotype; 1♂: Idem, but 1–3.iv.1998; 3♀: Idem, but 26–28.vii.1999; 1♀, 1♂: Idem, but 3–24.i.1999; 1♀: Idem, but x.1999; 1♀: Idem, but xii.1999; 1♀, 2♂: Idem, but v.2002; 1♂: “Yemen (6158), Al Lahima, 17.ix.–14.xi.2001, Mal[aise] trap, A. v. Harten, RMNH’02”.

**Figure 347.**
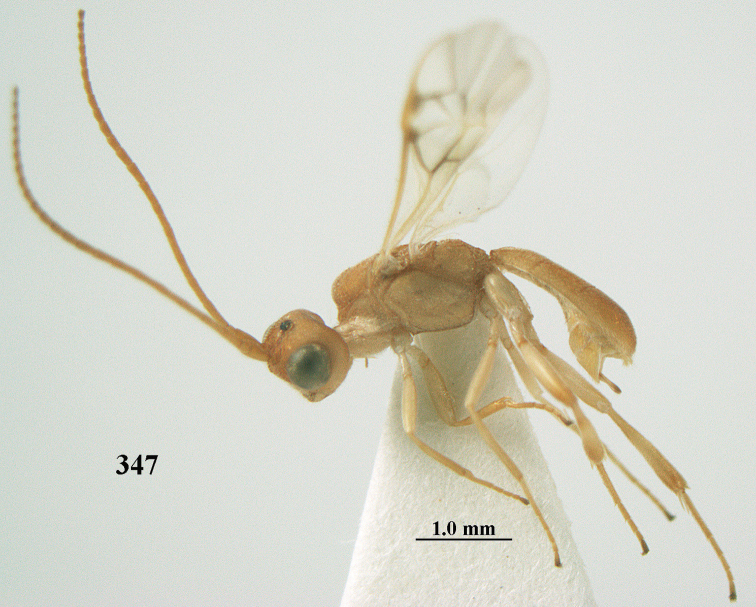
*Phanerotoma spuriserrata* van Achterberg, sp. nov., ♀, holotype, habitus lateral.

##### Diagnosis.

**Figures 348–358.**
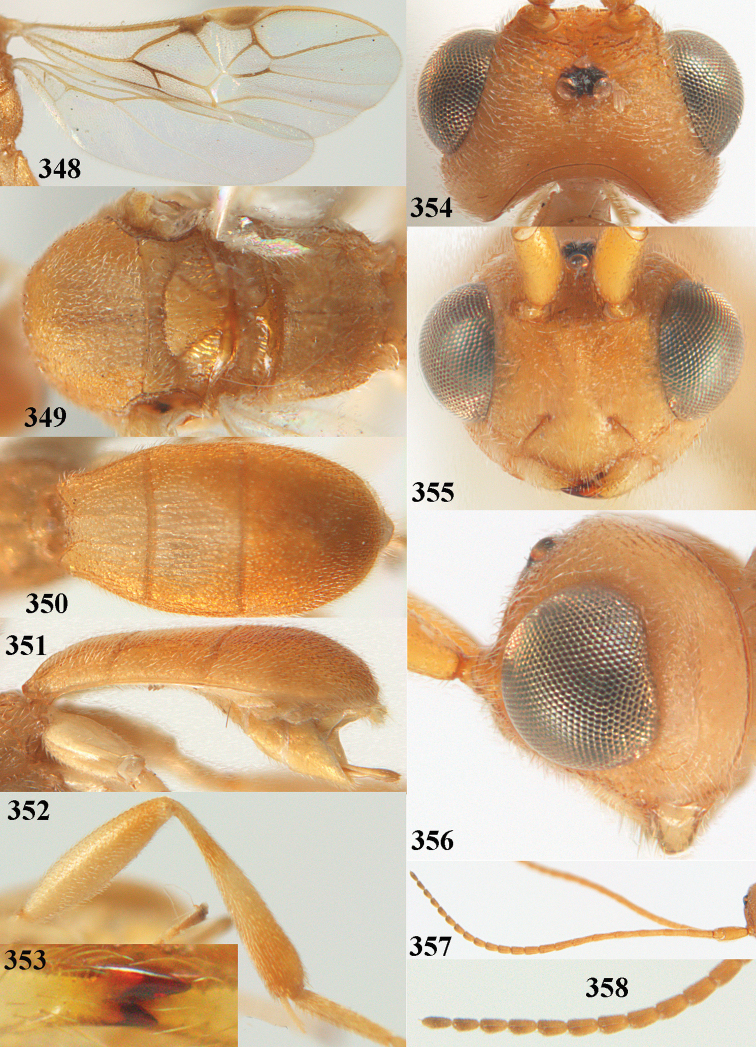
*Phanerotoma spuriserrata* van Achterberg, sp. nov., ♀, holotype **348** wings **349** mesosoma dorsal **350** first–third metasomal tergites dorsal **351** metasoma lateral **352** hind leg lateral **353** mandible ventral **354** head dorsal **355** head anterior **356** head lateral **357** antenna lateral **358** apical third of antenna lateral.

Subapical antennal segments of ♀ rather slender, with erect subapical setae, sixth segment from apex narrowed basally and subapically widened and with small and round protuberances near apex, resulting in a somewhat serrate margin of antenna (Figs 347, 357, 358); antenna of ♀ approx. as long as body and eighth–eleventh segments from apex elongate; POL 0.6–0.8 × diameter of posterior ocellus; temple densely striate and with satin sheen; clypeus much narrower than face, semi-circular, protruding medio-ventrally and with three minute teeth (Fig. 355); length of malar space 0.7–0.8 × basal width of mandible; inner tooth of mandible 0.4–0.5 × as long as apical tooth (Fig. 353); vein 1-Rl of fore wing 1.3–1.4 × length of pterostigma (Fig. 348); distance between apex of wing and vein 1-R1 ca. 0.2 × vein 1-R1; parastigma largely yellow and large; scutellar sulcus wide; vein r 0.6–0.8 × vein 3-SR and almost linearly connected (Fig. 348); vein cu-a of fore wing distinctly inclivous; pterostigma pale yellowish basally and more or less brownish medially; vein 1-M brownish yellow or brown; third metasomal tergite 1.4–1.5 × as long as second tergite and with curved sides; hypopygium without up curved triangle or spine apically; length of fore wing 2.8–3.8 mm. Similar to *P. longiradialis* van Achterberg, 1990, from Iraq, but differs by having subapical antennal segments partly widened near apex (“pseudo-serrate” margin of antenna) and eighth–eleventh antennal segments of ♀ from apex elongate (respectively, cylindrical and shortened in *P. longiradialis*), vein r of fore wing 0.6–0.8 × as long as vein 3-SR (approx. as long), clypeus without three minute teeth medio-ventrally (without distinct teeth) and vein cu-a of fore wing distinctly inclivous (nearly vertical).

##### Description.

Female, holotype, length of body (excluding ovipositor) 4.5 mm; antenna 4.8 mm; fore wing 3.5 mm; visible part of ovipositor sheath 0.3 mm.

***Head*.** Width 1.4 × median length in anterior view and part of head above eye in lateral view 0.3 × height of eye (Fig. 356); antenna with 23 segments and 1.1 × as long as fore wing, segments near apical quarter of antenna elongate and longer than wide, widened subapically because of small round protuberances and seven apical segments rather moniliform (Figs 357, 358) and apical segment without minute spine, third, fourth and penultimate segments 3.8, 3.2 and 2.0 × longer than wide in lateral view, respectively; area of stemmaticum mostly smooth; OOL: diameter of posterior ocellus: POL = 17: 4: 5; length of eye 2.8 × temple in dorsal view (Fig. 354); frons coarsely rugose and rather shiny, with median carina posteriorly; vertex transversely rugose-striate and with satin sheen; temple largely striate and with satin sheen, its median width 0.6 × width of eye in lateral view; face reticulate-rugose and with median ridge dorsally, but no distinct median carina; clypeus mostly smooth, shiny and its width 0.7 × minimum width of face, intertentorial distance 1.2 × minimum distance between clypeus and eye and medio-ventrally with 3 minute teeth (Fig. 355); eye large, strongly convex and in lateral view 1.5 × (measured medially) as wide as temple (Fig. 356), in anterior view its height 0.9 × minimum width of face; upper condyle of mandible below lower level of eyes (Fig. 355); malar space rugose, with satin sheen and as long as basal width of mandible; lower tooth of mandible 0.5 × as long as apical tooth (Fig. 353).

***Mesosoma*** (Figs 347, 349). Length 1.7 × its width in lateral view; side of pronotum only medially and posteriorly rugose, remainder finely rugulose; propleuron posteriorly evenly convex; mesosternum densely granulate and rather matt; mesoscutum densely rugulose; scutellum flat, densely granulate but smooth posteriorly and with satin sheen; notauli not indicated; scutellar sulcus wide and with seven carinae (Fig. 349); metanotum without short median carina anteriorly and narrowly crenulate posteriorly; propodeum coarsely rugose-reticulate, without distinct median and transverse carinae, and latero-posteriorly slightly tuberculate. ***Wings*.** Fore wing 3.0 × longer than its maximum width; length of 1-R1 1.4 × as long as pterostigma; distance between wing apex and vein 1-R1 0.2 × length of vein 1-R1; r issued distinctly beyond middle of pterostigma, nearly linearly connected to 3-SR and 0.7 × 3-SR; 2-SR nearly straight and distally distinctly converging to posterior margin of pterostigma (Fig. 348); SR1 straight; 2-SR+M absent, m-cu interstitial; parastigma large; first discal cell of fore wing much higher than first subdiscal cell; 1-CU1 0.4 × as long as vein 2-CU1, cu-a strongly inclivous and 0.9 × 1-CU1; r:3-SR:SR1 = 7:10:54; 2-SR:3-SR:r-m = 20:10:5; r-m reclivous; 2-M hardly curved (Fig. 348). Hind wing: M+CU:1-M:1r-m = 24:24:12. ***Legs*.** Hind femur with satin sheen, 3.8 × as long as wide and rather widened submedially; hind tibia rather slender (Fig. 352); middle tibia with medium-sized yellowish blister; inner spur of middle tibia 0.4 × its basitarsus; hind coxa mostly smooth and shiny.

***Metasoma*** (Figs 350, 351). Elliptical in dorsal view, 1.9 × as long as wide and 1.1 × as long as mesosoma; first and second tergites densely and rather coarsely longitudinally rugose; metasomal sutures medium-sized; third tergite distinctly convex medially, 1.4 × longer than second tergite and laterally curved, in lateral view rather convex (Fig. 351), largely densely rugulose and with satin sheen, lateral lamella narrow laterally, posteriorly rather wide and not protruding latero-apically, medio-apically truncate; ovipositor sheath widened apically, its visible part 0.1 × as long as fore wing and 0.15 × metasomal carapace and its setose apical part with medium-sized setae and 0.03 × as long as fore wing; hypopygium setose and acute apically (Fig. 351), without up curved triangle apically or apical spine.

***Colour*.** Yellowish brown; apical antennal segments apically and apex of ovipositor sheath brown; stemmaticum blackish; telotarsi, veins 1-CU1, 2-CU1 (but apically yellow), cu-a, r, 3-SR basally and 2-M of fore wing dark brown, other veins (including vein 1-M), parastigma largely and pterostigma (but basally and apically pale yellowish) brown; clypeus, mandible (except dark brown teeth), palpi, pronotum, tegulum and humeral plate, remainder of legs (but hind femur rather brownish except basally and hind tibia subbasally and apically slightly darkened), first and second tergites and metasoma ventrally pale yellowish; wing membrane below veins 1-&2-CU1 and below pterostigma brownish and remainder largely subhyaline.

##### Male.

Similar to female (including shape of hind femur); antennal segments slenderer and usually less serrate and with indistinct subapical protuberances, but sometimes similar to segments in female.

##### Variations.

Length of fore wing of ♀ 3.0–3.8 mm, of ♂ 2.8–3.2 mm; inner tooth of mandible robust and 0.4–0.5 × as long as apical tooth; pterostigma sometimes largely yellowish, but usually brownish medially; vein r of fore wing 0.6–0.8 × vein 3-SR.

##### Biology.

Unknown.

##### Distribution.

Yemen.

##### Etymology.

From *spurius* (Latin for false) and *serra* (Latin for saw), because of the somewhat serrated apical third of the antenna.

#### Phanerotoma stenochora sp. nov.

Taxon classificationAnimaliaHymenopteraBraconidae

DA5F415E-BEDF-54DE-BE82-8DBFB7D6E3FF

http://zoobank.org/678D315C-FBF2-4204-A4FE-9EE40E2AF8C6

Figs 359–362 , 363–373

##### Type material.

***Holotype***, ♀, “**United Arab Emirates**, Sharjah Desert Park (11718), light tr[ap], 30.iv.–21.v.2007, 25°17'N, 55°42'E, A. v. Harten, RMNH’10”. ***Paratypes***: 1♀: Idem, 24.iii.–1.iv.2007; 1♀: Idem, 21.xii.2007–23.i.2008; 1♀: Idem, 29.iii.–6.iv.2005; 1♀: Idem, 6–13.iv.2005; 1♂: “United Arab Emirates, Sharjah (2279), light trap, 30.vi.–21.vii.2005, 25°17'N, 55°42'E, A. v. Harten, RMNH’06”; 1♀: “United Arab Emirates, al-Ajban (11858), light trap, 17.iv.–29.v.2006, 24°36'N, 55°01'E, A. v. Harten, RMNH’10”; 2♀: Idem, 17.x.–9.xi.2005; 1♀: Idem, 28.xii.2005–29.i.2006, Malaise & light trap; 3♀: Idem, 7–28.xii.2006; 2♀: Idem, 25.v.–26.vi.2006, Malaise trap; 2♀: “United Arab Emirates, NARC near Sweihan (1473), light trap, 20–30.iv.2005, 24°24'N, 55°26'E, A. v. Harten, RMNH’05”; 1♀: Idem, 1.ii.–14.iii.2005; 1♀: “United Arab Emirates, Bithnah (3699), at light, 11.xii.2005–18.i.2006, 25°17'N, 55°42'E, A. v. Harten, RMNH’06”; 1♀, 1♂: “United Arab Emirates, SSW of ad-Dhaid (1462), at light & light tr[ap], 23.iv.2005, 25°09'N, 55°48'E, A. v. Harten & K. Szpila, RMNH’06”.

**Figures 359–362.**
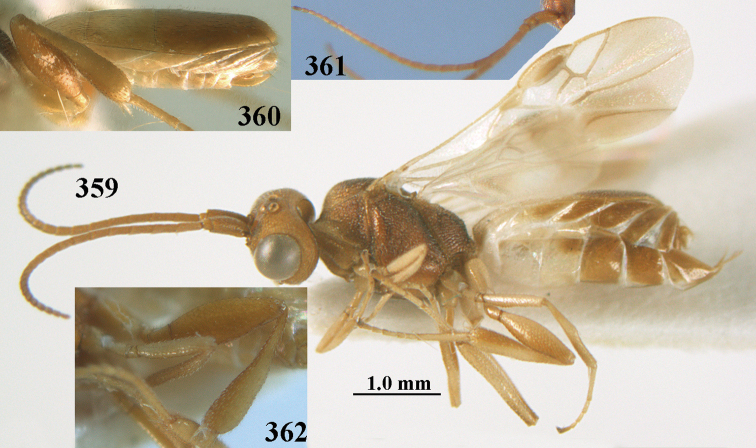
*Phanerotoma stenochora* van Achterberg, sp. nov., ♀, holotype (but **360–362** of ♂, paratype) **359** habitus lateral **360** metasoma lateral **361** antenna lateral **362** hind femur and tibia lateral.

##### Diagnosis.

**Figures 363–373.**
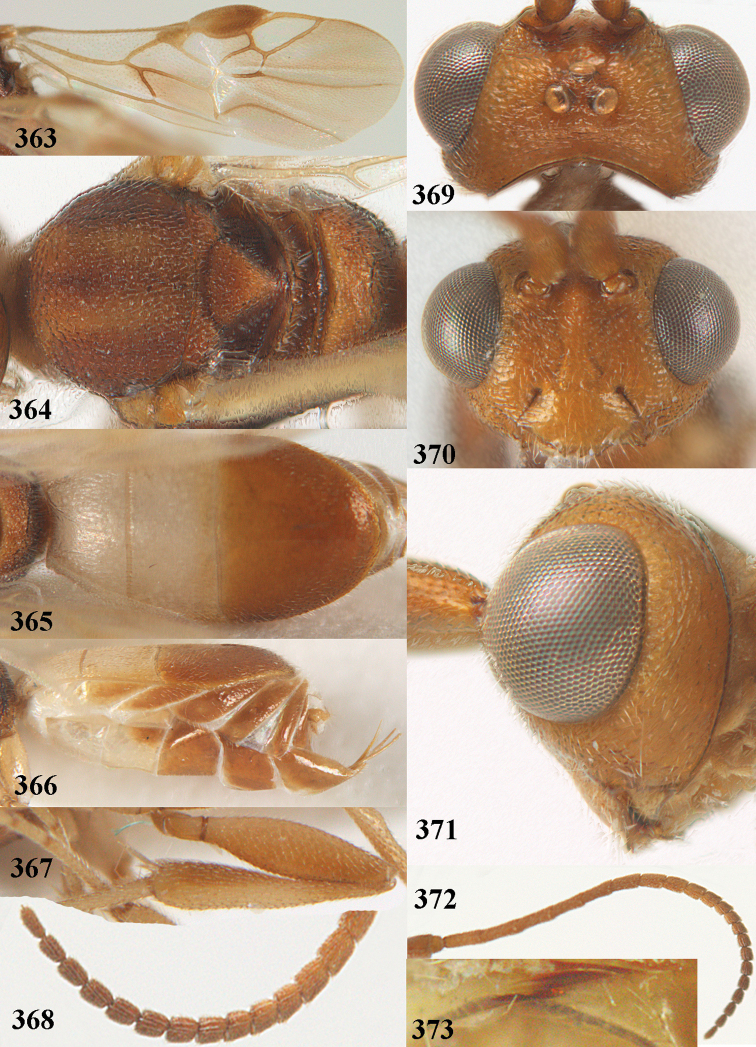
*Phanerotoma stenochora* van Achterberg, sp. nov., ♀, holotype **363** fore wing **364** mesosoma dorsal **365** first–third metasomal tergites dorsal **366** metasoma lateral **367** hind leg lateral **368** apical half of antenna lateral **369** head dorsal **370** head anterior **371** head lateral **372** antenna lateral **373** mandible ventral.

Differs from all other species by having the first discal cell of fore wing as high as first subdiscal cell (Fig. 363), vein 1-R1 of fore wing ca. ½ as long as distance between apex of vein 1-R1 and apex of wing (Fig. 359) and third metasomal tergite shiny, dark brown or brown (contrasting with ivory second tergite), 1.8–2.2 × longer than second tergite and laterally curved to nearly straight, mostly smooth and flat apically (Fig. 365); metasomal sutures very narrow (Fig. 366); malar space long in lateral view, 1.6 × as long as basal width of mandible (Fig. 371); mesoscutum and vertex coarsely rugose-reticulate; inner tooth of mandible 0.2 × as long as apical tooth (Fig. 373); ovipositor sheath needle-shaped and only apically with some setae (Fig. 366). Superficially similar to *P. intermedia* van Achterberg, 1990, from Turkey and Israel, but the eyes are much larger (small in *P. intermedia*), clypeus with three small ventral teeth (absent), medium-sized ocelli (small), first discal cell of fore wing as high as first subdiscal cell (higher) and third tergite mostly smooth (reticulate-rugose).

##### Description.

Female, holotype, length of body (excluding ovipositor) 5.0 mm; antenna 3.3 mm; fore wing 3.5 mm; visible part of ovipositor sheath 0.5 mm (only apex setose).

***Head*.** Width 1.4 × median length in anterior view and part of head above eye in lateral view 0.3 × height of eye (Fig. 371); antenna with 23 segments and slightly shorter than fore wing, near apical third segments slightly widened and approx. as long as wide, narrowed apically and apical segments non-moniliform and apical segment with short spine (Figs 359, 368, 372), third, fourth and penultimate segments 3.2, 3.0 and 1.5 × longer than wide in lateral view, respectively; area of stemmaticum rugose; OOL: diameter of posterior ocellus: POL = 9: 5: 4; length of eye 3.0 × temple in dorsal view (Fig. 369); frons coarsely rugose laterally, mostly smooth medially and without median carina; vertex coarsely transversely rugose and rather shiny; temple densely and finely rugose, rather shiny; face transversely rugose and with median ridge dorsally but no distinct median carina; clypeus mostly smooth and distinctly narrower than face, shiny and with three minute teeth medio-ventrally (Fig. 370); eye rather large, strongly convex and in lateral view 1.9 × (measured medially) temple (Fig. 371), in anterior view 0.8 × minimum width of face; upper condyle of mandible near lower level of eyes (Fig. 370); malar space rugose, shiny and 1.6 × as long as basal width of mandible; lower tooth of mandible 0.2 × as long as apical tooth (Fig. 373).

***Mesosoma*** (Figs 359, 364). Length 1.5 × its width in lateral view; side of pronotum coarsely reticulate-rugose; propleuron posteriorly with smooth tubercle; mesosternum mostly smooth and shiny; mesoscutum and scutellum coarsely reticulate-rugose and rather shiny, short setose; notauli not indicated; scutellar sulcus narrow and with eleven carinae (Fig. 364); metanotum with median carina anteriorly and no tooth posteriorly; propodeum coarsely vermiculate-rugose, without distinct median and transverse carinae, latero-posteriorly not tuberculate. ***Wings*.** Fore wing 2.7 × longer than its maximum width; vein 1-R1 0.5 × as long as pterostigma and approx. half as long as distance between apex of vein 1-R1 and apex of wing (Fig. 359); r issued far beyond middle of pterostigma and 0.3 × 3-SR; 2-SR nearly straight and distally subparallel with posterior margin of pterostigma (Fig. 363); SR1 straight; 2-SR+M short, m-cu just postfurcal; parastigma large; first discal cell of fore wing as high as first subdiscal cell (Fig. 363); 1-CU1 0.2 × as long as vein 2-CU1; r:3-SR:SR1 = 6:7:29; 2-SR:3-SR:r-m = 20:7:10; r-m vertical; 2-M slightly curved; basal and subbasal cell rather sparsely setose. Hind wing: M+CU:1-M:1r-m = 27:25:10. ***Legs*.** Hind femur shiny, 3.5 × as long as wide and widened submedially; hind tibia rather robust (Fig. 367); middle tibia with ivory blister; inner spur of middle tibia 0.4 × its basitarsus; hind coxa mostly smooth and shiny.

***Metasoma*** (Figs 365, 366). Elliptical in dorsal view, 1.7 × as long as wide and 1.4 × as long as mesosoma; first and second tergites superficially and very finely rugulose; third tergite 1.9 × longer than second tergite and laterally curved, in lateral view rather flat, mostly smooth and shiny and medio-basally superficially rugulose (Fig. 365), lateral lamella narrow, not protruding latero-apically and medio-apically truncate and medium-sized; ovipositor sheath narrow, needle-shaped, its visible part 0.15 × as long as fore wing and 0.24 × metasomal carapace and only its apex with small cluster of long setae; hypopygium apically with short and moderately wide bent up triangle (Fig. 366), without apical spine and with medium-sized setae.

***Colour*.** Head and antenna (but apically darkened) yellowish brown; palpi, mandible (except dark brown teeth), tegulae and legs pale yellowish; mesosoma largely brown, but mesosternum and imaginary notaulic courses yellow; first and second tergites and ventral half of metasoma ventrally ivory; remainder of metasoma rather dark brown; pterostigma brownish but basally pale yellowish (Fig. 363); wing membrane basally and marginal cell hyaline, remainder of apical half of fore wing largely brownish; parastigma, veins 1-M, 2-CU1 (except basally) and m-cu of fore wing pale yellow and veins r, 1-CU1, cu-a, 2-SR, 3-SR and 2-M brown; ovipositor sheath evenly brown.

##### Male.

Similar to female but hind femur inflated (Fig. 362), antennal segments slender and elongate (Fig. 361), and veins r and 1-M of fore wing widened and latter curved; vein 3-SR 2.7 × as long as vein r; metasoma very shiny and mostly smooth (Fig. 360).

##### Variations.

Length of fore wing of ♀ 2.7–3.9 mm, of ♂ 2.7 mm; third metasomal tergite 1.8–2.2 × longer than second tergite, dark brown or brown, curved to nearly straight laterally; vein 3-SR 1.1–2.7 × as long as vein r; hind femur largely and apex of hind tibia sometimes dark brown.

##### Biology.

Unknown.

##### Distribution.

United Arabian Emirates.

##### Etymology.

Named after the narrow marginal cell of the fore wing (*stenos* is Greek for narrow and *chora* is Greek for room or space).

#### Phanerotoma vanharteni sp. nov.

Taxon classificationAnimaliaHymenopteraBraconidae

150EC2F9-01B5-54BA-9D8C-7DC26D47A7FF

http://zoobank.org/6645EAB3-EBA2-4A80-BA28-22684537E726

Figs 374–376 , 377–386

##### Type material.

***Holotype***, ♀ (RMNH), ♀, “**Yemen**: Al Kowd (8136), ix.2003, light trap, A. v. Harten & S. Al Haruri, RMNH’03”. ***Paratypes***: 2♀: Idem, v.–vi.2000; 1♀: Idem, x.2000; 1♀: Idem, 27–31.vii.2001; 1 ♂: “Yemen (6628), Ta’izz, light trap, vi.2002, A. van Harten & A.R. Al Yarimi, RMNH”; 2♀: “**United Arab Emirates**, NARC near Sweihan (1473), light trap, 28.iii.–2.iv.2005, 24°24'N, 55°26'E, A. v. Harten, RMNH’05”.

**Figures 374–376.**
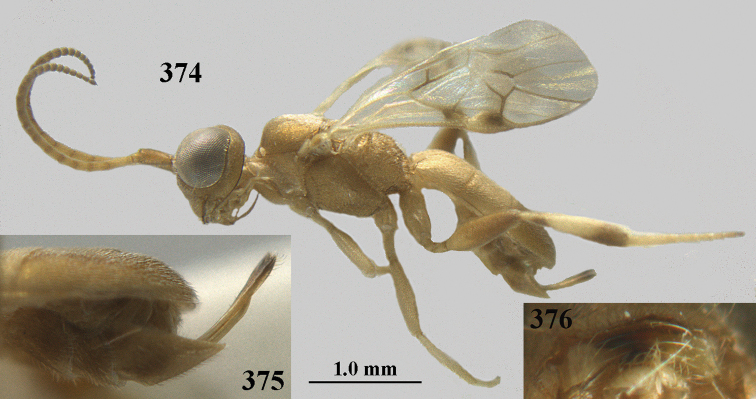
*Phanerotoma vanharteni* van Achterberg, sp. nov., ♀, holotype **374** habitus lateral **375** apical half of metasoma lateral **376** mandible ventral.

##### Diagnosis.

Apical antennal segments of ♀ short and distinctly moniliform (Figs 374, 382, 386), sixth segment from apex narrowed basally and subapically widened (Fig. 382); upper condyle of mandible near lower level of eyes (Fig. 383); temple narrow in lateral view (median width of temple 0.4–0.5 × width of eye; Fig. 385); width of clypeus 0.9 × minimum width of face (Fig. 383); height of eye in anterior view 1.3 × minimum width of face; face densely sculptured, rather matt and dorsally often with fine median carina (Fig. 383), but sometimes superficially sculptured, without median carina and shiny; hind tibia subbasally usually partly dark brown; inner tooth of mandible medium-sized (Fig. 376); vein r of fore wing 0.8–1.1 × vein 3-SR; hypopygium of ♀ straight apically in lateral view (Figs 375, 380), without up curved apical triangle or spine-like protuberance.

**Figures 377–386.**
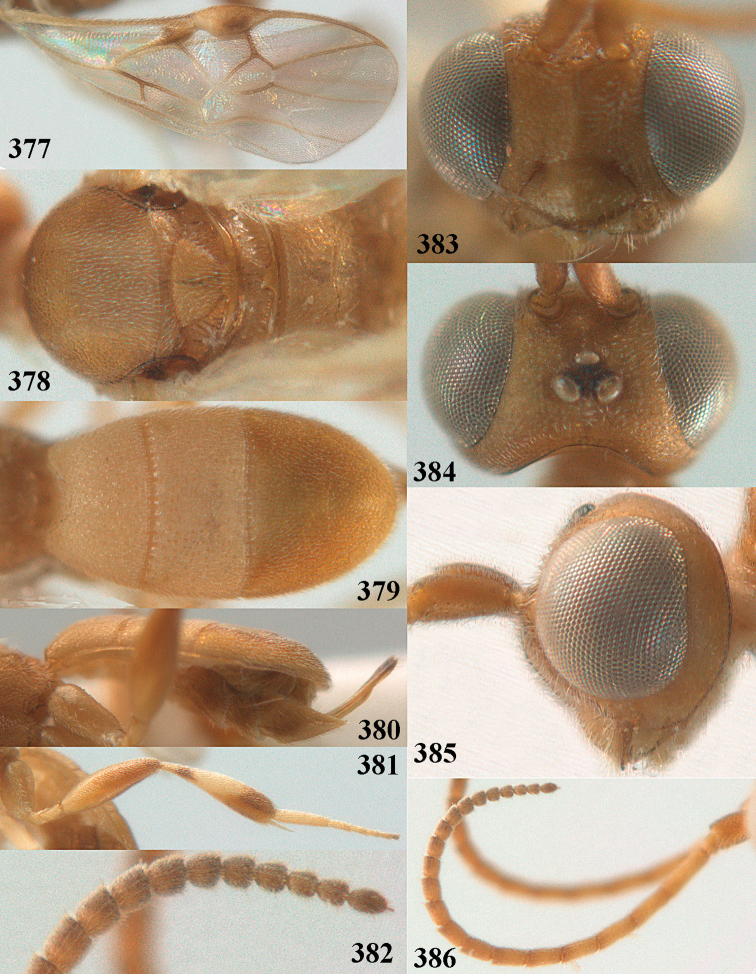
*Phanerotoma vanharteni* van Achterberg, sp. nov., ♀, holotype **377** fore wing **378** mesosoma dorsal **379** first–third metasomal tergites dorsal **380** metasoma lateral **381** hind leg lateral **382** apical third of antenna lateral **383** head anterior **384** head dorsal **385** head lateral **386** antenna lateral.

##### Description.

Female, holotype, length of body (excluding ovipositor) 3.5 mm; antenna 2.5 mm; fore wing 2.5 mm; visible part of ovipositor sheath 0.5 mm, only apically with erect setae.

***Head*.** Width 1.6 × median length in anterior view and part of head above eye in lateral view 0.25 × height of eye (Fig. 385); antenna with 23 segments and as long as fore wing, segments near apical quarter of antenna longer than wide, somewhat serrate and widened subapically because of small round protuberances and nine apical segments moniliform (Figs 382, 386) and apical segment with spine, third, fourth and penultimate segments 3.4, 2.8 and 1.1 × longer than wide in lateral view, respectively; area of stemmaticum coriaceous; OOL: diameter of posterior ocellus: POL = 7: 3: 5; length of eye 5.4 × temple in dorsal view (Fig. 384); frons rugose laterally, rugulose medially and with satin sheen, without median carina posteriorly; vertex rugose but posteriorly striate and with satin sheen; temple densely striate and rather matt, its median width 0.4 × width of eye in lateral view; face densely sculptured, rather matt and dorsally with fine median carina; clypeus largely very finely coriaceous, with satin sheen and its width 0.9 × minimum width of face, intertentorial distance 3.0 × minimum distance between clypeus and eye and medio-ventrally with three minute lobes (Fig. 383); eye large, strongly convex, in anterior view its height 1.3 × minimum width of face; upper condyle of mandible near lower level of eyes (Fig. 383); malar space rugose, with satin sheen and its length 0.5 × basal width of mandible; lower tooth of mandible 0.5 × as long as apical tooth (Fig. 376).

***Mesosoma*** (Figs 374, 378). Length 1.5 × its width in lateral view; side of pronotum granulate dorsally, rugose medially and remainder mostly smooth; propleuron posteriorly evenly convex and protruding towards medial sulcus; mesosternum densely granulate and matt; mesoscutum densely rugulose; scutellum flat, densely granulate but smooth posteriorly and with satin sheen; notauli not indicated; scutellar sulcus wide and with six carinae (Fig. 378); metanotum with median carina anteriorly, flat posteriorly and narrowly serrate posteriorly; propodeum granulate-rugulose anteriorly and remainder coarsely rugose-reticulate, transverse carina distinct, without median carina, antero-dorsal face long and latero-posteriorly slightly tuberculate. ***Wings*.** Fore wing 2.6 × longer than its maximum width; length of 1-R1 1.4 × as long as pterostigma; distance between wing apex and vein 1-R1 0.2 × length of vein 1-R1; r issued distinctly beyond middle of pterostigma, nearly linearly connected to 3-SR and 0.9 × 3-SR; 2-SR straight and distally slightly converging to posterior margin of pterostigma (Fig. 377); SR1 straight; 2-SR+M present, m-cu antefurcal; parastigma large; 1-CU1 0.5 × as long as vein 2-CU1, cu-a slightly inclivous and 0.8 × 1-CU1; r:3-SR:SR1 = 10:11:47; 2-SR:3-SR:r-m = 20:11:7; r-m reclivous; 2-M slightly curved (Fig. 377). Hind wing: M+CU:1-M:1r-m = 23:19:10. ***Legs*.** Hind femur rather dull, 3.5 × as long as wide and widened submedially; hind tibia wide medially (Fig. 381); middle tibia with small ivory blister; inner spur of middle tibia 0.55 × its basitarsus; hind coxa largely superficially granulated and rather shiny.

***Metasoma*** (Figs 379, 380). Elliptical in dorsal view, 1.9 × as long as wide and 1.3 × as long as mesosoma; first and second tergites coarsely longitudinally rugose; metasomal sutures medium-sized and deep; third tergite distinctly convex medially, 1.7 × longer than second tergite and laterally curved, in lateral view rather convex (Fig. 380), largely densely reticulate-rugulose and with satin sheen, lateral lamella narrow laterally and posteriorly, not protruding latero-apically and medio-apically truncate; ovipositor sheath parallel-sided and rather narrow apically, its visible part 0.15 × as long as fore wing and 0.3 × metasomal carapace and only apically with medium-sized setae; hypopygium setose and acute apically, without up curved triangle or spine apically (Fig. 380).

***Colour*.** Yellowish brown; apical antennal segments apically and apex of ovipositor sheath brown; stemmaticum dark brown; parastigma largely, veins 1-CU1, base of 2-CU1, cu-a, r, 2-SR, 3-SR, SR1 and 2-M of fore wing dark brown, other veins (including vein 1-M), and pterostigma basally and apically, pale yellowish; apical half of pterostigma partly dark brown; clypeus, mandible (except dark brown teeth), palpi, pronotal side, tegulum, mesoscutum medio-posteriorly, legs (but hind femur rather brownish except basally and hind tibia subbasally and apically dark brown), first and second tergites and metasoma baso-ventrally pale yellowish; humeral plate brownish; wing membrane below veins 1-&2-CU1 and below pterostigma brownish and remainder largely subhyaline.

##### Male.

Similar to female (including shape of hind femur and tibia); antennal segments slenderer and weakly serrate and with minor subapical protuberances; median width of temple 0.55 × width of eye in lateral view.

##### Variations.

Length of fore wing of ♀ 3.0–3.8 mm, of ♂ 2.8 mm; median width of temple 0.4–0.5 × width of eye in lateral view; dark part of hind tibia dark brown or brown; vein r of fore wing linear with vein 3-SR or rather angled, 0.8–1.1 × vein 3-SR; face often densely sculptured, rather matt or with satin sheen and dorsally often with fine median carina (Fig. 383), but female from UAE has face superficially sculptured, without median carina and shiny.

##### Biology.

Unknown.

##### Distribution.

United Arab Emirates, Yemen.

##### Etymology.

Named in honour of Tony van Harten (editor of the series ‘Arthropod Fauna of the UAE’) for his extraordinary efforts to make extensive collections of insects and species in hardly investigated habitats.

#### Phanerotomella

Taxon classificationAnimaliaHymenopteraBraconidae

Szépligeti, 1900

83B3421F-6F2E-54D0-9B95-85C0BE9CE8D1

Figs 387–390 , 391–401

Phanerotomella Szépligeti, 1900: 59; Shenefelt 1973: 929; van Achterberg 1990: 7; Zettel 1989: 18, 22–26, 58–59, 68–70; Tobias 2000: 439–440; Chen and Ji 2003: 229–231; Ahmad and Shujauddin 2003: 353; Braet et al. 2012: 24–25. Type-species: Phanerotomella longipes Szépligeti, 1900 (examined). Designated by Viereck 1914: 115.
Plesiosphaeropyx Cameron, 1912: 82, 84. Type-species: Plesiosphaeropyx albipalpis Cameron, 1912. Monotypic. Synonymised by De Saeger (1948).

##### Diagnosis.

**Figures 387–390.**
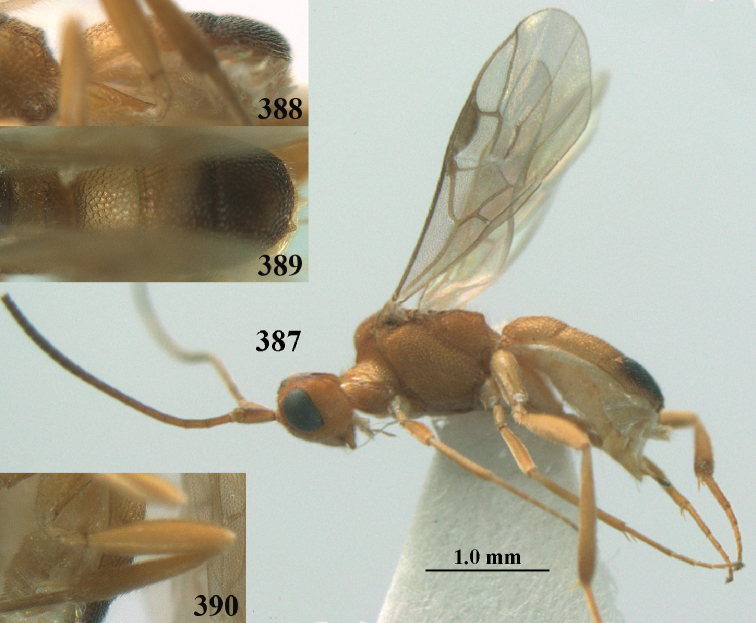
*Phanerotomella yemenitica* van Achterberg, sp. nov., ♀, holotype (but **388–390** of ♂, paratype) **387** habitus lateral **388** metasoma lateral **389** metasoma dorsal **390** hind femur and tibia lateral.

**Figures 391–401.**
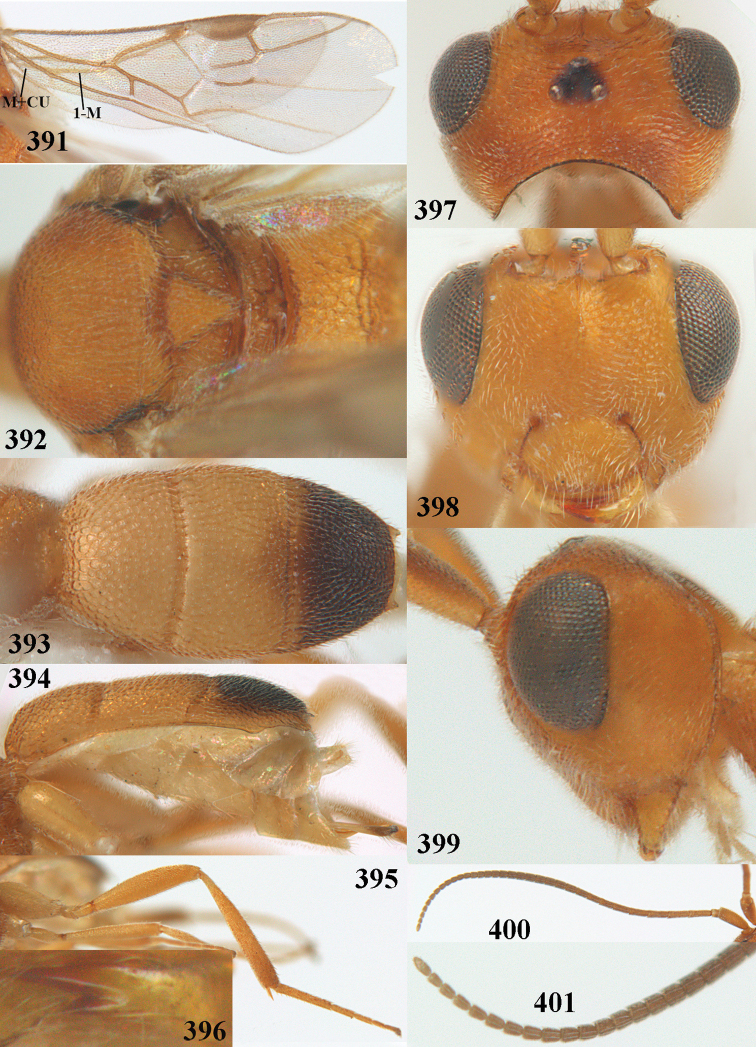
*Phanerotomella yemenitica* van Achterberg, sp. nov., ♀, holotype **391** wings **392** mesosoma dorsal **393** first–third metasomal tergites dorsal **394** metasoma lateral **395** hind leg lateral **396** mandible ventral **397** head dorsal **398** head anterior **399** head lateral **400** antenna lateral **401** apical half of antenna lateral.

Antennal segments (24–)30–60; eyes glabrous; vein 2-R1 of fore wing present (Fig. 391); second submarginal cell more or less triangular and often petiolate (Fig. 391); vein CU1b of fore wing absent, resulting in an open first subdiscal cell (Fig. 387); pterostigma usually comparatively slender; vein r of hind wing absent; vein 1-SR+M of fore wing present; carapace with distinct transverse sutures (Fig. 393); third metasomal tergite without slender lateral teeth, at most corners triangularly protruding latero-posteriorly (Fig. 393).

##### Distribution.

C & SE Europe, East Palaearctic, Afrotropical (including Malagasy) and Indo-Australian regions.

#### Phanerotomella yemenitica sp. nov.

Taxon classificationAnimaliaHymenopteraBraconidae

B1C04B33-6CF6-56C9-8661-726AA5C26FB4

http://zoobank.org/F90C28B1-1CB9-4926-B15B-CF19AA4C6FCF

Figs 387–390 , 391–401

##### Type material.

***Holotype***, ♀ (RMNH), “**Yemen** (no. 2910), Ta’izz, light trap, 5.i.–2.ii.1998, A. van Harten, RMNH’98”. ***Paratypes***: 2♀: with same data as holotype; 1♀: Idem, 26–28.v.1998; 1♀: Idem, i.2000; 2♀: Idem, v.2000; 1♀: Idem, viii.2000; 2♀: Idem, x.2001; 1♀: Idem, vi.2002; 2♂: “Yemen (6394), Al Lahima, 14.xi.2001–6.iii.2002, Mal. trap, A. v. Harten, RMNH’02”; 1♂: Idem, 1.i.–9.iv.2001; 1♂: Idem, 9.iv.–5.vi.2001; 1♀: Idem, 5.vi.–24.vii.2001; 1♀: Idem, 17.ix.–14.xi.2001; 4♀: “Yemen (6090), Al Kadan, x.2001, light trap, A. v. Harten & T. Abdul-Haq, RMNH’03”; 1♀: Idem, v.2002.

##### Comparative diagnosis.

The new species runs in the key by Braet et al. (2012) to *P. capensis* Zettel, 1989 and *P. aurea* Zettel, 1989, but the new species differs from both species by having the third metasomal tergite dark brown (brownish yellow in both species), inner spur of middle tibia 0.4 × as long as middle basitarsus (0.5–0.6 ×) and subapical antennal segments of ♀ stout (slenderer). It shares with *P. aurea* Zettel the distinctly developed pair of apical teeth of the third tergite, but the face and clypeus are matt (distinctly shiny in *P. aurea*).

##### Description.

Female, holotype, length of body (excluding ovipositor) 3.1 mm; of antenna 3.7 mm; of fore wing 2.7 mm; visible part of ovipositor sheath 0.3 mm (setose part 0.1 mm).

***Head*.** Width 1.3 × median length in anterior view and part of head above eye in lateral view 0.3 × height of eye (Fig. 399); antenna with 34 segments and 1.4 × longer than fore wing, twelfth–24^th^ segments widened and shortened, gradually narrowed apically, apical segments non-moniliform and slightly longer than wide (Fig. 401), third, fourth and penultimate segments 3.4, 3.2 and 1.3 × longer than wide in lateral view, respectively; area of stemmaticum transversely striate; OOL: diameter of posterior ocellus: POL = 26: 7: 10; length of eye 1.3 × temple in dorsal view (Fig. 397); frons aciculate but dorso-laterally rugose and with median carina; vertex reticulate-rugose with fine coriaceous background sculpture, short setose; temple densely rugose and with satin sheen; face rugose and with distinct median ridge, dorsally connect to median carina; clypeus punctate and with shiny interspaces, truncate medio-ventrally (Fig. 398); eye medium-sized in lateral view (Fig. 399), in anterior view 0.8 × minimum width of face (Fig. 398); upper condyle of mandible far below lower level of eyes (Fig. 398); malar space rugose and 1.5 × as basal width of mandible; lower tooth of mandible 0.3 × as long as apical tooth (Fig. 396).

***Mesosoma*** (Figs 387, 392). Length 1.5 × its width in lateral view; side of pronotum reticulate-rugose; mesoscutum reticulate-rugulose with granulate background, densely setose; notauli slightly indicated but not well differentiated; scutellar sulcus medium-sized and with four short crenulae (Fig. 392; scutellum triangular, reticulate-punctate; metanotum with short median carina anteriorly and small tooth posteriorly; propodeum coarsely reticulate, without median carina, with irregular transverse carina connected to tuberculate corners (Fig. 392). ***Wings*.** Fore wing 2.9 × longer than its maximum width; length of 1-R1 1.1 × pterostigma; r issued far beyond middle of pterostigma and 3 × petiole of second submarginal cell; 2-SR only basally weakly bent and nearly parallel with posterior margin of pterostigma (Fig. 391); SR1 straight; 2-SR+M nearly absent because of slightly postfurcal m-cu; parastigma small and brown; 1-CU1 0.4 × as long as vein 2-CU1; r:2-SR:SR1 = 10:25:47; r-m vertical; 2-M weakly curved (Fig. 391). Hind wing: M+CU:1-M:1r-m = 20:23:12. ***Legs*.** Hind femur 4.8 × as long as wide; middle tibia without ivory blister; inner spur of middle tibia 0.4 × its basitarsus; hind coxa smooth and shiny dorsally, laterally superficially punctate and with satin sheen; hind tibia slender (Fig. 395).

***Metasoma*** (Figs 393, 394). Elliptical in dorsal view, 1.8 × as long as wide and 1.1 × as long as mesosoma; first–third tergites densely reticulate-rugose; third tergite 1.1 × longer than second tergite, mainly densely and finely reticulate-rugulose and truncate medio-posteriorly (Fig. 394), lateral lamella tooth-shaped protruding latero-apically and medium-sized, weakly sinuate medio-apically (Fig. 394); setose part of ovipositor sheath 0.04 × as long as fore wing and visible part of ovipositor sheath 0.11 × as long as fore wing and 0.14 × metasomal carapace; hypopygium with apically acute triangular lobe and with long setae (Fig. 394).

***Colour*.** Yellowish brown; palpi, mandible (except dark brown teeth), tegulae, legs (but hind tibia slightly darkened apically) and metasoma ventrally pale yellow or ivory; flagellum brown; stemmaticum and third tergite dark brown; pterostigma rather dark brown with vague subhyaline basal spot (Fig. 391); wing membrane evenly slightly infuscate; parastigma and vein 1-M (as other veins) pale brown.

##### Male.

Very similar to female, but antenna slender medially (Figs 388–390).

##### Variations.

Length of fore wing of ♀ 2.3–2.7 (of ♂ 2.1–2.2) mm; antenna of ♀ with 33 (1), 34 (5) or 35 (2) segments, of ♂ with 33(1) or 34 (2) segments; sometimes mesoscutum (except medio-posteriorly), scutellum (except disc), metanotum and second tergite medio-posteriorly more or less dark brown.

##### Biology.

Unknown.

##### Distribution.

Yemen.

##### Etymology.

Named after the country of origin of the type series, Yemen.

## Supplementary Material

XML Treatment for Phanerotoma

XML Treatment for Phanerotoma angusticrus

XML Treatment for Phanerotoma artocornuta

XML Treatment for Phanerotoma aspidiota

XML Treatment for Phanerotoma bilinea

XML Treatment for Phanerotoma brunneivena

XML Treatment for Phanerotoma caudatoides

XML Treatment for Phanerotoma ejuncida

XML Treatment for Phanerotoma flavivena

XML Treatment for Phanerotoma glabritemporalis

XML Treatment for Phanerotoma graciloides

XML Treatment for Phanerotoma granulata

XML Treatment for Phanerotoma hellyeri

XML Treatment for Phanerotoma latifemorata

XML Treatment for Phanerotoma lepta

XML Treatment for Phanerotoma leucobasis

XML Treatment for Phanerotoma longivena

XML Treatment for Phanerotoma masiana

XML Treatment for Phanerotoma mesocellata

XML Treatment for Phanerotoma microdonta

XML Treatment for Phanerotoma micrommata

XML Treatment for Phanerotoma ocularis

XML Treatment for Phanerotoma permixtellae

XML Treatment for Phanerotoma robusta

XML Treatment for Phanerotoma sculptilis

XML Treatment for Phanerotoma signifera

XML Treatment for Phanerotoma spuriserrata

XML Treatment for Phanerotoma stenochora

XML Treatment for Phanerotoma vanharteni

XML Treatment for Phanerotomella

XML Treatment for Phanerotomella yemenitica
